# Differential CMS-Related Expression of Cell Surface Carbonic Anhydrases IX and XII in Colorectal Cancer Models—Implications for Therapy

**DOI:** 10.3390/ijms24065797

**Published:** 2023-03-18

**Authors:** Arne Rotermund, Sarah Brandt, Martin S. Staege, Jana Luetzkendorf, Lutz P. Mueller, Thomas Mueller

**Affiliations:** 1 Department of Internal Medicine IV (Hematology/Oncology), Medical Faculty, Martin Luther University Halle-Wittenberg, 06120 Halle, Germany; 2 Department of Surgical and Conservative Pediatrics and Adolescent Medicine, Medical Faculty, Martin Luther University Halle-Wittenberg, 06120 Halle, Germany

**Keywords:** carbonic anhydrases, colorectal cancer, consensus molecular subtypes, CMS, SLC-0111, targeted inhibition of carbonic anhydrases, chemotherapy

## Abstract

Tumor-associated carbonic anhydrases IX (CAIX) and XII (CAXII) have long been in the spotlight as potential new targets for anti-cancer therapy. Recently, CAIX/CAXII specific inhibitor SLC-0111 has passed clinical phase I study and showed differential response among patients with colorectal cancer (CRC). CRC can be classified into four different consensus molecular subgroups (CMS) showing unique expression patterns and molecular traits. We questioned whether there is a CMS-related CAIX/CAXII expression pattern in CRC predicting response. As such, we analyzed transcriptomic data of tumor samples for CA9/CA12 expression using Cancertool. Protein expression pattern was examined in preclinical models comprising cell lines, spheroids and xenograft tumors representing the CMS groups. Impact of CAIX/CAXII knockdown and SLC-0111 treatment was investigated in 2D and 3D cell culture. The transcriptomic data revealed a characteristic CMS-related CA9/CA12 expression pattern with pronounced co-expression of both CAs as a typical feature of CMS3 tumors. Protein expression in spheroid- and xenograft tumor tissue clearly differed, ranging from close to none (CMS1) to strong CAIX/CAXII co-expression in CMS3 models (HT29, LS174T). Accordingly, response to SLC-0111 analyzed in the spheroid model ranged from no (CMS1) to clear (CMS3), with moderate in CMS2 and mixed in CMS4. Furthermore, SLC-0111 positively affected impact of single and combined chemotherapeutic treatment of CMS3 spheroids. In addition, combined CAIX/CAXII knockdown and more effective treatment with SLC-0111 reduced clonogenic survival of CMS3 modelling single cells. In conclusion, the preclinical data support the clinical approach of targeted CAIX/CAXII inhibition by showing linkage of expression with response and suggest that patients with CMS3-classified tumors would most benefit from such treatment.

## 1. Introduction

Hypoxia in solid tumors has long been established as an essential factor for tumor progression and tumor malignancy and, therefore, presents a promising strategy in tumor therapy [1]. As intratumoral hypoxia leads to severe metabolic reprogramming, e.g., in pathways such as the Krebs cycle, fatty acid synthesis and the respiratory chain, a metabolic shift towards anaerobic glycolysis and excessive production of acidic metabolites such as lactate and protons (H^+^) is commonly observed in many different tumors in order to meet the energy demands of the fast-growing tumor tissue, even in low-oxygen environments [2]. This metabolic reprogramming is partly induced via the hypoxia-inducible factor 1 alpha (HIF1α) pathway, which supports the shift towards glycolysis but also plays a role in several other key factors of tumor progression, e.g., invasion and metastasis, genomic instability and pH homeostasis [3]. Due to this metabolic shift, physiological homeostasis between intracellular and extracellular space changes drastically inside the tumor as the extracellular pH (pHe) is acidified towards a pH of below 7 while the intracellular pH (pHi) slightly alkalinizes [4]. Alteration in pH, especially in the tumor microenvironment (TME), is further supported by loss of tumor suppressor genes and overexpression of oncogenes, inhibiting the physiological mechanism of pH homeostasis even further [2]. This disruption in pH homeostasis is not exclusively found in highly hypoxic tissue as tumor cells in close proximity to blood vessels exposed to only moderate levels of hypoxia also show strong imbalance in pH homeostasis, therefore solidifying acidosis as a hypoxia-independent factor driving tumor progression [5]. Tumor cells greatly benefit from this pH alteration as an acidified pHe inhibits immune functions [6], supports selection of more malignant tumor cells [7], supports degradation of the extracellular matrix [8], supports angiogenesis [9] and inhibits efficacy of anti-tumor therapy as many drugs have pH-titrable groups [10,11,12]. Due to the permanently slightly alkaline pHi, intracellular energy production remains largely unaffected by excessive production of acidic metabolites, which aids tumor growth and cell survival [13].

The dysregulated balance between pHi and pHe is maintained by complex machinery consisting of several membrane transporters, e.g., Na^+^/H^+^-exchangers (NHEs) or sodium bicarbonate cotransporters (NBCs), as well as membrane-associated carbonic anhydrases IX and XII (CAIX/CAXII) and intracellular carbonic anhydrase II. Both the membrane transporters and CAIX/CAXII are upregulated via HIF1α and form a functional complex, a so called “Metabolon”, which facilitates extrusion of acidic metabolites, such as lactate, in highly hypoxic and CO_2_ in moderately hypoxic tumor tissue and ensures uptake of bicarbonate (HCO_3_^−^), which is used to buffer intracellular protons [14,15,16]. As both hypoxia and acidosis are essential factors driving tumor progression, both have already been discussed as potential targets for anti-tumor therapy [9,17]. HIF1α especially has long been a gene of interest as it is induced by both hypoxia and acidosis [18] and plays a role in many aspects of tumor development. However, due to its physiological functions, direct inhibition of HIF1α is impossible. Therefore, inhibition of HIF1α target genes seems to be a better approach for anti-tumor therapy.

Two of those genes are CA9 [19,20] and CA12 [21,22], both zinc metalloenzymes catalyzing reversible hydration of CO_2_ to H^+^ and HCO_3_^−^. In comparison to other carbonic anhydrases, especially, expression of CAIX is low in healthy tissue but high in several carcinomas, including carcinomas of the colon [23,24], breast [25], head and neck [26], kidney [27] and bladder [28], making them an interesting target for anti-tumor therapy [29]. Aside from their described role in pH-regulation, CAIX and CAXII also play an important role in chemotherapeutic resistance [30,31], tumor cell migration [32], tumorigenesis [33], cell adhesion [34,35] as well as tumor growth and survival [36,37]. Furthermore, CAIX and CAXII are prognostic markers in several carcinomas [38,39,40,41,42,43,44,45]. Due to their many functions in cancer, several studies on inhibition of CAIX and CAXII have been conducted so far, including development of monoclonal antibodies [46] and small-molecule inhibitors [47], some of which have already entered clinical trials. One small-molecule inhibitor, SLC-0111, an ureido-substituted benzenesulfonamide, has recently passed clinical phase I dose escalation study and showed a promising safety and tolerability profile in patients with previously treated advanced solid tumors [48]. Interestingly, one among four patients with colorectal cancer exhibited stable disease for prolonged time.

Colorectal cancer (CRC) represents a heterogeneous disease both from a molecular and clinical perspective. With the establishment of consensus molecular subtypes (CMS) by an international consortium, CRC was classified into four different molecular subgroups and an unclassified/mixed group showing distinguishing features: CMS1 (MSI immune, 14%), hypermutated, microsatellite unstable and strong immune activation; CMS2 (canonical, 37%), epithelial, marked WNT and MYC signaling activation; CMS3 (metabolic, 13%), epithelial and evident metabolic dysregulation; and CMS4 (mesenchymal, 23%), prominent TGF-beta activation, stromal invasion and angiogenesis [49]. As the different subgroups are very much distinct from each other, several studies were conducted examining the differences between those subgroups regarding different pathophysiological traits, including chemotherapeutic efficacy [50], tumor location [51], local anti-tumor immune response [52], tumor budding [53] and regarding their clinical predictive and prognostic value [54,55]. Notably, the CMS classification of CRC is recapitulated in preclinical model systems, enabling investigations to uncover new targets and test new therapy approaches [56,57,58]. Accordingly, we used our preclinical CRC models comprising cell lines, spheroids and xenograft tumors, which were classified into the four different CMS groups by applying the CMScaller [59].

The aim of this study was to analyze expression of CAIX and CAXII with special respect to CMS classification of CRC and investigate their specific role regarding differential tumor biological and therapeutic aspects.

## 2. Results

### 2.1. Differential Molecular-Subtype-Associated Expression of CA9 and CA12 in Colorectal Cancer

**Figure 1:**
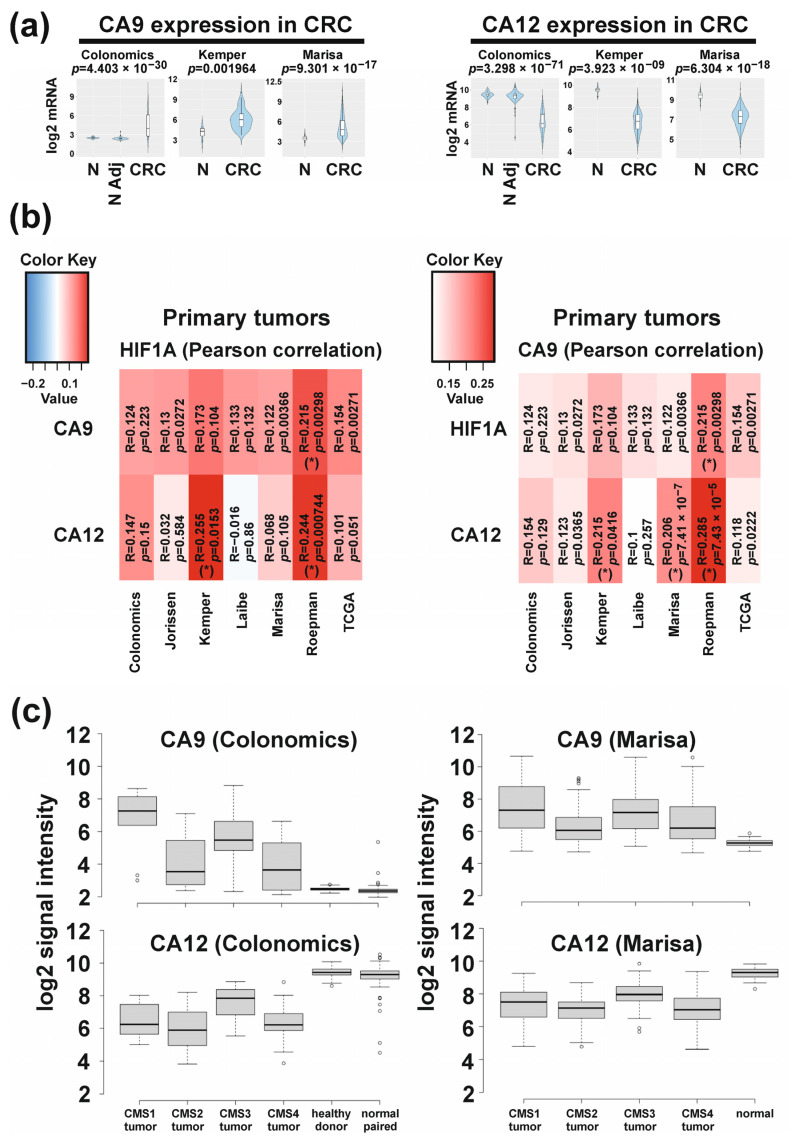
Analyses of expression and correlation of CA9, CA12 and HIF1α on transcriptomic levels in patient tumor samples using Cancertool [60]. (**a**): Expression of CA9 and CA12 in relation to normal or normal-adjacent tissue in 3 out of 7 CRC datasets. Other 4 datasets did not contain normal tissue and could, therefore, not be included for this analysis. (N = normal tissue; N Adj = normal tissue adjacent to tumor; CRC = colorectal carcinoma) (**b**): Heat maps of correlation analyses based on HIF1α (left) and CA9 (right) in 7 datasets showing overall positive correlation of both CAs to HIF1α and both CAs to each other with different stringency depending on datasets. (**c**): CMS-related expression of CA9 and CA12. Depicted are 2 representative datasets (Colonomics, GSE44076; Marisa, GSE39582). The other 5 datasets can be found in Figure S1 (Supplementary Materials). Tumor samples of each dataset were classified using the CMScaller [59]. Schemes of (**a**,**b**) were created by Cancertool and modified to improve the lettering. Heat maps: in every cell, the corresponding R and *p*-values of the analysis are shown. The color of each cell represents its correlation R value, being red towards 1 and blue towards −1. Correlations with *p*-value ≤ 0.05 and |R| ≥ 0.2 are indicated with (*).

Based on the data of the phase 1 study showing differential response to CAIX/XII specific inhibitor SLC-0111 among patients with colorectal cancer [48], we questioned whether there is a characteristic differential expression pattern of both CAs in colorectal tumors, which could explain the differential response. To this end, we used Cancertool [60] to investigate gene expression of CA9 and CA12 in patient tumor samples. Cancertool is an online bioinformatics platform performing expression-, correlation- and gene-enrichment analyses based on seven different colorectal cancer transcriptomic datasets. Three of the seven datasets also contain normal tissue. The summarized data established with Cancertool are depicted in Figure 1. Compared to normal or normal-adjacent tissue, CA9 expression is increased in tumors, whereas CA12 is decreased, which were significant in all three datasets (Figure 1a). In addition, CA9 expression positively correlated with HIF1α expression in all seven datasets, although reaching statistical significance in only one dataset (Figure 1b). Correlation analysis of CA12 with HIF1α showed a more varying pattern among datasets yet with an overall positive correlation and with statistically significant correlations in two datasets. This suggests, that not only CA9-, but also CA12 expression is associated with hypoxia in colorectal tumors. Analyzing the relationship of both CAs directly revealed an overall positive correlation, reaching statistical significance in three datasets (Figure 1b). Thus, CA9 and CA12 seem to have no complementary roles in colorectal tumors but rather can also occur in a co-expressed manner.

To investigate CA9/CA12 expression depending on the molecular subtype, we employed the same CRC datasets and applied the CMScaller [59] to classify tumor samples according to the CMS classification system. This resulted in a characteristic pattern of expression, which was observed in all seven CRC datasets, exemplarily represented by two datasets in Figure 1c (see Figure S1 in Supplementary Materials for the other five datasets). CA9 was more expressed in CMS1/CMS3 tumors compared to CMS2/CMS4 tumors. CA12 showed the highest expression in CMS3 tumors, followed by CMS1 tumors. Thus, the transcriptomic data revealed pronounced co-expression of both CAs as a typical feature of CMS3- and CMS1 tumors. In addition, the characteristic relation between CA expression in tumors versus normal tissue was reproduced (Figure 1c).

### 2.2. Differential Expression of CAIX and CAXII in Preclinical Models Representing CMSs

**Figure 2:**
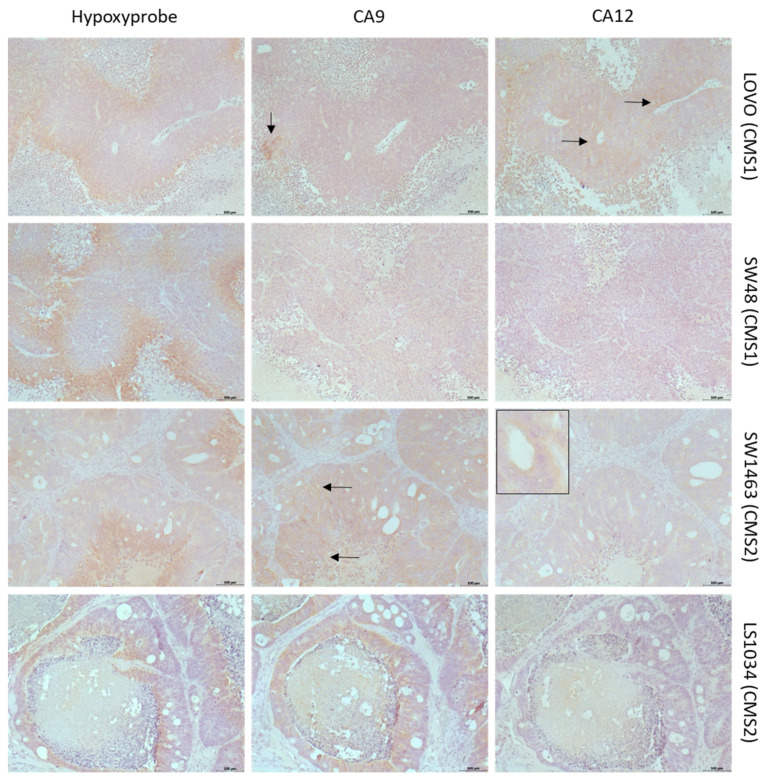

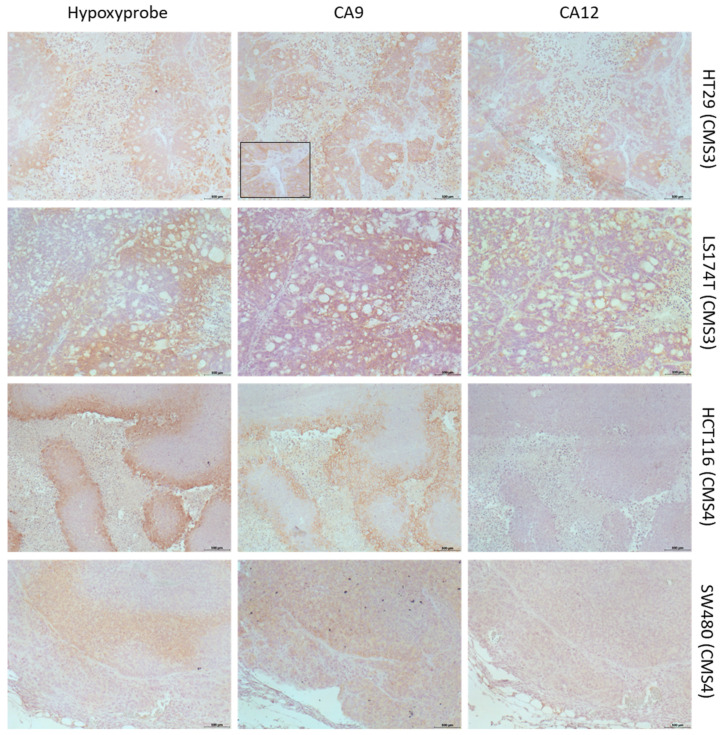
Immunohistochemical analyses of expression of CAIX and CAXII and presence of hypoxia (Hypoxyprobe, pimonidazole) in xenograft tumors representing different CMS. Staining was performed on direct following slides to capture the same tumor areas for analyses. Original pictures with higher quality, including selected images with higher magnification, can be found in Figure S2 (Supplementary Materials). LOVO (CMS1): CAIX expression is rare; positive cells are associated with hypoxic perinecrotic areas (arrow). CAXII is more frequently found and can occur outside of hypoxic tissue, even adjacent to vessels (arrows). SW48 (CMS1): CAIX is rare or absent despite hypoxia. CAXII is not found. SW1463 (CMS2): CAIX is nearly ubiquitously expressed and is found bound to hypoxic areas (arrow below) as well as independent of hypoxia (upper arrow). CAXII-positive cells can be found, but the signal is confined to the cytoplasmic compartment of colonic epithelial-like cells building ducts (inserted picture). LS1034 (CMS2): CAIX expression is frequent but rarer compared to SW1463 and more associated to hypoxia in comparison to SW1463 tumors. CAXII expression shows the same phenomenon as described for SW1463 (not shown). HT29 (CMS3): CAIX expression is nearly ubiquitous and is found in hypoxic and perinecrotic areas as well as in non-hypoxic areas, even adjacent to vessels (inserted picture). CAXII expression is rarer than CAIX expression but is still found widespread around the tumor mass and is particularly associated with morphological features of goblet cells. Large areas with co-expression of both CAs occur frequently. LS174T (CMS3): CAIX expression is widespread but rarer compared to HT29 tumors and is more associated to hypoxic areas. CAXII is frequently expressed and especially found in tumor areas showing morphological features of goblet cells. Areas with combined CAIX/CAXII expression are frequently found. HCT116 (CMS4): CAIX expression is strong, clearly associated with hypoxia and almost exclusively found in perinecrotic tissue. CAXII expression is missing completely. SW480 (CMS4): CAIX expression is exclusively found in hypoxic tissue. There is no CAXII expression. (Scale bar: 100 µm).

To investigate CAIX/XII expression in tumor tissues on a protein level, we employed our established preclinical CRC model comprising luciferase-expressing cell-line-derived nude mouse xenograft tumors and spheroids representing the four molecular subtypes according to the CMS classification system. Detailed characterization of the model, including generation of the luciferase-expressing variants of cell lines, transcriptomic data and CMS classification using the CMScaller [59], will be published in a separate paper. The results of CMS classification were in accordance with wild-type CRC cell lines, which were previously characterized in the study by Berg et al. [56]. Immunohistochemical analyses of both CAs and hypoxia (pimonidazole) showed a characteristic expression pattern among xenograft tumors of different CMSs. Figure 2 shows examples featuring each xenograft tumor type (see Figure S2 in Supplementary Materials for original pictures with higher quality, including additional images and selected images with higher magnification). In tumors derived from cell line LOVO representing CMS1, expression of CAIX was low in all examined tumors, and, if positive cells were found, they were associated with hypoxic areas, especially in perinecrotic tissue. CAXII was found more frequently; however, in contrast to CAIX, CAXII expression also occurred outside of hypoxic tissue, even adjacent to vessels. In SW48 tumors (CMS1), CAIX expression was found in even fewer cells than in LOVO tumors in three out of four tumors, while, in one of the four tumors, much stronger expression of CA9 was found. If found, CAIX expression was associated with hypoxic tissue. CAXII expression was not observed in any of the SW48 tumors. Overall, CMS1 tumors showed an undifferentiated phenotype and large portions of the tumors were hypoxic, yet expression of CAIX was rare. CAXII expression occurred in one CMS1 tumor but was not necessarily bound to hypoxic tissue. The rare CAIX protein expression was in contrast to the transcriptomic data obtained from tumor samples (Figure 1c), whereas a differential CAXII protein expression pattern among CMS1 tumors could be expected.

CMS2 tumors represented by xenografts from cell lines SW1463 and LS1034 frequently showed a colonic-epithelium-like differentiation pattern and were overall less hypoxic compared to the other tumor types, with hypoxic areas observed in some distance to the blood vessels. CAIX staining in SW1463 tumors revealed nearly ubiquitous expression as even tissue adjacent to vessels showed positive staining. Therefore, expression of CAIX was not necessarily bound to hypoxic areas but was also found in non-hypoxic tissue. CAXII-stained cells could be frequently found; however, the signal was confined to the cytoplasmic compartment and was typically observed in colonic epithelial cells’ building ducts. In LS1034 tumors, CAIX expression was frequent but rarer when compared to SW1463 as association of CAIX expression to hypoxia was stronger in LS1034 tumors. For CAXII, we observed the same phenomenon as described for SW1463. Together, CMS2 tumors showed very frequent expression of CAIX, with expression being more spread in SW1463 than in LS1034 tumors. Expression of CAIX can be associated with hypoxia, but both tumors also expressed CAIX in non-hypoxic tissue. Regarding CAXII, the clear cytoplasmic signal exclusively observed in distinct cell types in CMS2 tumors might be due to unspecific staining but could also be a specific feature of those cells. Otherwise, cytoplasmic expression of CAXII is probably less relevant regarding the therapeutic impact of CAIX/XII specific inhibitors. Strikingly, the overall strong CAIX protein expression in CMS2 xenograft tumors, in particular when compared to CMS1 tumors, was in clear contrast to the transcriptomic data of tumor samples (Figure 1c).

CMS3 tumors represented by xenografts from cell lines HT29 and LS174T showed pronounced differentiation propensity towards goblet-cell-like structures and were overall more hypoxic compared to CMS2 tumors. In HT29 tumors, CAIX expression was nearly ubiquitous. Most areas with expression of CAIX were also hypoxic, but expression of CAIX was also found in non-hypoxic areas. CAXII expression was rarer than CAIX expression but was still found widespread around the tumor mass. Expression of CAXII was almost exclusively found in hypoxic tissue, especially in areas showing morphological features of goblet- and secretory cells. Notably, in hypoxic areas, co-expression of both CAs could be observed. In LS174T tumors, CAIX expression was widespread but rarer compared to HT29 tumors. Most of the CAIX-positive cells were found in hypoxic areas, although expression was also found in non-hypoxic regions. In contrast to HT29, some areas, especially around blood vessels, showed no CAIX expression at all. CAXII expression was rarer than CAIX and almost exclusively found in tumor areas showing morphological features of goblet cells. Some of the cells in those regions showed unclear CAXII signals, similar to the phenomenon described for CMS2 tumors. Hypoxic areas were widespread but rarer than in HT29 tumors. CAIX/CAXII co-expression occurred in hypoxic areas in accordance with HT29 tumors. Thus, as a whole, CMS3 tumors showed the strongest expression of CAIX and CAXII compared to all other types and were particular characterized by occurrence of co-expression of both CAs in same areas and cells, a feature that could not be observed in any tumor outside of the CMS group. Interestingly, the strong combined protein expression of both CAs completely reflected the transcriptomic data (Figure 1c).

CMS4 tumors represented by xenograft tumors from cell lines HCT116 and SW480 showed an undifferentiated phenotype, and large portions of the tumors were hypoxic. In HCT116 tumors, CAIX expression was strong but almost exclusively found in perinecrotic tissue and, therefore, clearly associated with hypoxia. CAXII expression was not found in any of the examined tumors. The tumors were also non-hypoxic apart from the areas adjacent to necrosis. In SW480 tumors, CAIX expression was exclusively found in hypoxic tissue. In contrast to HCT116, some of the perinecrotic tissue showed no expression of CAIX. There was no CAXII expression in any of the examined tumors. Overall, CMS4 tumors showed strong and hypoxia-associated expression of CAIX on a protein level, although lack of CAIX in some hypoxic areas could also be observed. The partly strong expression of CAIX on the protein level in CMS4 tumors was not to be expected based on the transcriptome data, especially when compared to CMS1 tumors (Figure 1c).

To summarize the data obtained in the preclinical CRC tumor models, it can be stated that the models overall do reflect the specific characteristics of CRC regarding expression of CAs and hypoxia provided by the transcriptomic data (Figure 1b); i.e., CAIX and CAXII expression is largely positively correlated to hypoxia, even if not stringent, and both CAs can be occur in a co-expressed manner (Figure 1b). However, considering molecular-subtype-associated expression, there were clear differences between expression on a protein level in preclinical tumors and expression on a transcriptome level in the patient tumor samples. This could be explained by the known fact that transcriptomic expression levels must not necessarily be directly linked to proteomic expression levels and, as such, the high CAIX gene expression level in CMS1 tumors does not necessarily need to translate into high CAIX expression on a protein level. On the other hand, CA expression on transcriptome level does clearly vary among each group and show overlapping areas. Therefore, some of the used preclinical CMS-classified CRC tumor models probably do not reflect the most typical CA expression pattern of the respective group, assuming that each CMS group can be characterized by a specific CA protein expression pattern after all. Strikingly, CMS3 xenograft tumors completely reflected a CAIX/XII expression pattern, which was expected based on the transcriptomic data; hence, they are useful models representing CMS3 group tumors.

### 2.3. CAIX/CAXII Expression Is Associated with Response to SLC-0111 in Tumor Spheroids

Next, we questioned whether the CAIX/XII protein expression patterns observed in xenograft tumors are reproduced in their respective CMS-classified tumor spheroids in vitro. Tumor spheroids are useful in vitro models representing several important aspects of real tumor tissues, i.e., three-dimensional growth with structural organization and physiologically relevant cell–cell and cell–matrix interactions, establishment of tumor microenvironmental characteristics, such as nutrient gradients, hypoxia and acidosis, as well as drug resistance mechanisms [61,62]. Immunohistochemical analyses of tumor spheroid tissues largely reflected characteristics of xenograft tumors, except few variations. For example, in LOVO spheroids (CMS1), CAXII expression was hardly found, while CAIX was rare or absent in accordance with xenografts. In SW48 spheroids (CMS1), neither CA was observed, nearly reflecting xenografts. Thus, these spheroids represent models with rare or no CAIX/XII expression. Spheroids of SW1463 and LS1034 (CMS2) showed CAIX expression, and the phenomenon of specific differentiated cells with cytoplasmic CAXII signal could also be observed. Strong and combined expression of both CAs was the typical feature of HT29 and LS174T spheroids (CMS3), clearly reflecting characteristics of xenograft tumors. HCT116 spheroids (CMS4) showed typical strong CAIX expression bound to hypoxia and adjacent to the core necrotic area. In SW480 spheroids (CMS4), CAIX was ubiquitously expressed throughout the tissue; thus, the relation of CAIX expression and tumor mass was much higher compared to xenograft tumors.

Using these representative models, we tested the impact of specific CAIX/XII inhibitor SLC-0111 on tumor spheroid growth. To this end, we performed our established spheroid cytotoxicity assay, which is based on stable luciferase expression of tumor cells, enabling monitoring of spheroid growth and response to therapy. Calculated IC_50_ values derived from dose–response curves are summarized in Table 1. There was a differential response among models, reaching from completely insensitive (CMS1 spheroids) to clearly responding (CMS3 spheroids) when exposed to the reported peak plasma levels of SLC-0111 of around 20 µM [48]. Differential response was clearly associated with differential CA expression. Interestingly, the SW480 model (CSM4) was almost as sensitive as our CMS3 models. Together, the data obtained in spheroid models clearly suggest a CMS-related propensity to respond to CA inhibition, with no (CMS1), moderate (CMS2), clear (CMS3), and moderate to clear (CMS4) response. In addition, considering the ability of our preclinical CMS3 models to reflect transcriptomic pattern of CMS3 tumor samples, it can be assumed that patients with CMS3 tumors would most benefit from treatment with CAIX/XII-specific inhibitors such as SLC-0111. Conclusions with regard to the other CMS groups are not possible at this point. It remains to be investigated whether they can also be characterized by specific CA protein expression patterns. Nevertheless, based on the data obtained in these preclinical models, response to CA inhibitors can be assumed in tumors with substantial CAIX expression independent of the CMS.

### 2.4. Role of CAIX/CAXII Expression for Clonogenic Survival in CMS3 Modeled Cells

**Figure 3:**
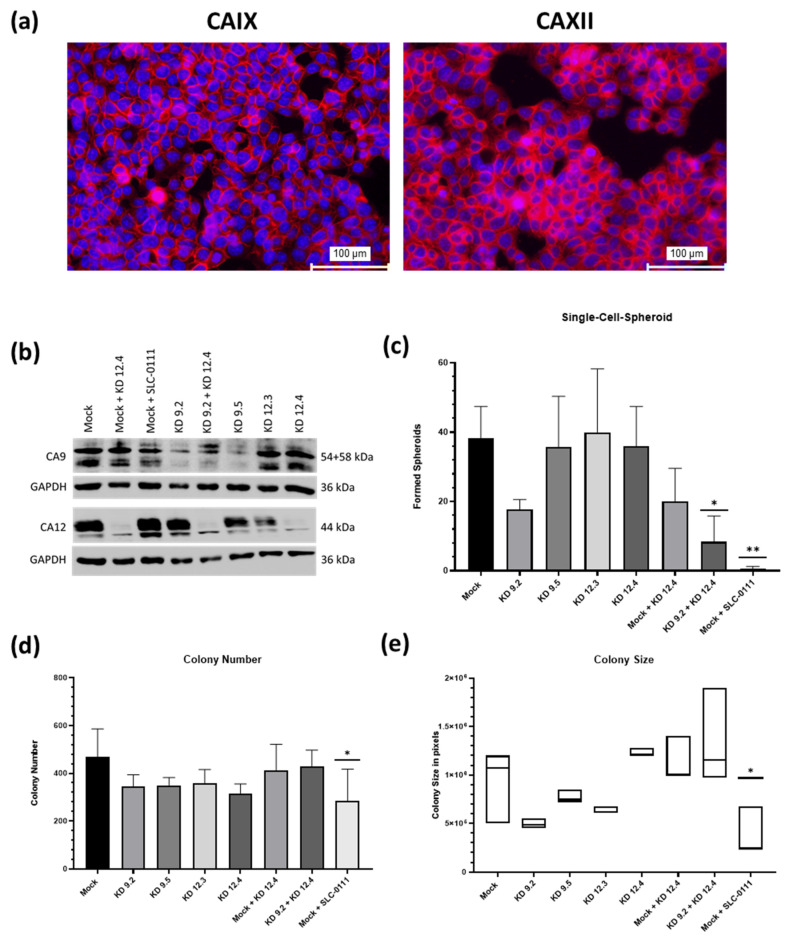
Impact of CAIX/CAXII expression on tumor biological functions of HT29 cells (**a**): immunocytochemistry of monolayer culture showing ubiquitous expression of both CAIX (left) and CAXII (right) (scale bar: 100 µm). (**b**): Western blot analyses of CAIX/CAXII expression in mock and knockdown cells. Two different shRNAs were used for each CA. (**c**): Spheroid forming from single-cell level was significantly reduced in cells with the combined CAIX/CAXII knockdown and mock cells treated with SLC-0111 (mean with SD, n ≥ 3). (**d**): Significant reduction in colony number in mock cells treated with SLC-0111 (mean with SD, n ≥ 3). (**e**): Significant reduction in colony size in mock cells treated with SLC-0111 (median with range, n ≥ 3). The clonogenicity assay and single-cell spheroid assay were repeated three or more times per condition in separate trials. (*: *p* ≤ 0.05, **: *p* ≤ 0.01).

The monolayer cultures of our models, analyzed by immunocytochemistry, largely reflected the specific CAXII expression pattern observed in xenograft tumors and spheroids as some LOVO cells and virtually all cells of HT29 and LS174T were positively stained. As expected, the CAIX expression pattern was different. In CMS1 and CMS4 cell lines, CAIX-positive cells were hardly found. HT29 (CMS3) and SW1463 (CMS2) showed strong and ubiquitous expression of CAIX, whereas it was far less in LS174T (CMS3) and LS1034 (CMS2). Notably, HT29 cells were characterized by virtually ubiquitous co-expression of both CAs (Figure 3a), reflecting the most typical feature of CMS3 tumors already on the monolayer level of cell culture. Therefore, it represents a sufficient model to study the importance of expression and activity of both CAs for different tumor functions.

To this end, we analyzed the impact of knockdown or inhibition of CAs on ability of cells to build colonies from single cells under adherent and non-adherent conditions, i.e., colony-forming in six-well plates and single-spheroid-forming in 96-well plates. After establishing stable knockdowns of each CA and of both combined, successful depletion was controlled by means of Western blot analysis of protein lysates prepared from monolayer- and spheroids culture (Figure 3b). Aside from the impact of knockdowns, treatment of HT29 mock cells with CAIX/XII inhibitor SLC-0111 slightly reduced CAIX level but showed no influence on CAXII levels. In the colony-forming assay, only small effects but no significant changes in colony numbers could be observed in any of the knockdown variants (Figure 3d). However, treatment with SLC-0111 resulted in significant decrease in colony number in mock cells (Figure 3d). Mock cells treated with SLC-0111 also showed a significant decrease in colony size, while none of the other knockdown variants showed significant reduction in colony size (Figure 3e). The amount of spheroids formed from single cells was significantly decreased in cells with combined CAIX/CAXII knockdown as well as in mock cells treated with SLC-0111 (Figure 3c). Thus, combined reduction in CAIX/CAXII expression affected clonogenic survival in part, whereas combined inhibition of activity of CAs led to effective reduction in cell survival in each condition.

### 2.5. Impact of CAIX/CAXII Expression on Chemotherapy in CMS3 Spheroids

Using the HT29 spheroid model, we investigated the impact of different knockdowns and inhibition of CAIX/CAXII by means of co-treatment with SLC-0111 on efficacy of chemotherapeutic drugs 5-fluorouracil (5-FU), irinotecan and oxaliplatin. There were no significant differences in IC_50_ between mock cells and knockdown variants, although a tendency of sensitization towards 5-FU and oxaliplatin could be concluded from slight shifting of knockdown curves, in particular of the combined knockdown. The rather small effects of knockdowns, already observed in the single-cell assays, could be explained by probably sufficient residual expression of CAIX and CAXII despite knockdown resulting in no substantial alteration of the microenvironment.

**Figure 4:**
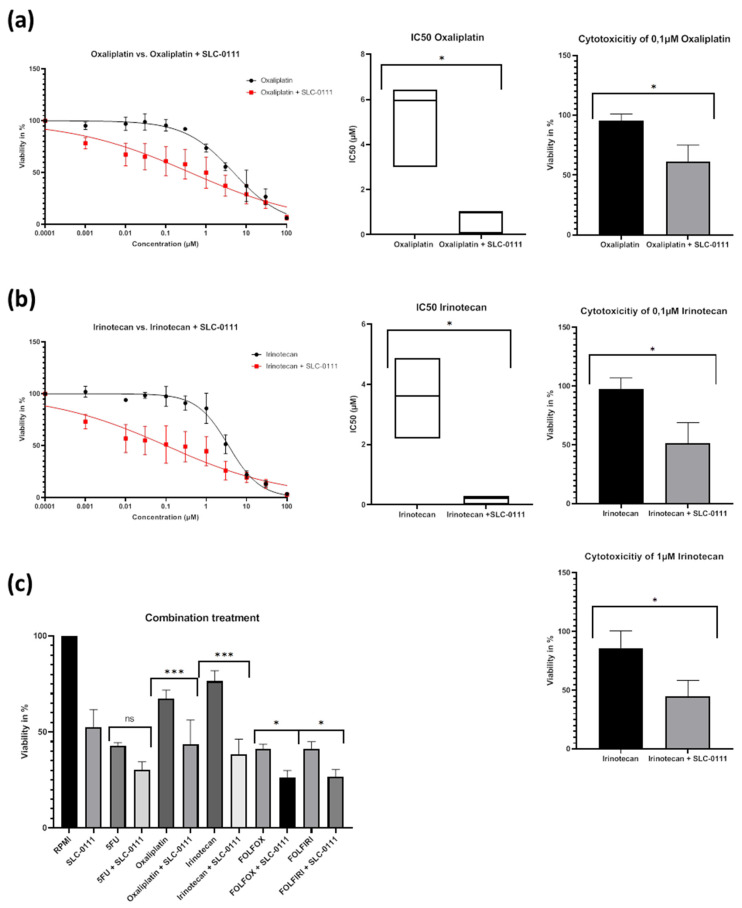
Impact of SLC-0111 co-treatment on chemotherapy in HT29 spheroids. (**a**): Co-treatment of oxaliplatin and SLC-0111 results in significant decrease in IC_50_ value (µM, median with range, n ≥ 3) and cell viability at oxaliplatin concentration of 0.1 µM (mean with SD, n ≥ 3). (**b**): Co-treatment of irinotecan and SLC-0111 results in significant decrease in IC_50_ value (µM, median with range, n ≥ 3) as well as cell viability at irinotecan concentrations of 0.1 µM and 1 µM, respectively (both mean with SD, n ≥ 3). (**c**): Additional treatment with 20 µM SLC-0111 results in significant decrease in cell viability in spheroids treated with 0.1 µM irinotecan and 0.1 µM oxaliplatin as well as spheroids treated with combinations of chemotherapeutic agents reflecting the FOLFOX (1 µM 5-FU; 0.1 µM Oxaliplatin) or FOLFIRI (1 µM 5-FU; 0.1 µM irinotecan) regimens (all mean with SD, n ≥ 3). (*: *p* ≤ 0.05, ***: *p* ≤ 0.001).

Co-treatment with SLC-0111 led to clear sensitizing effects towards chemotherapy (Figure 4). For both oxaliplatin and irinotecan, a significant reduction in IC_50_ was achieved by adding SLC-0111. Furthermore, analyses of selected and clinically relevant concentrations around the IC_50_ values revealed a significant reduction in spheroid viability at 0.1 µM of oxaliplatin and at 0.1 µM as well as 1 µM of irinotecan. Clear, but not significant, reduction in spheroid viability could also be observed at 1 µM of oxaliplatin. Together, this showed that additional SLC-0111 treatment is capable of increasing cytotoxicity, especially at low concentrations of the chemotherapeutic agents (Figure 4a,b). Sensitizing effects were also observed with regard to 5-FU, but the characteristic flattened course of curves precluded clear calculation of IC_50_ values and their comparison.

As standard protocols for chemotherapy of CRC consist of combination schemes (FOLFOX: 5FU + oxaliplatin; FOLFIRI: 5FU + irinotecan), we furthermore investigated impact of co-treatment with SLC-0111 on combination chemotherapy. For this purpose, we used defined drug concentrations and analyzed dual and triple combinations of drugs (Figure 4c). Co-treatment with SLC-0111 led to significant improvement in chemotherapeutic efficacy in the dual therapy scheme, except for 5-FU, which was in accordance with results obtained from calculations of dose–response curves. As expected, the triple therapy regimens were also clearly more effective. Notably, a significant sensitizing effect resulted from addition of SLC-0111. Together, this suggests that inhibition of CAIX/CAXII by co-treatment with specific inhibitors can improve efficacy of chemotherapy independent of the used therapy protocols.

## 3. Discussion

With the recent introduction of consensus molecular subgroups (CMS), the focus on developing treatment strategies for a more individual and specific therapy for each patient in addition to the already existing therapies is as urgent as ever before. Therefore, new therapeutic targets have to be found and already existing ones have to be reexamined for their usefulness for individual therapy. Two proteins belonging to the second of the aforementioned categories are carbonic anhydrase IX and carbonic anhydrase XII. It is well documented in the literature that cancer cells modify their tumor microenvironment as a result of their otherwise unsustainable growth by changing extracellular pH values, leading to several effects benefiting the tumor and hindering the anti-cancer therapy [2,5,9,10,14], e.g., inhibition of immune function [6], selection of more malignant tumor cells [7], degradation of the extracellular matrix [8], angiogenesis [9] and reduction in efficacy of chemotherapy [10]. Tumor-associated carbonic anhydrases CAIX and CAXII were discovered over two decades ago [19,21]. Their unique niche in the enzyme family of the carbonic anhydrases has long made them interesting targets for anti-cancer therapy as their expression in healthy tissue is by far not as widespread as the expression of other members of their enzyme family, e.g., carbonic anhydrase II or IV. Their importance in creation and retention of this unique microenvironment has already been reported on [14,15,16]. As such, our goal in this study was to examine the potential of CAIX/CAXII inhibition for treatment of CRC. In order to examine the expression patterns of CAIX/CAXII in the different CMS groups, we analyzed the expression of the CAs in transcriptome data of human tumor samples and standardized cell-line-derived preclinical models of the different CMS groups.

Analyzing the transcriptome data of both CA9 and CA12 in both healthy and cancerous human tissue, we observed that CA12 was downregulated in CRC compared to healthy tissue. This finding was consistent with previous literature [24,63]. The opposite was observed for CA9 as CA9-expression increased in cancer tissue when compared to healthy tissue [23,24]. It is important to note that expression of both CAs was highest in CMS3 compared to the other CMS groups. It is known that CMS3 tumors show an increase in gene expression of goblet cell marker genes and show the highest amount of goblet cells out of all CMS groups [64]. The goblet cells, along with other cells involved in secretion and water absorption, also make up a large part of the cells showing CA expression in healthy tissue of the large intestine [65]. Several authors have previously stated that expression of tumor-associated CAs was likely to be linked to the origin of the cancer cell itself, among other factors, which might also explain why expression of CAIX in both the models of CMS2 and CMS3 was not exclusively found in hypoxic tissue [65,66].

While the transcriptome data were consistent with positive correlation of hypoxia to CA expression, there were also significant differences in our findings between protein and transcriptome level, as mentioned above: While patient tumor samples classified into the CMS1 group showed the highest expression of CA9 and CA12 (together with CMS3) on a transcriptome level, neither our immunohistochemical analysis of spheroid and xenograft tumor tissue nor our analyses in the 2D models showed any abundance of CA expression close to that of the CMS3 group. Aside from CMS1, the transcriptome data for CMS2 tumors showed less expression of CA9, while the xenograft tumors showed high abundance of expression of CAIX. This indicates that there is a complex multifactorial mechanism behind regulation of CAIX and CAXII expression. Although HIF-1 plays the major role in regulation of expression of CAIX and CAXII, it is far from the only protein involved as other mechanisms, including MORC2 and non-coding RNA, were reported on recently [66,67,68,69,70]. Additionally, di Fiore et al. recently reported on the currently known posttranslational modification mechanisms of CAIX and CAXII and stated that these modifications partly have a yet unknown impact on the function of CAIX/CAXII, indicating that there may also be fluctuation in CAIX/CAXII efficacy present on a protein level [71].

To our knowledge, no work has been published yet in which expression of CAIX and CAXII in the different CMS groups of CRC was systematically analyzed by means of immunohistochemistry. As described above, the individual strength of expression of CAIX/CAXII varied from cell line to cell line, yet the pattern in which CAIX and CAXII were expressed was close to the same when comparing the two cell lines of each CMS group with each other. However, expression patterns greatly varied between the different CMS groups, thus suggesting that the distinct molecular features that lead to classification of CRC into different CMS groups also play a huge part in expression of carbonic anhydrase IX and XII. We further validated our findings of differentiated expression with the help of the 3D spheroid model, in which we were able to show that: (1) close to no differences in expression patterns exist between the spheroid and xenograft model and (2) the spheroid model itself as a basic 3D model already shows distinction from the results of our 2D models. As such, the spheroid model proved to be a sufficient 3D model to assess the importance of CAIX and CAXII for tumor function. It is important to note that this distinction is also due to the different conditions the cancer cells are exposed to as, e.g., there is an abundance of oxygen and nutrients in the 2D-models, which are not present in our 3D-models. Therefore, the 2D- and 3D-models are to be compared with caution.

As described above, the effect of sole SLC-0111 treatment on spheroid viability differed between the different cell models of CRC depending on the CMS group. While SLC-0111 treatment of SW48 and LOVO of CMS1 had no effect on spheroid viability, HT29 and LS174T spheroids of CMS3 showed clear response to treatment with SLC-0111. The cytotoxic effects on spheroid models of CMS2 and CMS4 were stronger than in CMS1 but weaker than in CMS3. These findings led us to the conclusion that efficacy of SLC-0111 is very much dependent on the expression status of CAIX and CAXII in the tumor mass as LOVO and SW48 cells showed little to no expression of neither CAIX nor CAXII in both spheroids and xenograft tumors. The findings in CMS1 also proved that SLC-0111 itself probably has no effect on cell viability of cancer cells outside of inhibition of CAIX and CAXII. The IC_50_ value for these two models was outside the used concentrations in our assay, which already far exceeded the dosage previously tested as safe for humans in a phase 1-study conducted on SLC-0111 [48]. In sharp contrast, the HT29 and LS174T models, which we identified as the tumor models with the strongest expression of CAIX and CAXII, showed the strongest reduction in spheroid viability and, therefore, clear response to treatment with SLC-0111. This further proved that SLC-0111 efficacy is closely linked to the CAIX/XII expression status of the tumor.

These findings suggest that SLC-0111 could be a potent weapon for treatment of CRC, but its usage is limited to tumors with high expression of CAIX and CAXII. Therefore, a histopathological examination of the patient’s tumor or other procedures, such as PET imaging [72,73], are necessary in order to deduce if therapy with a CAIX/XII inhibitor such as SLC-0111 will be beneficial for the patient due to high expression of CAIX and/or CAXII. These findings may explain the results of the CRC patients in the phase 1 study for SLC-0111, in which only one of the enrolled CRC patients showed a period of stable disease upon treatment with SLC-0111 [48], which could be due to a tumor with high expression of CAIX and CAXII, most probably a CMS3 tumor. As such, our preclinical data demonstrate the benefit of SLC-0111 treatment but also suggest that far from all patients would benefit from additional therapy with such a CA inhibitor. The findings also revealed very differentiated expression of CAIX and CAXII in the different CMS groups, further confirming that assessing the CMS groups in CRC patients is very important to provide the patient with the most beneficial additional anti-tumor treatment, e.g., in the form of SLC-0111 if the CAIX/CAXII expression in the tumor mass is high, as in a CMS3 tumor. Interestingly, the described “metabolic dysregulation” signature of CMS3 tumors [49] could explain the importance of CAIX/CAXII for these tumor types, resulting in pronounced sensitivity towards inhibition of both CAs.

Aside from the aforementioned 3D models, we also used immunocytochemistry in order to examine the expression status of CAIX and CAXII in the different CMS groups on a 2D monolayer level. In these models, HT29 was the only cell line to show strong staining for both CAIX and CAXII. Further, we observed several differences between our 2D- and 3D models. The most striking difference was observed for the LS174T cell line, which showed only little expression of CAIX compared to HT29 in our monolayer models but showed widespread expression in xenograft tumors. CAIX expression in the LS174T cell line was mostly found in hypoxic tissue, and the monolayer model does not provide a severely hypoxic environment; however, this alone cannot be the reason for the lack of CAIX expression as HT29 cells showed a high abundance of CAIX in the 2D models although also exhibiting a strong association between CAIX and hypoxic tissue. In addition, both cell lines of CMS 4 showed close to no CAIX expression in our 2D models, although expression was present in a moderate and sometimes even high abundance in both the xenograft tumors and spheroids. We observed the opposite trend in the LOVO cell line as we observed less expression of CAXII in 3D models but more expression of CAXII in the monolayer culture. Therefore, we assume that expression of CAIX/CAXII is not only influenced by oxygenation of the tissue but also relies on other factors, such as tumor architecture, cell differentiation and cell–cell-interaction. These examples show that a 2D approach of analyzing expression of CAIX and CAXII does not necessarily reflect the real expression status in a tumor and that 3D models, therefore, appear to be more realistic models of CA expression in the tumor mass.

As described above, HT29 cells were found to have the highest expression of CAIX and CAXII in both our 2D- and 3D models; thus, they represent the most typical feature of CMS3 tumors in vitro already. Therefore, we decided to use HT29 cells as our model to examine the importance of CAIX and CAXII for tumor function. The effects of the knockdowns of CAIX and CAXII were not significant in any of our trials. It is important to note, however, that these knockdowns did have a negative effect on the tumor cells in our single-cell spheroid assay and our clonogenicity assay, respectively. Therefore, we concluded that reducing the expression of these genes by a certain amount had a negative impact on tumor function, but not a significant one. Treating HT29 cells with CA inhibitor SLC-0111, however, showed a significant impact on a variety of tumor functions. The ability of HT29 cells regarding clonogenic survival on a monolayer level was impaired severely as both number and size of the colonies were reduced by over 90%. Similar findings were also reported by Parks et al., who showed that disruption of CAIX severely reduced clonogenic proliferation in LS174T cells [13]. SLC-0111 treatment also resulted in a significant reduction in spheroid forming in our single-cell spheroid assay. The assay was designed in order to simulate metastasis at a single-cell stage, shortly after a cell implanted itself into the new tissue. It is important to note that HT29 cells, in which both CAs were knocked down, also showed significant reduction in ability of spheroid forming. This indicates that CIX and CAXII may be especially important for cancer cells in the very early stages of cancer development, in which cell–cell-interaction and surrounding soft tissue are limited or not yet present and decreased protein expression or protein inhibition of CAIX/CAXII hinders the cancer cell’s survival in that early stage. CRC is a special type of cancer regarding metastasis as the latency between the diagnosis of the primary tumor and the appearance of metastasis is relatively small [74]. Therefore, it is very important to further understand the mechanisms behind the metastatic process and to continue to find marker and target proteins for metastasis in order to prevent the cancer from spreading out of its organ of origin [75].

One of the largest challenges in the medicamentous approach of cancer treatment is the resistance of tumors towards chemotherapeutic agents. Cancer cells are capable of several mechanisms that can reduce chemotherapeutic efficacy. Alteration in extracellular pH to more acidic values is commonly observed in chemotherapy-resistant tumors and leads to reduced intracellular accumulation of the chemotherapeutic agent due to a shift in the concentration gradient between the acid and base form of the drug [5,76]. The literature has already reported on pH-dependent uptake of irinotecan, a drug commonly used in medicamentous therapy of CRC [77]. Tumor-associated carbonic anhydrase IX and XII play a major role in establishing this pH disbalance by forming a functional complex, a so called “Metabolon”, which facilitates extrusion of acidic metabolites created in the cellular metabolism to the extracellular space [5,15,16,78,79]. The literature has already shown that disruption of the CAIX pathway leads to a reversal of the described pH-altering effect [80]. P-glycoprotein-mediated extrusion of chemotherapeutic agents has long been established to be a mechanism by which tumor cells are able to avoid otherwise lethal concentrations of chemotherapeutic therapy. Interestingly, CAXII has been reported to be associated with p-glycoprotein (Pgp) in several tumors, and it was shown that chemotherapeutic efficacy can be increased by means of CAXII inhibition [30,81,82,83]. Although HT29 cells show high expression of CAXII, expression of Pgp in this cell line is low [81]. As such, this manner of chemotherapeutic resistance plays a minor role at best in our HT29 model but may play a major role in other tumors expressing CAXII, on which the literature has already reported before [84,85]. Aside from the aforementioned mechanisms, there are many more ways described in the literature by which CRC cells may be able to become resistant against basic chemotherapeutic drugs 5-FU, irinotecan and oxaliplatin [86]. Our findings in the co-treatment trials suggest that a combined treatment of SLC-0111 plus the conventional chemotherapeutic agents could be beneficial in treating CRC if the tumor presents itself with a high expression status of the tumor-associated carbonic anhydrases. The increase in cytotoxicity was especially strong in the HT29 cells treated with irinotecan and oxaliplatin as, without SLC-0111, both irinotecan and oxaliplatin only showed little effect on cell viability in concentrations that might be administered to humans. These findings proved that the combination of the normally used chemotherapeutic agents and SLC-0111 is superior to chemotherapy alone in our model. Additive effects were observed for 5-FU. Therefore, treatment with the commonly used FOLFOX or FOLFIRI regimens in combination with SLC-0111 is entirely viable and showed a significant decrease in spheroid viability in our combination treatment trials and might, therefore, be an option for chemotherapeutic treatment of CRC if expression of CAIX and CAXII is high in the patient’s tumor.

## 4. Materials and Methods

### 4.1. Transcriptomic Analyses of CRC Samples

In order to assess transcriptomic data of CA9 and CA12, as well as HIF1α in human tumor samples we used the platform Cancertool [60]. Cancertool is an online bioinformatic platform performing expression-, correlation- and gene-enrichment analyses based on seven different colorectal cancer transcriptomic datasets: Colonomics, GSE44076; Jorissen et al., GSE14333; Kemper et al., GSE33113; Laibe et al., GSE37892; Marisa et al., GSE39582; Roepman et al., GSE42284; TCGA, cBioPortal. Detailed information about the data sources and methods used by Cancertool can be found on the website: http://genomics.cicbiogune.es/CANCERTOOL/index.html (accessed on 13 March 2023). In addition, using these datasets, the patient’s tumor data were classified and separated into the CMS groups using the CMScaller [59], in order to gain differentiated analysis on the expression patterns of both CA9 and CA12 in the different CMS groups. The classification was performed accordingly to the instructions provided on https://github.com/peterawe/CMScaller (accessed on 13 March 2023). Boxplots were generated with BoxPlotR (http://shiny.chemgrid.org/boxplotr/, (accessed on 13 March 2023)).

### 4.2. Preclinical Tumor Models

The preclinical CRC model used in this study was established in the context of a separate study and comprises luciferase-expressing variants of the cell lines SW48, LOVO, SW1463, LS1034, HT29, LS174T, HCT116 and SW480, derived nude mouse xenograft tumors and spheroids representing the four molecular subtypes according to the CMS classification system. Detailed characterization of the model including generation of the luciferase-expressing variants of cell lines, transcriptomic data and CMS classification using the CMScaller [59] will be published in a separate paper. CMS classification was in accordance to the wild-type CRC cell lines, which were previously characterized by Berg et al. [56]. The new generated luciferase-expressing cell lines were re-authenticated at the DSMZ-German Collection of Microorganisms and Cell Cultures GmbH (Braunschweig, Germany) in 2020/2021.

The monolayer cells were cultivated in RPMI1640 medium (Sigma-Aldrich, St. Louis, MO, USA), to which 10% fetal bovine serum (Biowest, Nuaillé, France) and 1% penicillin-streptomycin (Sigma-Aldrich) was added. For generation of single tumor spheroids, tumor cells resuspended in culture medium were seeded into 96-well plates, which were coated before with 0.7% agarose (SeaKem^®^ GTG™ Agarose, Lonza, Basel, Switzerland). Cell lines HT29-Luc, DLD-1-Luc, LS174T-Luc, LS1034-Luc and SW1463-Luc were able to form compact spheroids within 2 days. For the cell lines LOVO -Luc, SW48-Luc, COLO205-Luc, HCT116-Luc and SW480-Luc, culture medium was supplemented with 10 µg/mL collagen I (Ibidi GmbH, Gräfelfing, Germany) to support spheroid formation. Completely compacted spheroids were formed within 7 days.

Stable knockdowns of CA9 and CA12 in HT29 cells were generated by lentiviral transduction using ready-to-use prepared virus particles from Sigma-Aldrich. The single knockdowns KD 9.2”, ”KD 9.5”, ”KD 12.3”, ”KD 12.4”were obtained with puromycin selection. As a control for the puromycin-based vectors, HT29 cells were transduced with an empty puromycin vector (“Mock”). The vectors of the more potent knockdown of CA9 (KD 9.2) and CA12 (KD 12.4) were chosen to be combined to double to generate a combined CA9/CA12 knockdown. While the aforementioned KD 9.2 vector was used, for the KD 12.4 knockdown a GFP labelled vector was used. FACS sorting was carried out at the local facility (Core facility, Center for Basic Medical Research (ZMG) of the Medical Faculty, Martin Luther University). As a control for the GFP-generated double-knockdown, HT29 mock cells were also transduced with the KD 12.4/GFP-vector (“Mock + KD 12.4”). Therefore, in both the “KD9.2 + KD12.4”, as well as the “Mock + KD12.4” samples, CA12 was knocked down using the same GFP-vector. For the Mock + SLC-0111 group, the aforementioned, puromycin-selected “Mock” cells were used.

### 4.3. Immunohistochemistry of Xenograft Tumors and Spheroids

The xenograft tumors samples used in this study for immunohistochemical analyses were prepared in the context of a separate study during the generation of our preclinical CRC model (see above). In order to be able to analyze intratumoral hypoxia, the mice were treated with Hypoxyprobe™ (Hypoxyprobe, Inc., Burlington, MA, USA) 90 min prior to tumor extraction. After extraction, the tumors/organs were fixated in formalin, before they were processed using the Microm STP120 and then embedded in paraffin using the Microm EC350-1 (both Thermo Fischer Scientific, Waltham, MA, USA). For immunohistochemical analyses, the tumors were cut into 4 µm wide samples. Following deparaffinization and rehydration, the samples were first subjected to peroxidase-blocking and then to protein-blocking. The used reagents were, if not stated otherwise, obtained from Dako (Agilent Technologies, Santa Clara, CA, USA). For the latter a 3% BSA (Carl Roth, Karlsruhe, Germany) in PBS solution was used. The samples were treated with anti-CAIX, anti-CAXII (AB184006 1:1000 and AB195233 1:100; both Abcam, Cambridge, UK) or anti-PAb2627AP (1:200, Hypoxyprobe, Inc.) primary antibodies, diluted in a 1% BSA in PBS solution, for three hours. They were then treated with biotinylated secondary antibodies (SC-2491, diluted 1:800 in PBS; Santa Cruz Biotechnology, Dallas, TX, USA) for one hour. After treatment with a Streptavidin-HRP-conjugate for 30 min, the samples were exposed to the chromogen 3,3′-diaminobenzidine (DAB) for five minutes, before all samples were stained with hematoxylin for one minute and then dehydrated. Images of the samples were taken on an Axiolab microscope equipped with Axiocam 503 color (Zeiss Group, Oberkochen, Germany).

### 4.4. Immunocytochemistry on Monolayer Culture

The used cells were seeded onto one well of a chamber slide (Nunc™ Lab-Tek™ II Chamber Slide™ System, Thermo Fischer Scientific). After 24 h of incubation the medium was removed and the plates were rinsed twice with PBS for two minutes each. The cells were then fixated using methanol (Sigma-Aldrich) for 15 min. The methanol was then washed away using PBS three times for 10 min, before the cells were treated with the same 3% BSA in PBS to achieve protein blocking. After that the cells were incubated with primary antibody (CAIX, AB184006 1:000 and CAXII, AB195233 1:175; both Abcam) diluted in a 1% BSA in PBS solution for two hours at room temperature. Following the incubation with the primary antibody, the plates were washed with a 1% BSA in PBS trice for 10 min each to remove excess antibodies. In the following step the plates were incubated with the secondary antibody (A-11036, 1:800, Thermo Fischer Scientific) diluted in PBS at room temperature in the dark for one hour. Excess antibody was then removed by washing the plates with PBS trice for 10 min each. During the washing steps, the plates were kept in the dark. In the next step the cells were stained with 4′,6-diamidino-2-phenylindole (DAPI, Sigma-Aldrich, stock solution 1mg/mL), which was diluted 1:1000 in PBS before, for three minutes. Following the staining process, the cells were washed twice with PBS for 5 min each in the dark. The plates were coated with mounting medium (Fluoromount-G™ Mounting Medium, Thermo Fischer Scientific) and then covered with a coverslip. Images of the samples were taken using the BioRevo BZ-9000 (Keyence Deutschland GmbH, Neu-Isenburg, Germany).

### 4.5. Protein isolation/Western Blot Analysis

Protein lysates of monolayer cells and spheroids were prepared using RIPA buffer. The RIPA buffer further contained 100mM phenylmethylsulfonyl fluoride, 10mM dithiothreitol (Bio-Rad Laboratories, Hercules, CA, USA), Protease-Inhibitor-Cocktail and Benzonase (both Sigma-Aldrich). After incubation on ice for 45 min, the lysates were then centrifuged at 15,000× *g* for 15 min at 4 °C. The protein concentration of the probes were then measured using the Bio Photometer D30 (Eppendorf SE, Hamburg, Germany) via Bradford method. The used Bradford reagent was obtained from Bio-Rad. The probes were adjusted to get equal concentrations.

The used Western blot equipment was, if not stated otherwise, obtained from Bio-Rad. Protein samples were separated using SDS-PAGE and subsequently transferred on a nitrocellulose blotting membrane for overnight blotting. Blocking of the membranes was accomplished using a 5% powdered milk (Carl Roth) in phosphate buffered saline (PBS) with 0.1% Tween (Sigma-Aldrich) solution, which was also used for dilution of the primary antibodies. The membranes were then treated with either anti-CAIX (M75 1:2000 (Bioscience Slovakia, Bratislava, Slovak Republic), anti-CAXII (AB195233 1:5000, Abcam) or anti-GAPDH (14C10, 1:5000, Cell Signaling Technology, Danvers, MA, USA) primary antibodies for two hours, before treatment with a horseradish-peroxidase (HRP) conjugated secondary antibody (SC-2357 or SC-516102; Santa Cruz Biotechnology) took place for one hour. The secondary antibodies were diluted 1:5000 in a 0.1% Tween in PBS solution, the same solution, which was also used to wash the membranes between and after antibody exposure. After treatment with a chemiluminescence detection reagent (ECL™ Western Blotting Detection Reagent, Sigma-Aldrich), images of the membranes were taken using the ImageQuant LAS 4000 (Cytiva Europe GmbH, Freiburg, Germany).

### 4.6. Clonogenicity Assay

Cell suspensions were first diluted to 500 cells in 5 mL of RPMI medium. The cells were then seeded onto six-well-plates. Ten days after seeding, the medium was removed and the plates were rinsed with PBS. The colonies were then fixated for 10 min using ethanol and afterwards stained with a 1% crystal violet (Sigma-Aldrich) in PBS solution for 10 min as well. The number and size of the colonies were analyzed using ImageJ (8-bit image; subtract background: 100 pixel, threshold: 212, analyze 3 particles: 40-3000, display results, summarize, add to manager, exclude on edges).

### 4.7. Single-Cell Spheroid Assay

Cell suspension were diluted to 100 cells in 20 mL of RPMI-medium and then seeded onto 96-well-plates (200 µL per well), which were previously coated with an 0.7% agarose (SeaKem GTG Agarose, Lonza Group, Basel, Switzerland) in PBS solution, in order to inhibit adhesion and therefore guarantee spheroid forming starting at a single-cell level. The formed spheroids were counted after 15 days.

### 4.8. Spheroid Cytotoxicity Assay

The cells were seeded onto a 0.7% agarose-coated, 96-well-plates and were incubated for two or seven days, depending on the model, to allow the undisturbed forming of compact spheroids, before treatment with chemotherapeutic agents/SLC-0111 was carried out. For the SLC-0111 response trial, the different spheroid models were exposed to the carbonic anhydrase inhibitor SLC-0111 (SLC-0111, Hycultec GmbH, Beutelsbach, Germany) at increasing concentrations (0.01, 0.03, 0.1, 0.3, 1.0, 3.0, 10.0, 30.0, 100.0 µM). For the co-treatment trials, spheroids were treated with SLC-0111 and one chemotherapeutic agent. Both drugs were diluted in 50 µL RPMI, and, while the chemotherapeutic agent was used in increasingly high concentrations (0.01, 0.03, 0.1, 0.3, 1.0, 3.0, 10.0, 30.0, 100.0 µM for oxaliplatin and irinotecan; 0.1, 0.3, 1.0, 3.0, 10.0, 30.0, 100.0, 300.0, 1000.0 µM for 5-fluorouracil (5-FU)), SLC-0111 was used in a concentration of 20 µM for all wells. Spheroids only treated with SLC-0111 served as a control to examine the sole efficacy of the CA inhibitor. The control group was treated with RPMI, in which DMSO was diluted, so that it could serve as a negative control for the effect of DMSO on cell viability. For the combination treatment with single doses, chemotherapeutic agents were used in a concentration of 1 µM for oxaliplatin and irinotecan or 10 µM for 5-FU. Spheroids were either treated with a monotherapy of one chemotherapeutic agent or a dual-therapy consisting of both agents (FOLFOX/FOLFIRI regiment). Each of the treatments served as an individual control group for the spheroids treated with the monotherapy/dual-therapy plus 20 µM of SLC-0111.

To measure and quantify the viability of the treated spheroids, 20 µL of D-luciferin (PerkinElmer, Waltham, MA, USA), diluted 1:1000 in RPMI-medium, was added to all wells 7 days after start of treatments. After 15 min of incubation in the dark, measurements were carried out on a Microplate Reader Tecan Spark (Tecan Group, Männedorf, Schwitzerland) using luminescence measurement with an exposure time of 1000 ms/well. The mean value of the eight values of each column was calculated and all values are given as % of untreated control. At least 3 independent experiments were performed and summarized as mean values with standard deviation.

### 4.9. Statistical Analysis

Statistical analysis was carried out using GraphPad Prism 8. The used *p*-value format for all analyses was NEJM. For the clonogenicity assay, the absolute values of colony numbers and colony size were examined for statistical significance by means of ordinary one-way ANOVA (assumed Gaussian distribution; assumed equal SDs) using the mock group as control for follow-up testing for statistical significance. Ordinary one-way ANOVA (assumed Gaussian distribution; assumed equal SDs) was also used for the single-cell spheroid assay, comparing the absolute values of each treatment to the control group mock. For the SLC-0111 response trial, an extrapolated curve was calculated via equation: [inhibitor] vs. response - variable slope to calculate the different IC_50_ values of each trial. The mean values and the SD of all measured IC_50_ values were then calculated for each spheroid model. For the co-treatment trials, IC_50_ values in both the control and the inhibitor group were calculated via equation: [inhibitor] vs. normalized response - variable slope. The IC_50_ values of both groups (Gaussian distribution assumed) were then compared using a Welch test or an unpaired *t*-test, depending if the variances were significantly different or not. Additionally, the relative viability of the spheroids in the control and inhibitor groups were compared at two different chemotherapeutic concentrations. The concentration was chosen closest to the peak plasma concentration of the respective agent in vivo (10 µM 5-FU; 1 µM irinotecan/oxaliplatin) and the second concentration was chosen at 10% of the first concentration (1 µM 5-FU; 0.1 µM irinotecan/oxaliplatin). The relative spheroid viability was compared using a Welch test or an unpaired *t*-test, as described above. For the combination treatment, the control groups and their respective inhibitor groups were compared to each other using traditional one-way ANOVA (assumed Gaussian distribution; assumed equal SDs).

## 5. Conclusions

This section is not mandatory but can be added to the manuscript if the discussion is unusually long or complex.

## Figures and Tables

**Table 1:** Response of tumor spheroids to SLC-0111. IC_50_ values (µM) ± SD (n ≥ 3).

LOVO	SW48	SW1463	LS1034	HT29	LS174T	HCT116	SW480
>100	98 ± 12	48 ± 12	56 ± 18	21 ± 4	19 ± 4	57 ± 7	23 ± 4
CMS1	CMS1	CMS2	CMS2	CMS3	CMS3	CMS4	CMS4

## Data Availability

The datasets used and/or analyzed in the current study are available from the corresponding author upon reasonable request.

## Supplementary Materials

The following supporting information can be downloaded at: https://www.mdpi.com/article/10.3390/ijms24065797/s1.
