# Supplementary figures and images for: Differential CMS-Related Expression of Cell Surface Carbonic Anhydrases IX and XII in Colorectal Cancer Models—Implications for Therapy
Source: Int J Mol Sci. 2023 Mar 18;24(6):5797. doi: 10.3390/ijms24065797 (PMC10056265; doi:10.3390/ijms24065797)

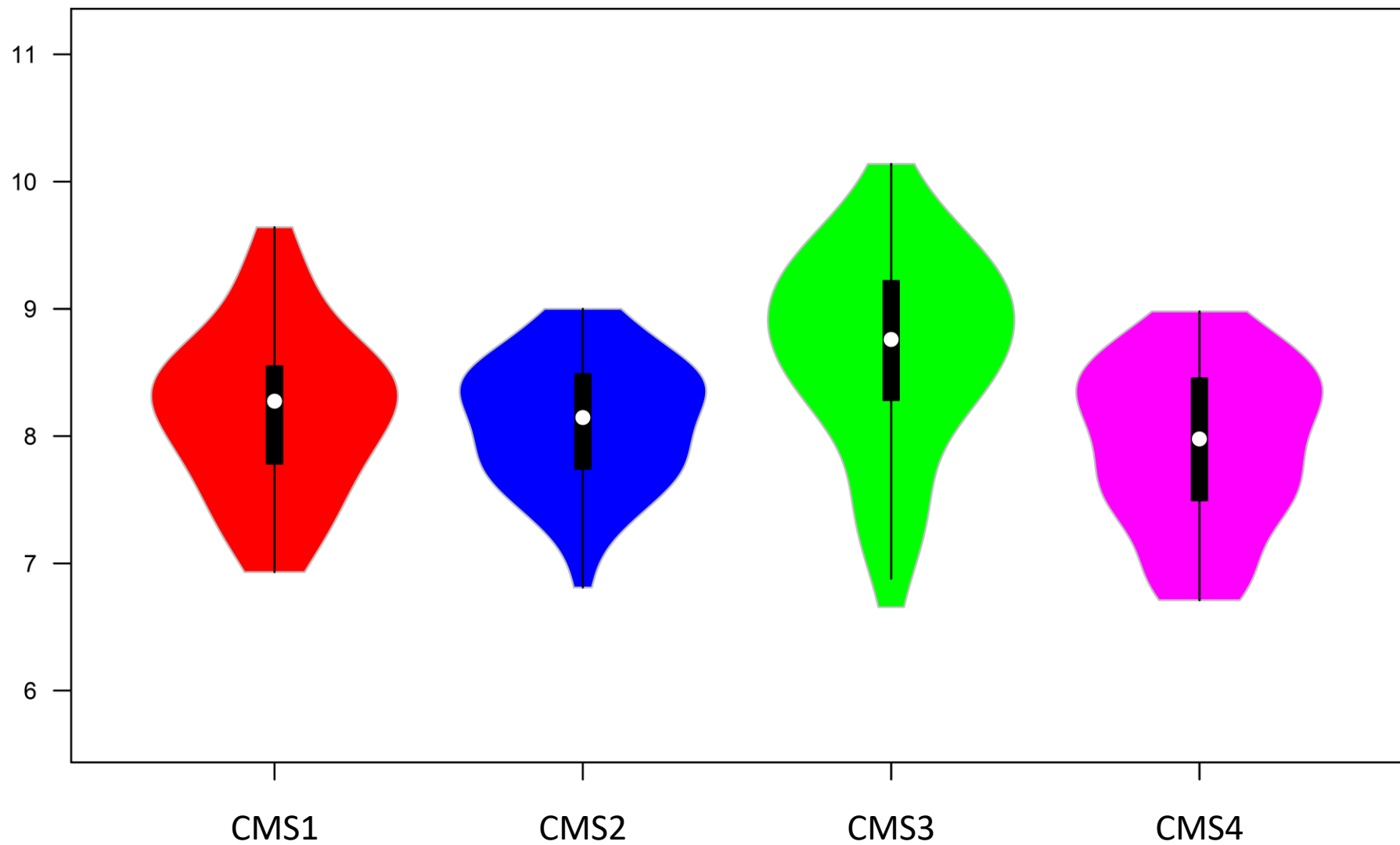

Supplement: Supplementary file 1 [file ijms-24-05797-s001.zip › Figure S1_CMS related CA9-CA12 expression in CRC samples/CA12_Jorissen_GSE14333.pdf]

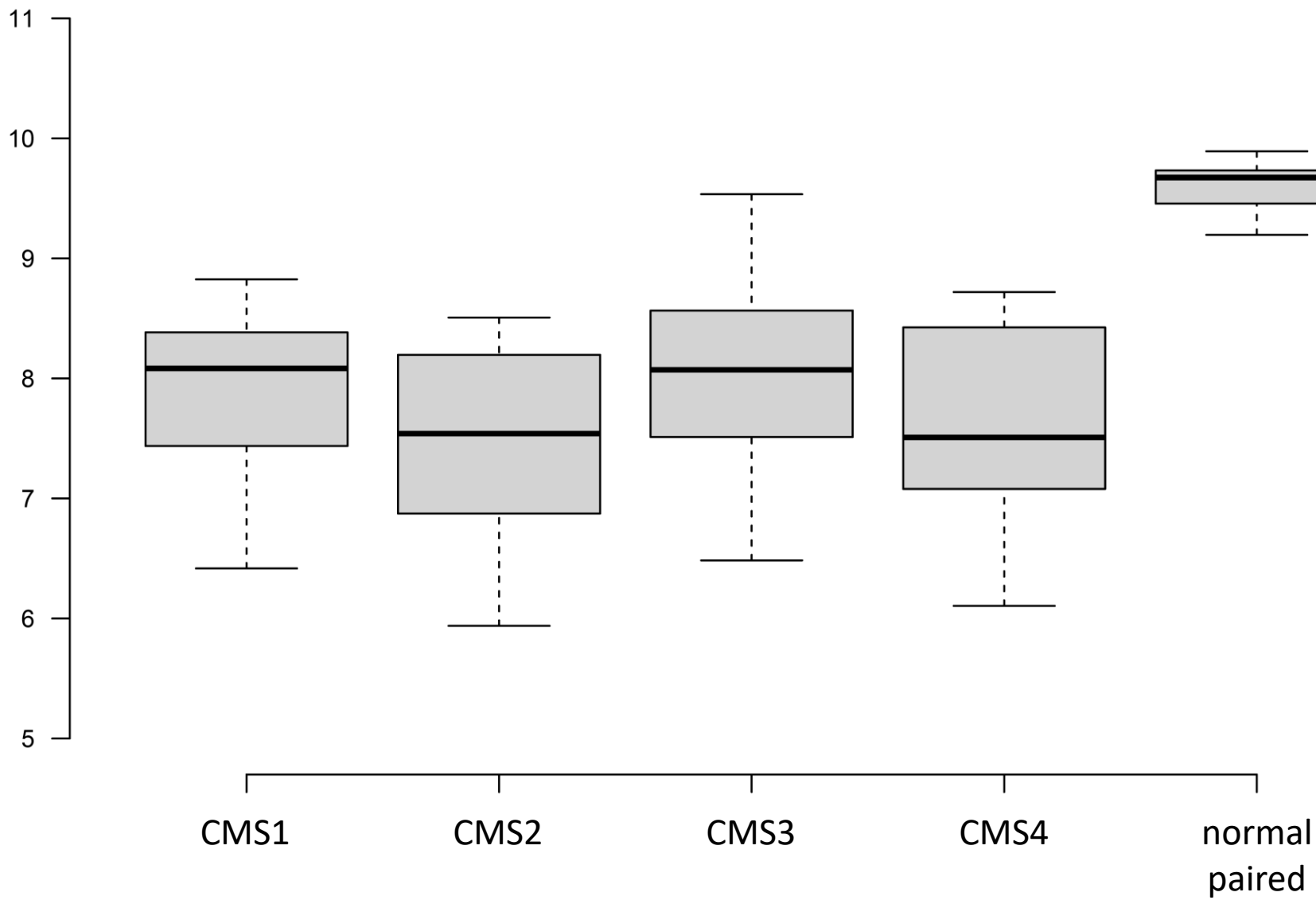

Supplement: Supplementary file 1 [file ijms-24-05797-s001.zip › Figure S1_CMS related CA9-CA12 expression in CRC samples/CA12_Kemper_GSE33113.pdf]

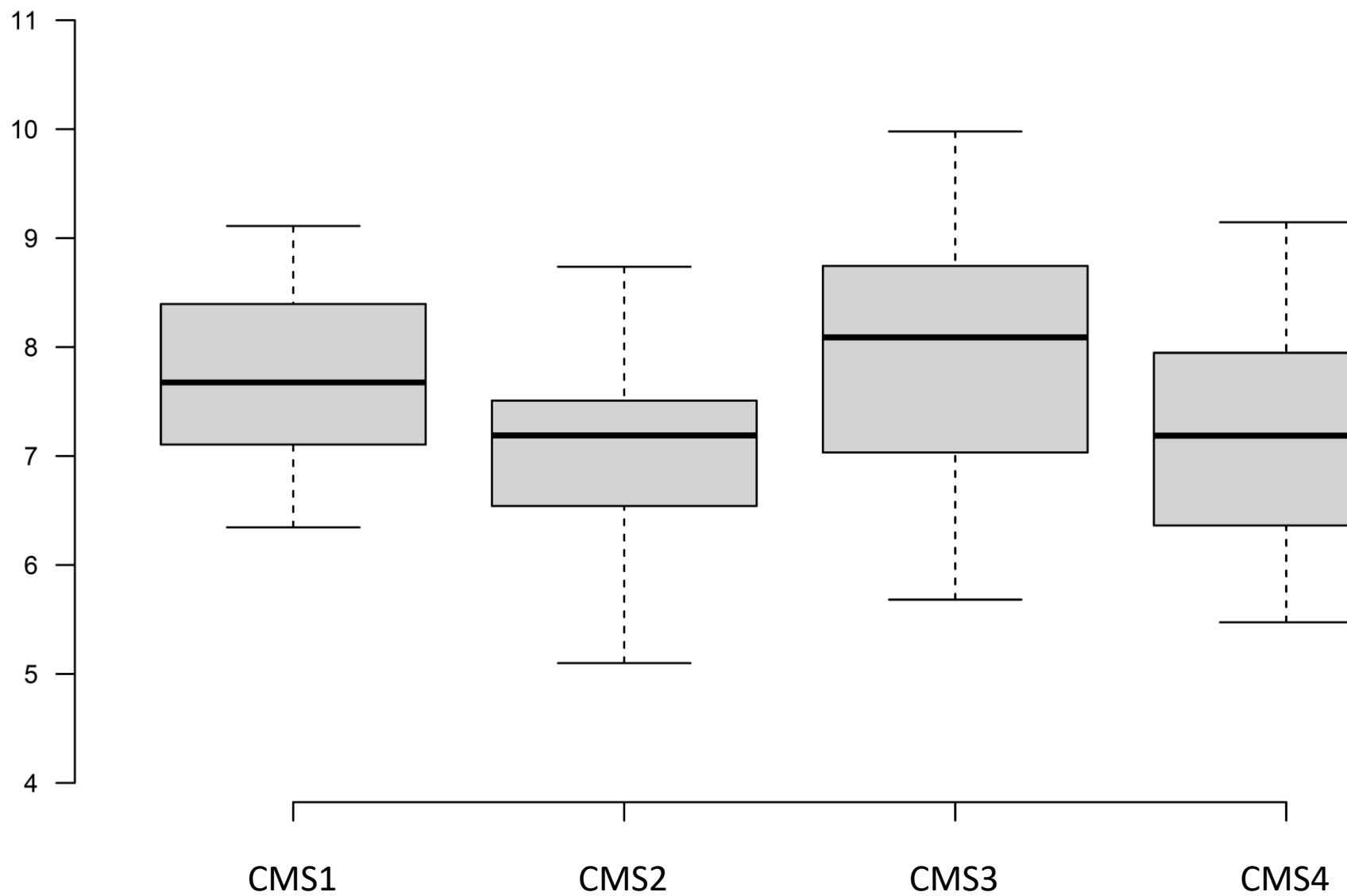

Supplement: Supplementary file 1 [file ijms-24-05797-s001.zip › Figure S1_CMS related CA9-CA12 expression in CRC samples/CA12_Laibe_GSE37892.pdf]

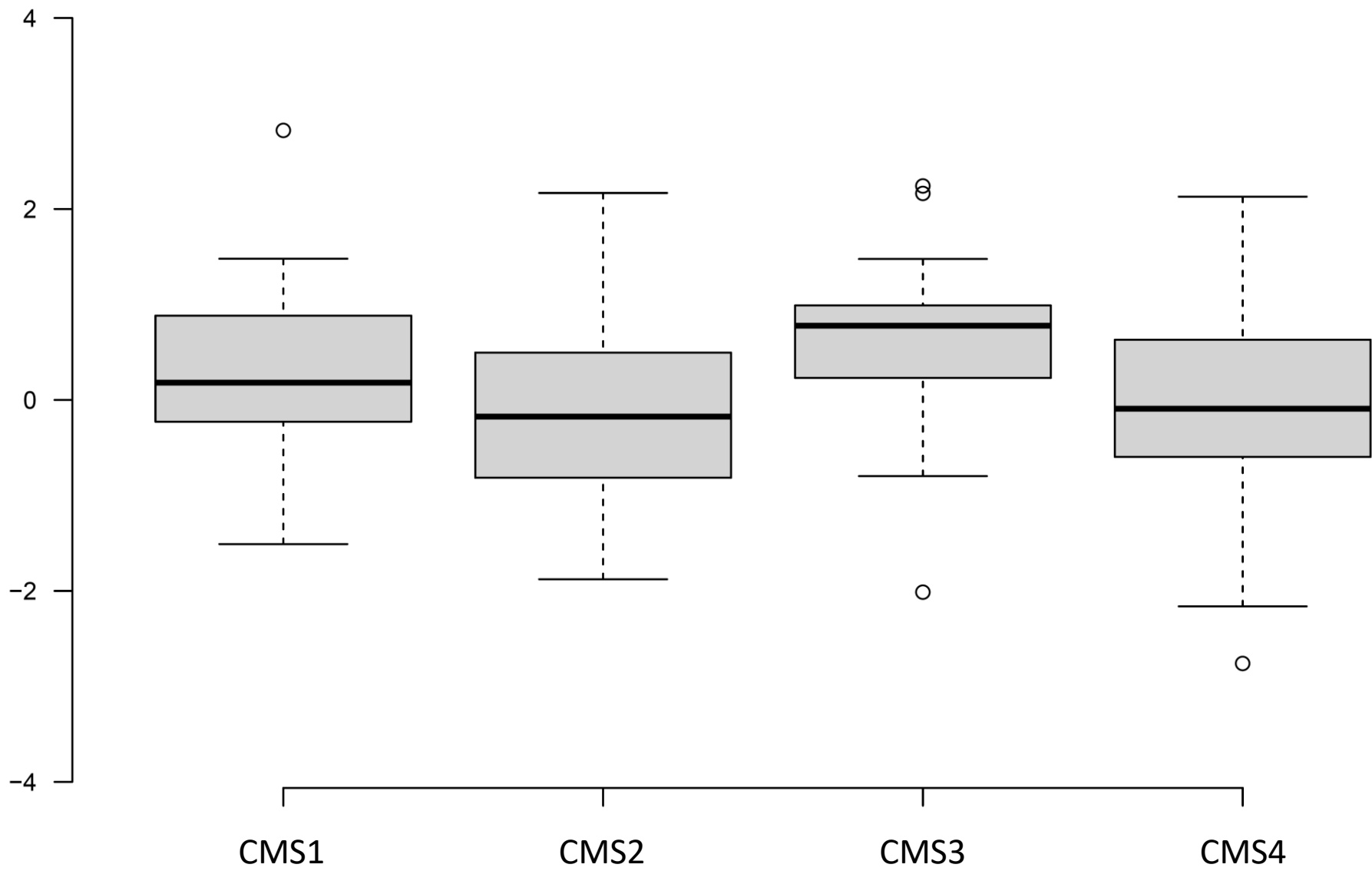

Supplement: Supplementary file 1 [file ijms-24-05797-s001.zip › Figure S1_CMS related CA9-CA12 expression in CRC samples/CA12_Roepman_GSE42284.pdf]

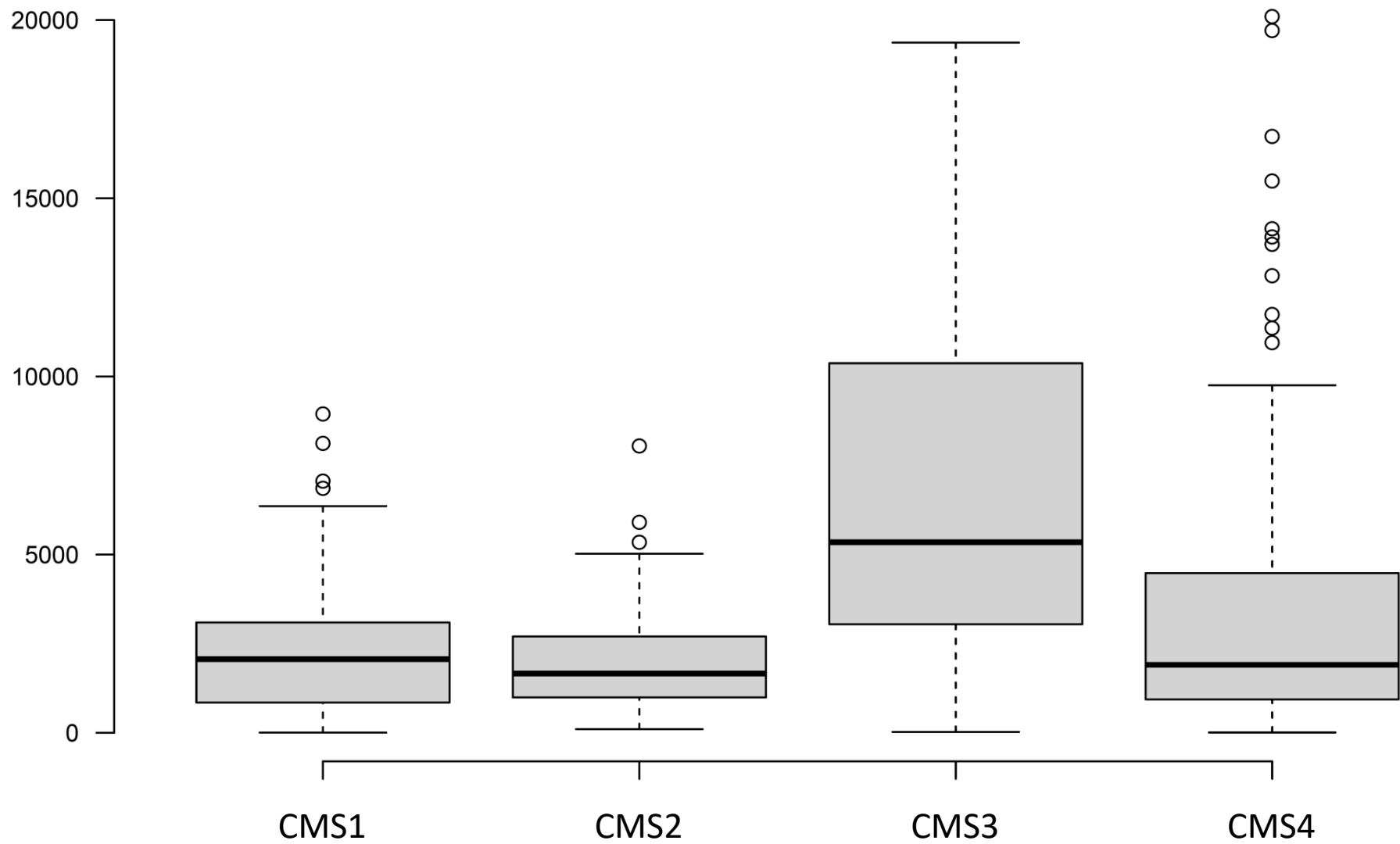

Supplement: Supplementary file 1 [file ijms-24-05797-s001.zip › Figure S1_CMS related CA9-CA12 expression in CRC samples/CA12_TCGA.pdf]

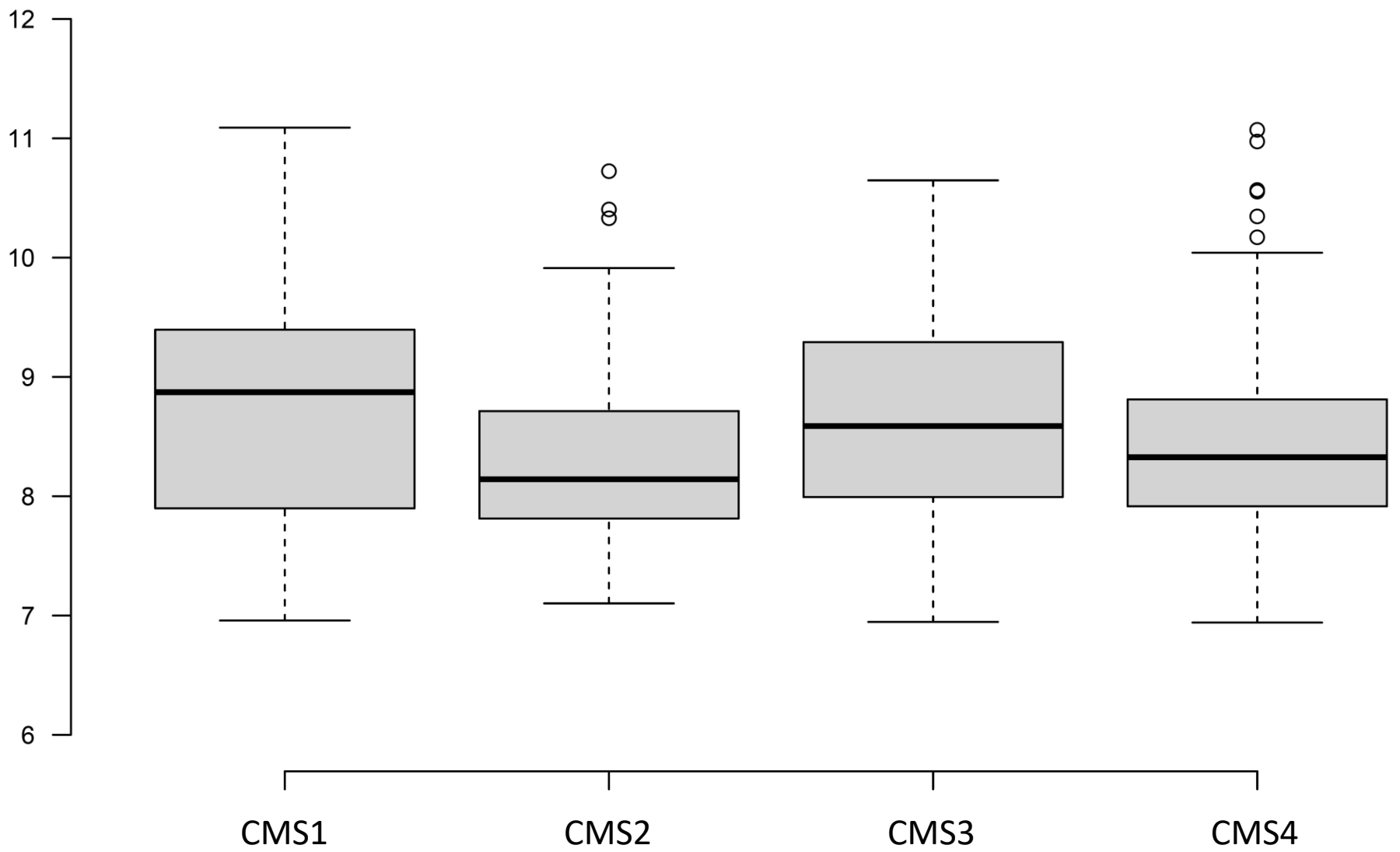

Supplement: Supplementary file 1 [file ijms-24-05797-s001.zip › Figure S1_CMS related CA9-CA12 expression in CRC samples/CA9_Jorissen_GSE14333.pdf]

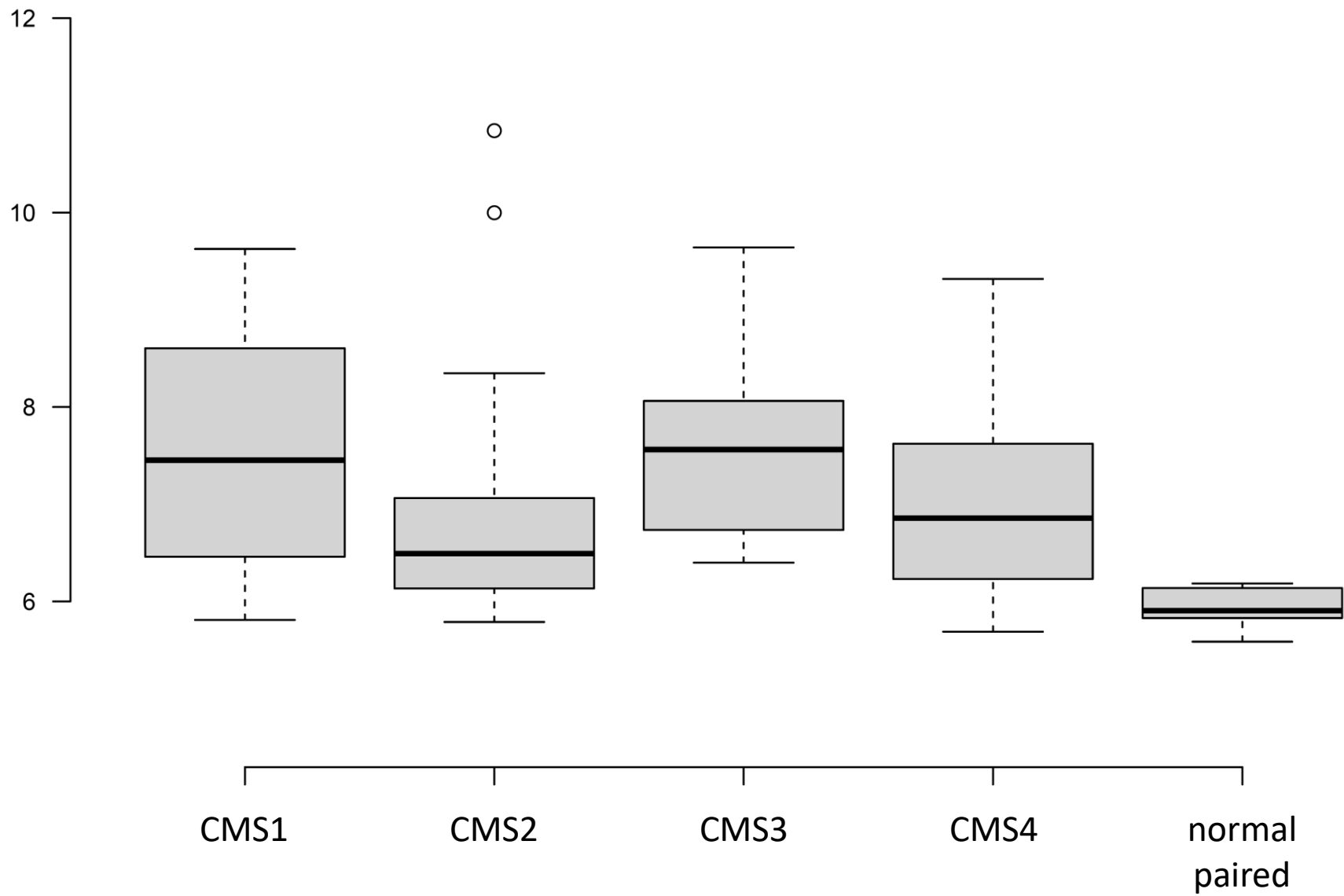

Supplement: Supplementary file 1 [file ijms-24-05797-s001.zip › Figure S1_CMS related CA9-CA12 expression in CRC samples/CA9_Kemper_GSE33113.pdf]

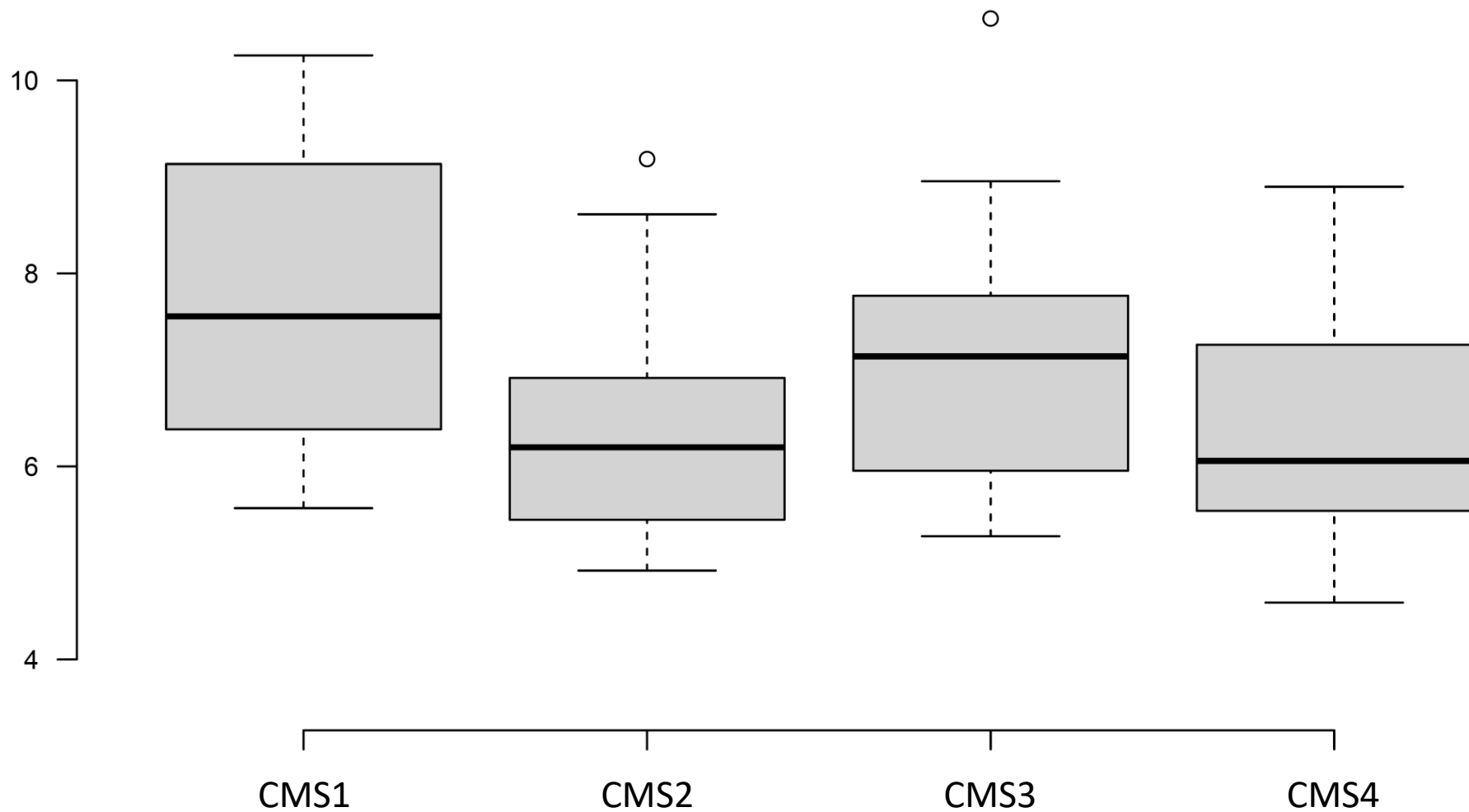

Supplement: Supplementary file 1 [file ijms-24-05797-s001.zip › Figure S1_CMS related CA9-CA12 expression in CRC samples/CA9_Laibe_GSE37892.pdf]

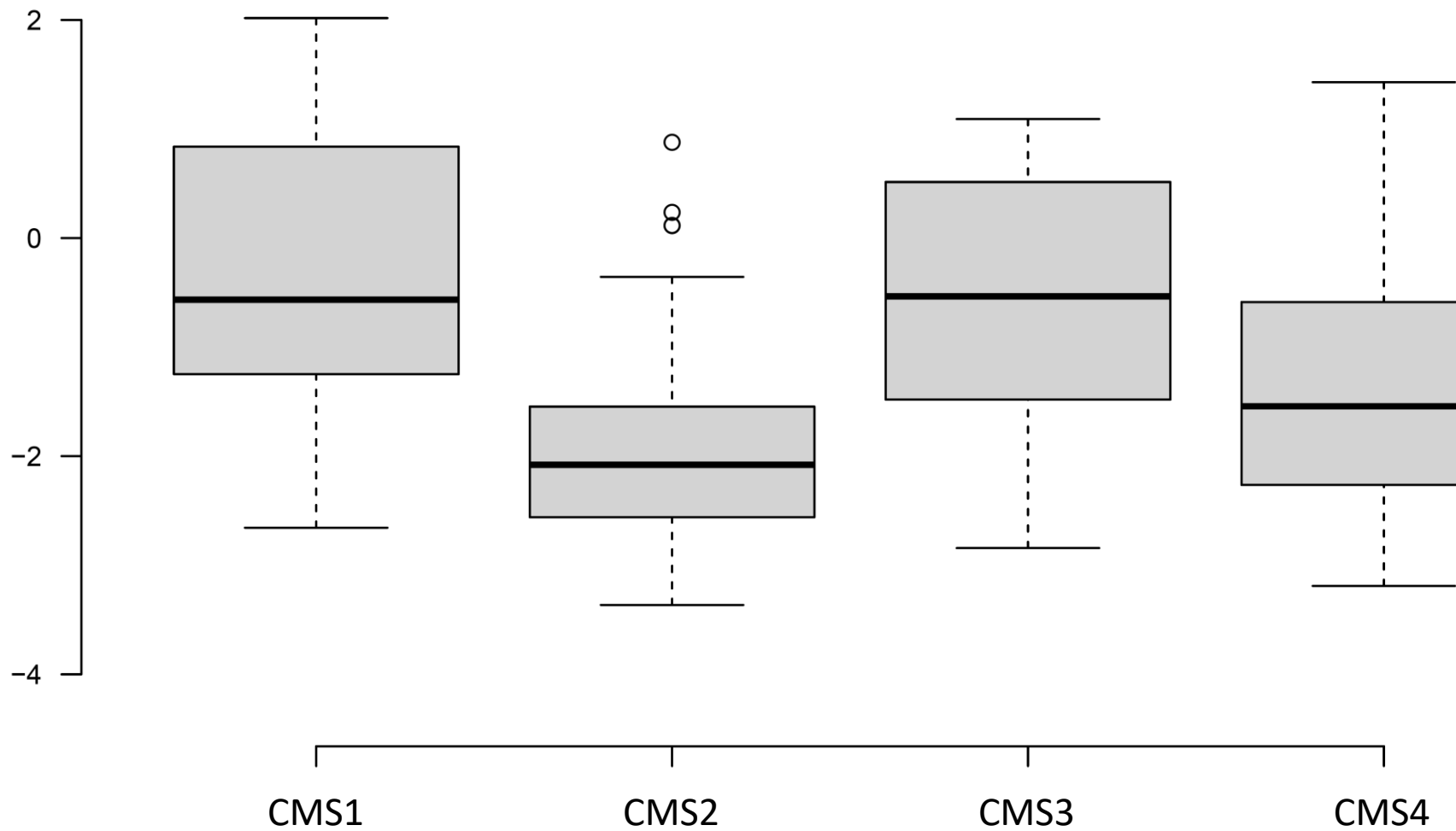

Supplement: Supplementary file 1 [file ijms-24-05797-s001.zip › Figure S1_CMS related CA9-CA12 expression in CRC samples/CA9_Roepman_GSE42284.pdf]

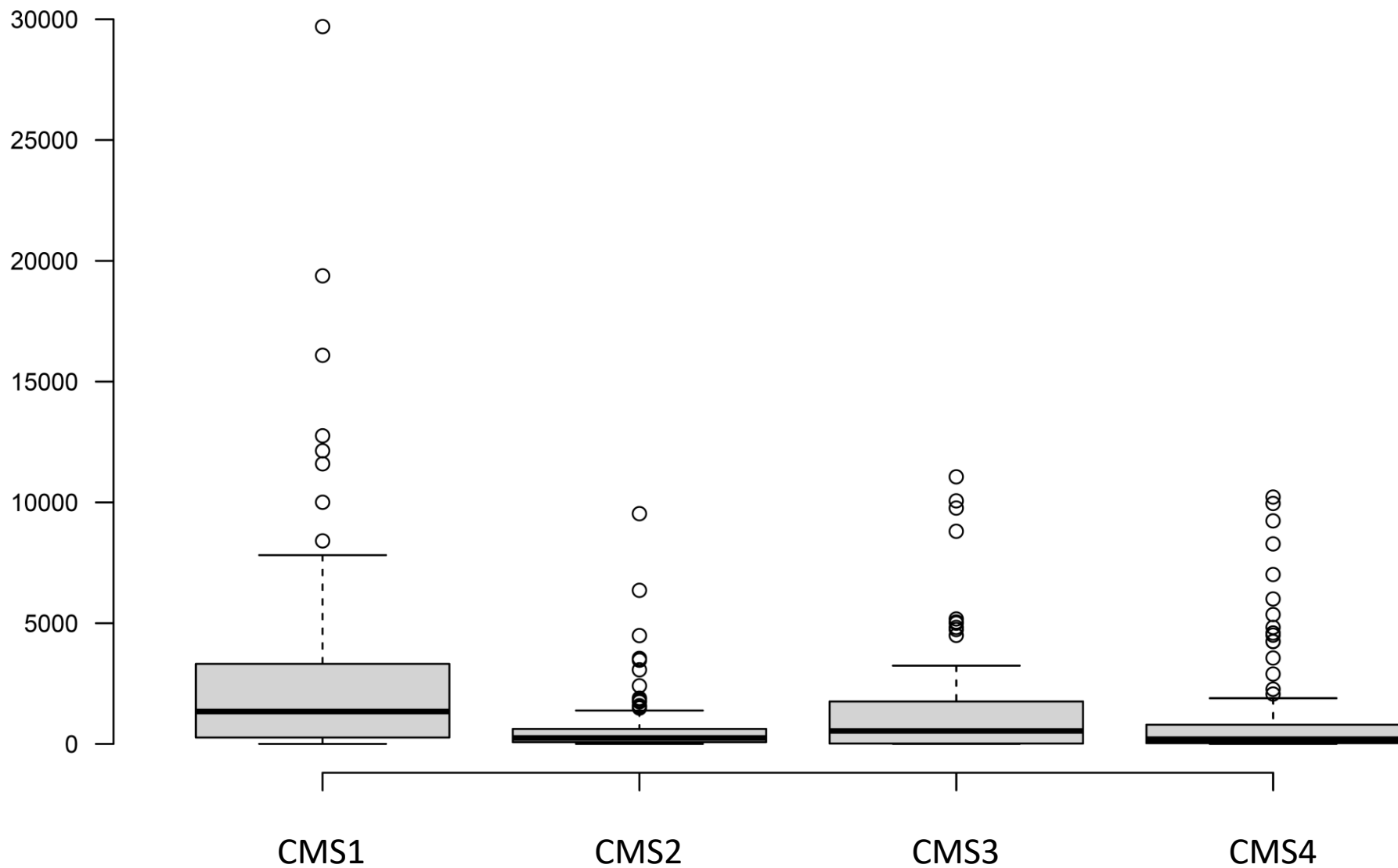

Supplement: Supplementary file 1 [file ijms-24-05797-s001.zip › Figure S1_CMS related CA9-CA12 expression in CRC samples/CA9_TCGA.pdf]

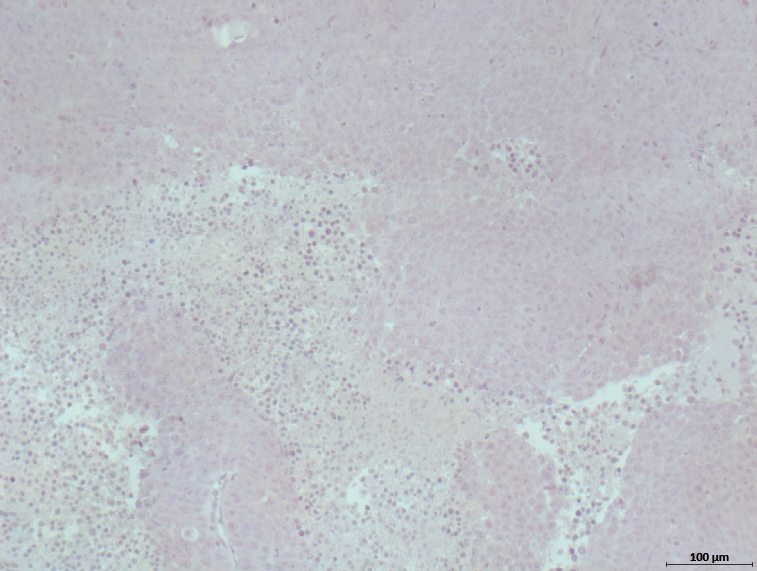

Supplement: Supplementary file 1 [file ijms-24-05797-s001.zip › Figure S2_CA9-CA12 immunohistochemistry - original pictures/HCT116/CA12 10x.tif]

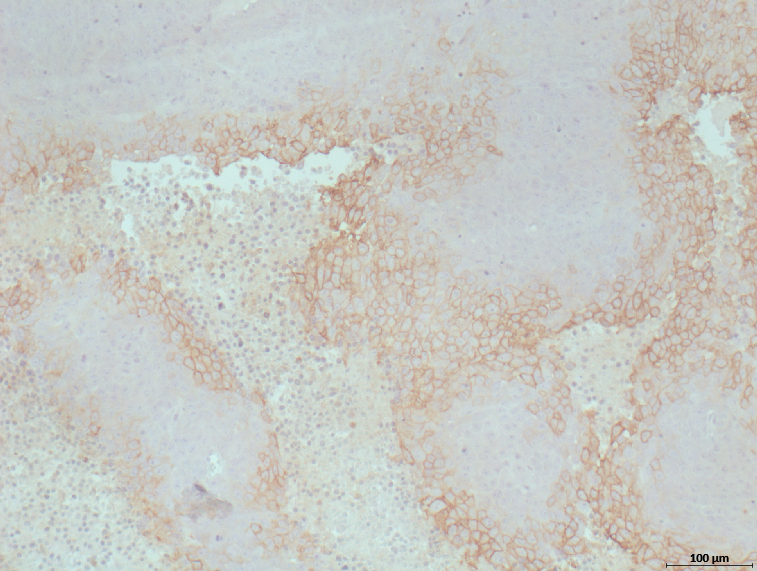

Supplement: Supplementary file 1 [file ijms-24-05797-s001.zip › Figure S2_CA9-CA12 immunohistochemistry - original pictures/HCT116/CA9 10x.tif]

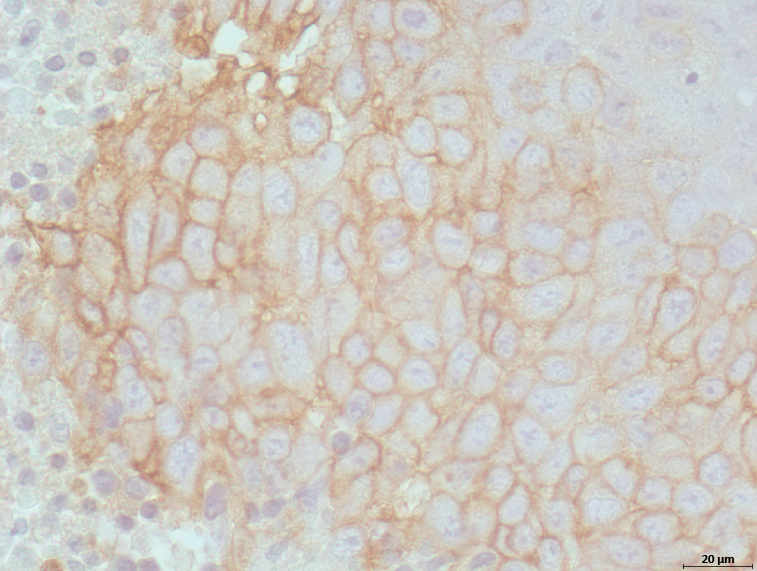

Supplement: Supplementary file 1 [file ijms-24-05797-s001.zip › Figure S2_CA9-CA12 immunohistochemistry - original pictures/HCT116/CA9 40x.tif]

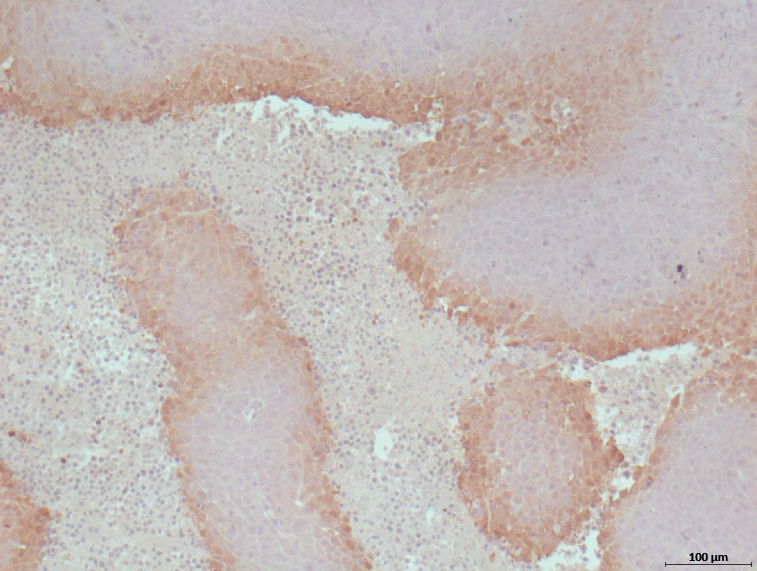

Supplement: Supplementary file 1 [file ijms-24-05797-s001.zip › Figure S2_CA9-CA12 immunohistochemistry - original pictures/HCT116/HP 10x.tif]

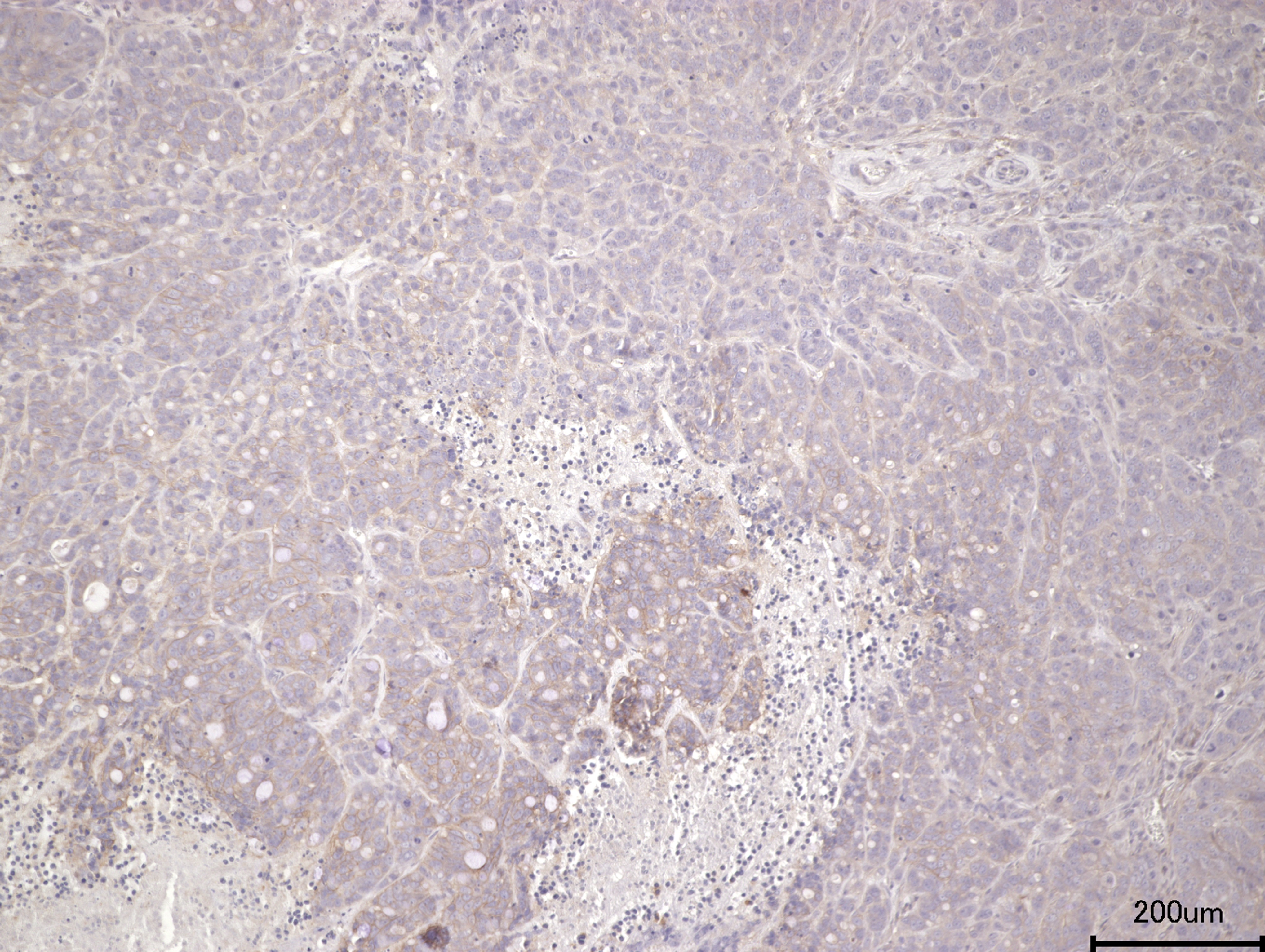

Supplement: Supplementary file 1 [file ijms-24-05797-s001.zip › Figure S2_CA9-CA12 immunohistochemistry - original pictures/HT29/additional pictures/CA12 10x.jpg]

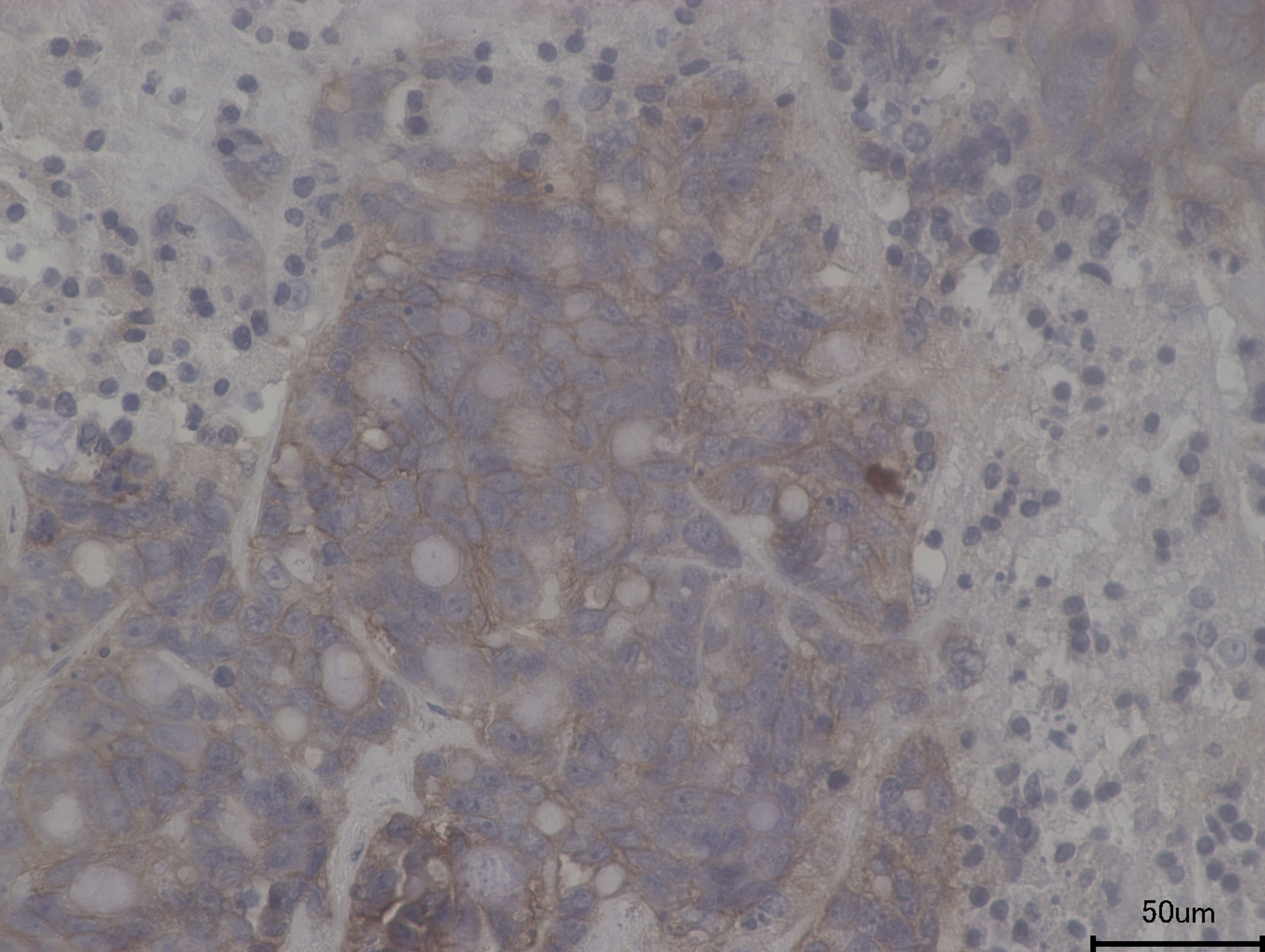

Supplement: Supplementary file 1 [file ijms-24-05797-s001.zip › Figure S2_CA9-CA12 immunohistochemistry - original pictures/HT29/additional pictures/CA12 40x perinecrotic.jpg]

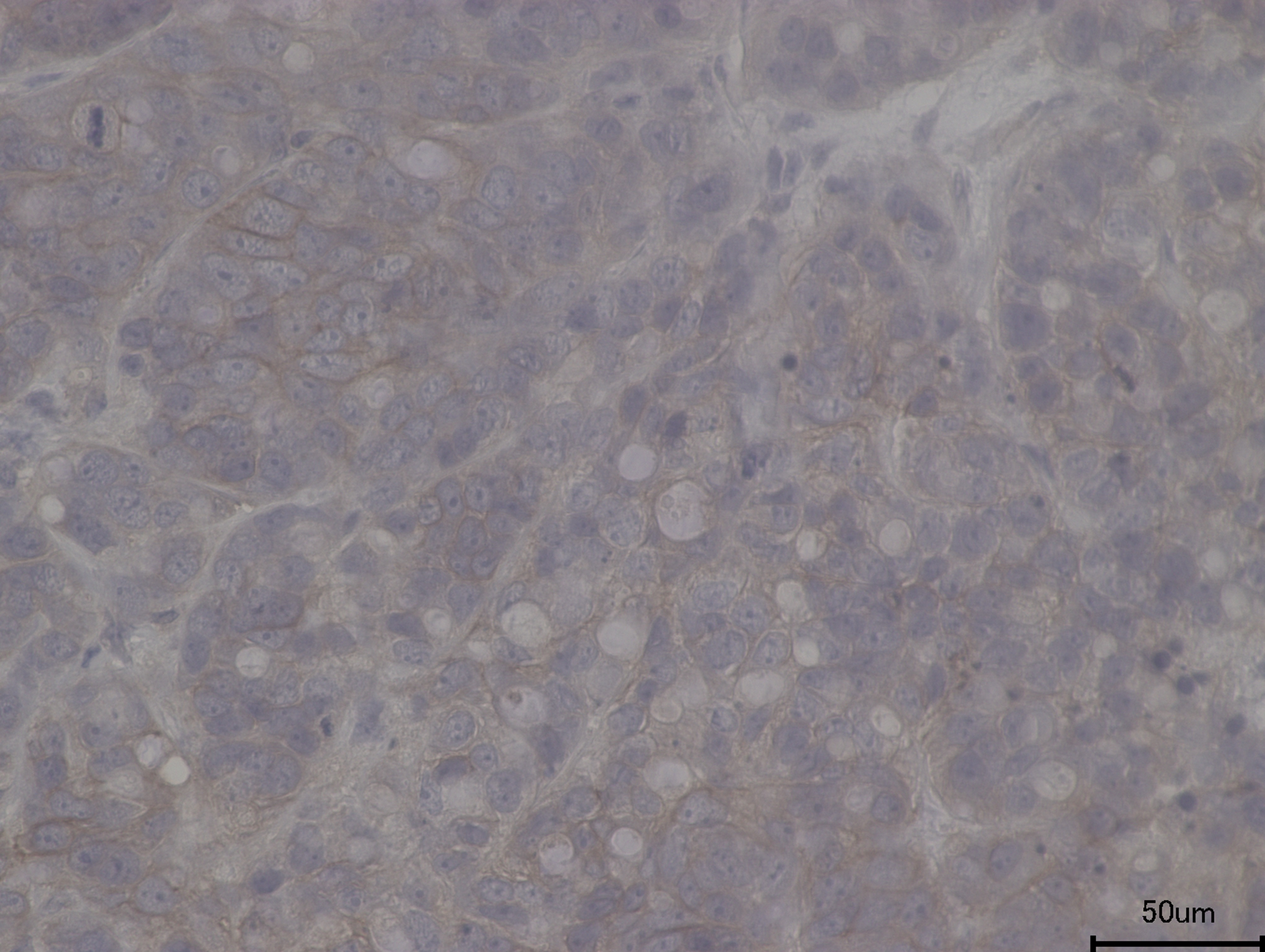

Supplement: Supplementary file 1 [file ijms-24-05797-s001.zip › Figure S2_CA9-CA12 immunohistochemistry - original pictures/HT29/additional pictures/CA12 40x vital.jpg]

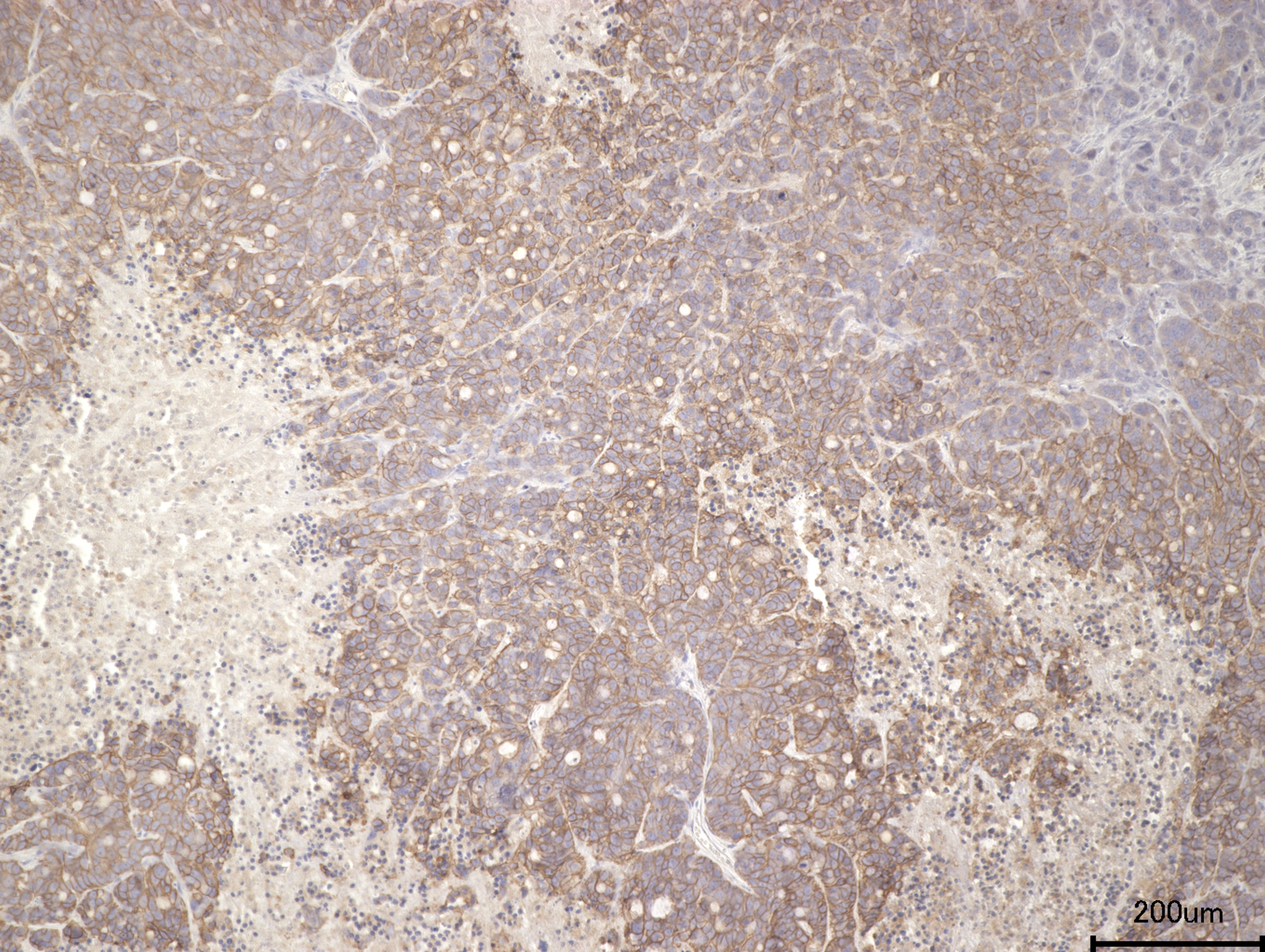

Supplement: Supplementary file 1 [file ijms-24-05797-s001.zip › Figure S2_CA9-CA12 immunohistochemistry - original pictures/HT29/additional pictures/CA9 Rabbit 10x.jpg]

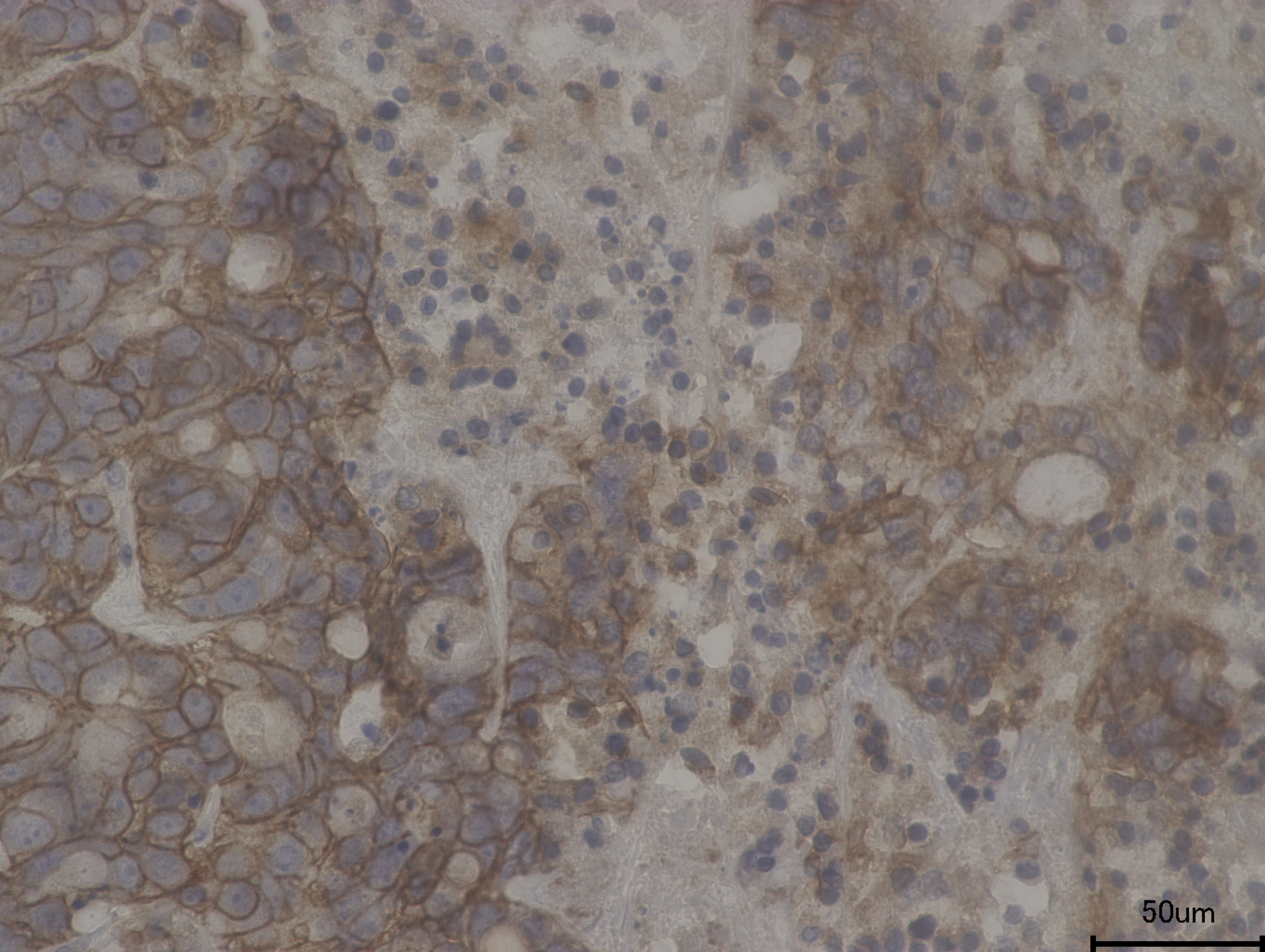

Supplement: Supplementary file 1 [file ijms-24-05797-s001.zip › Figure S2_CA9-CA12 immunohistochemistry - original pictures/HT29/additional pictures/CA9 Rabbit 40x perinecrotic.jpg]

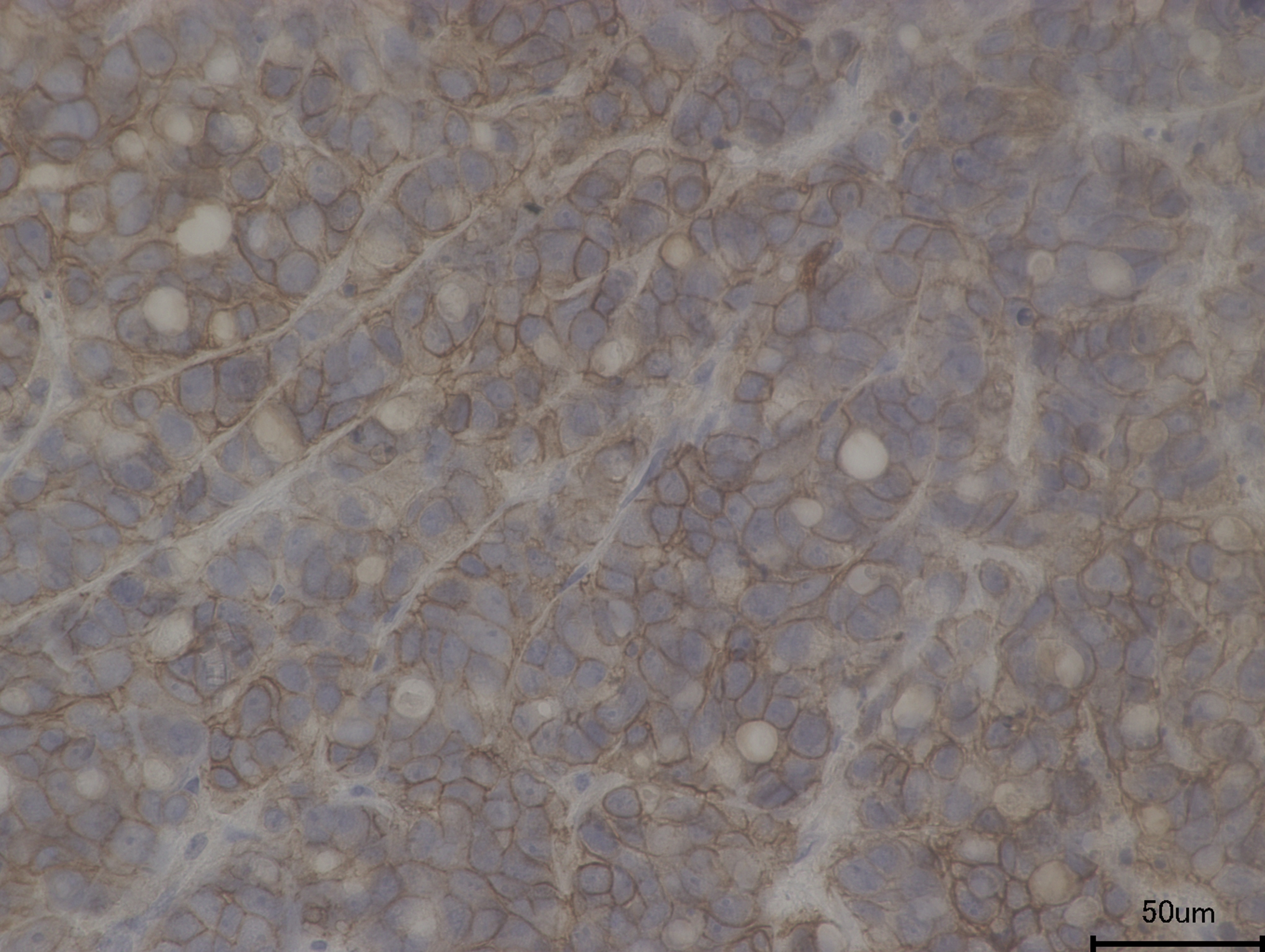

Supplement: Supplementary file 1 [file ijms-24-05797-s001.zip › Figure S2_CA9-CA12 immunohistochemistry - original pictures/HT29/additional pictures/CA9 Rabbit 40x vital.jpg]

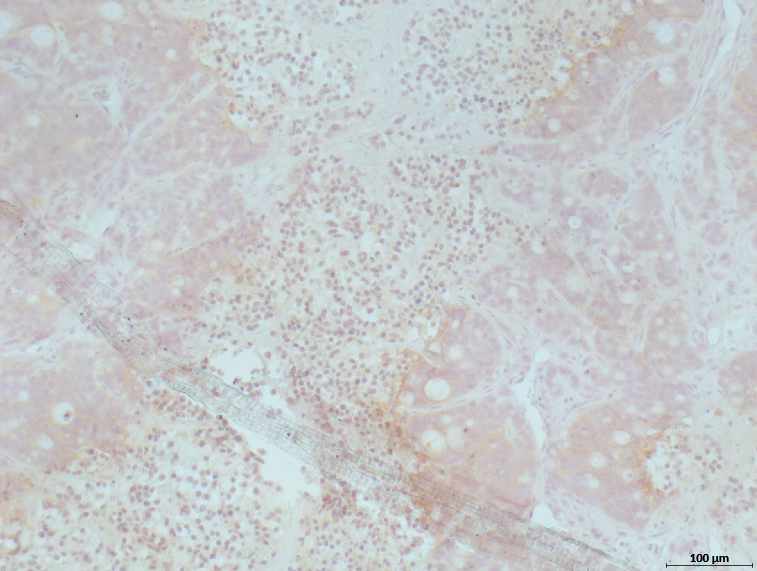

Supplement: Supplementary file 1 [file ijms-24-05797-s001.zip › Figure S2_CA9-CA12 immunohistochemistry - original pictures/HT29/CA12 10x.tif]

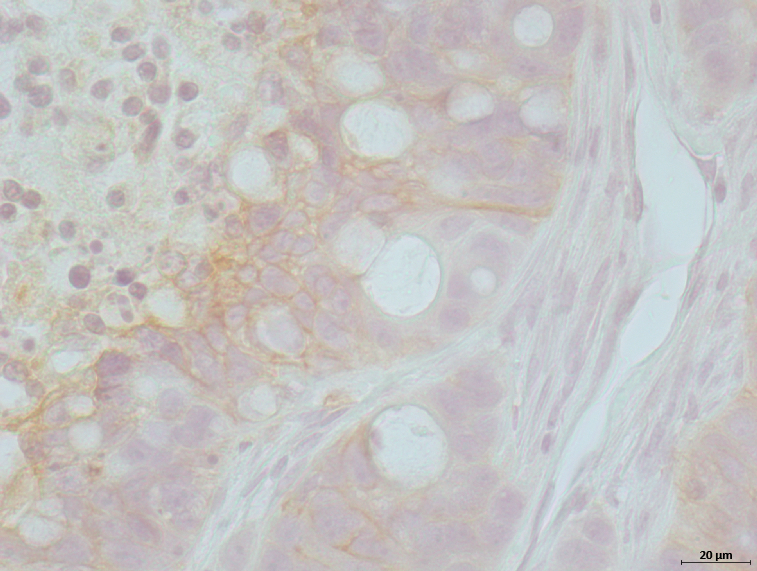

Supplement: Supplementary file 1 [file ijms-24-05797-s001.zip › Figure S2_CA9-CA12 immunohistochemistry - original pictures/HT29/CA12 40x.tif]

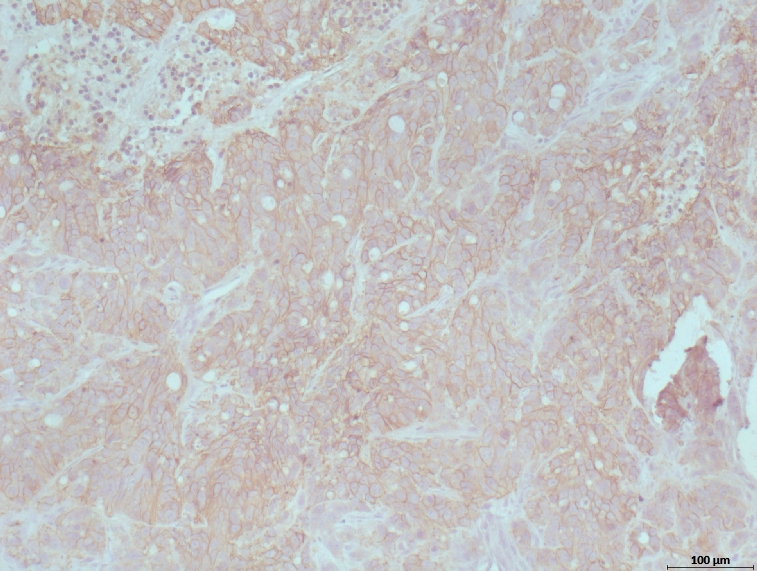

Supplement: Supplementary file 1 [file ijms-24-05797-s001.zip › Figure S2_CA9-CA12 immunohistochemistry - original pictures/HT29/CA9 10x.jpg]

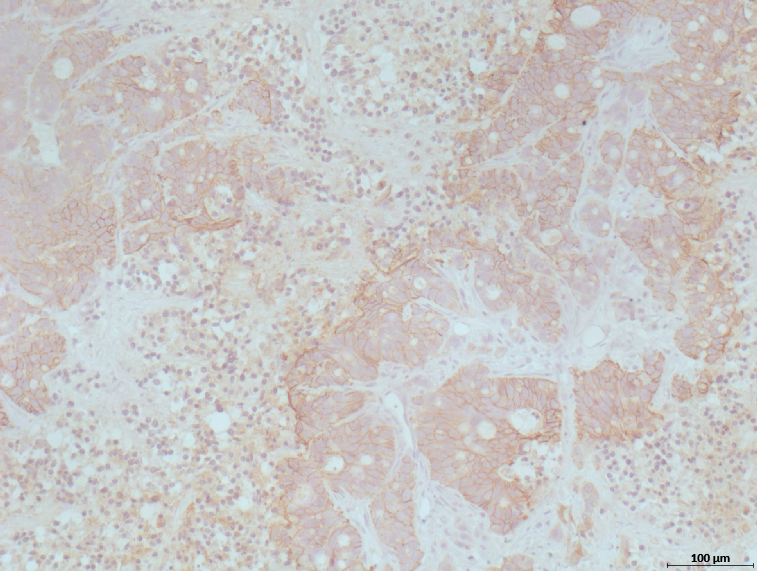

Supplement: Supplementary file 1 [file ijms-24-05797-s001.zip › Figure S2_CA9-CA12 immunohistochemistry - original pictures/HT29/CA9 10x.tif]

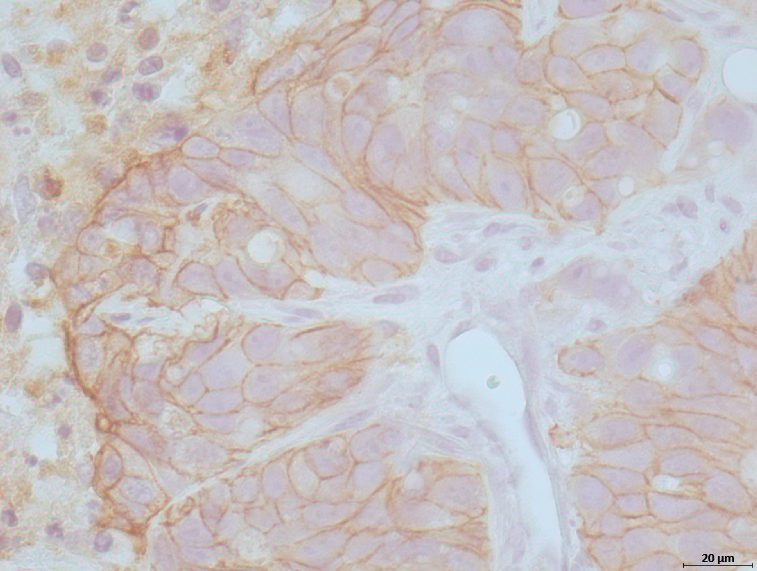

Supplement: Supplementary file 1 [file ijms-24-05797-s001.zip › Figure S2_CA9-CA12 immunohistochemistry - original pictures/HT29/CA9 40x.tif]

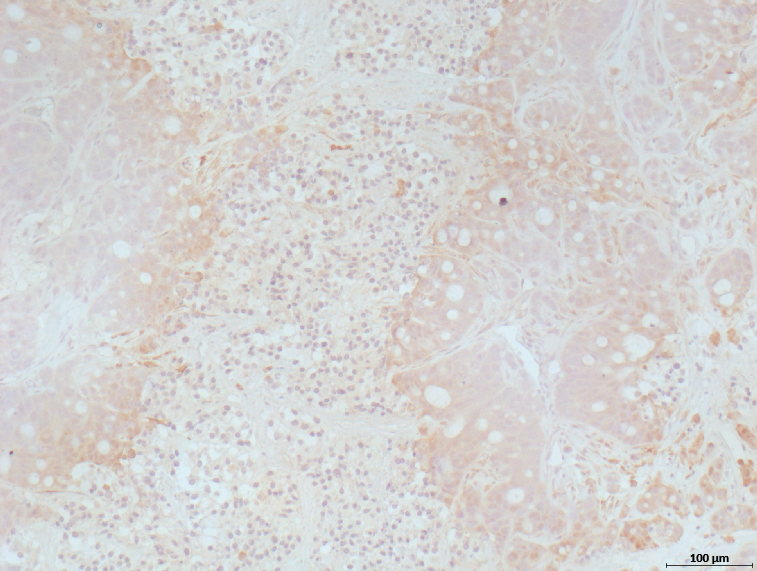

Supplement: Supplementary file 1 [file ijms-24-05797-s001.zip › Figure S2_CA9-CA12 immunohistochemistry - original pictures/HT29/HP 10x.tif]

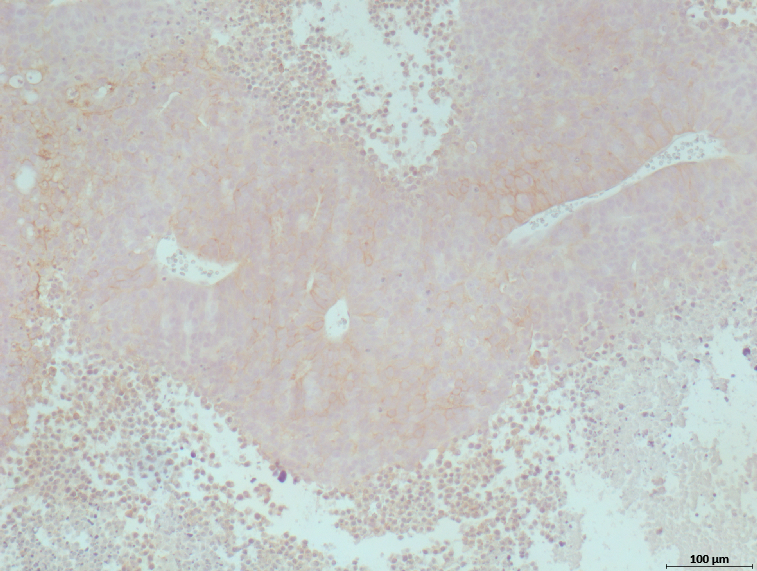

Supplement: Supplementary file 1 [file ijms-24-05797-s001.zip › Figure S2_CA9-CA12 immunohistochemistry - original pictures/LOVO/CA12 10x.tif]

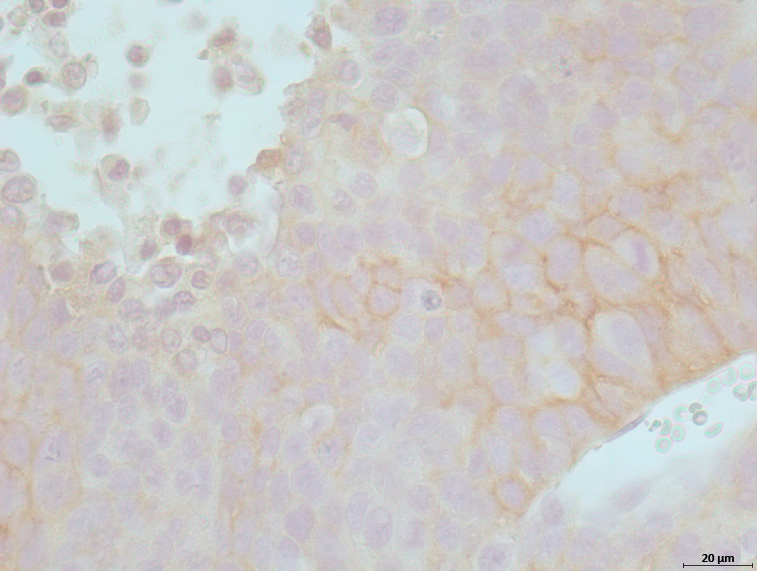

Supplement: Supplementary file 1 [file ijms-24-05797-s001.zip › Figure S2_CA9-CA12 immunohistochemistry - original pictures/LOVO/CA12 40x.tif]

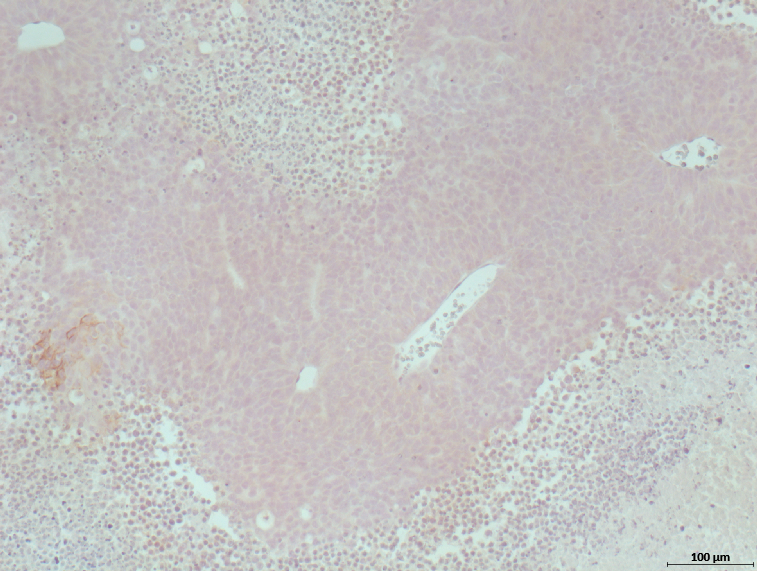

Supplement: Supplementary file 1 [file ijms-24-05797-s001.zip › Figure S2_CA9-CA12 immunohistochemistry - original pictures/LOVO/CA9 10x.tif]

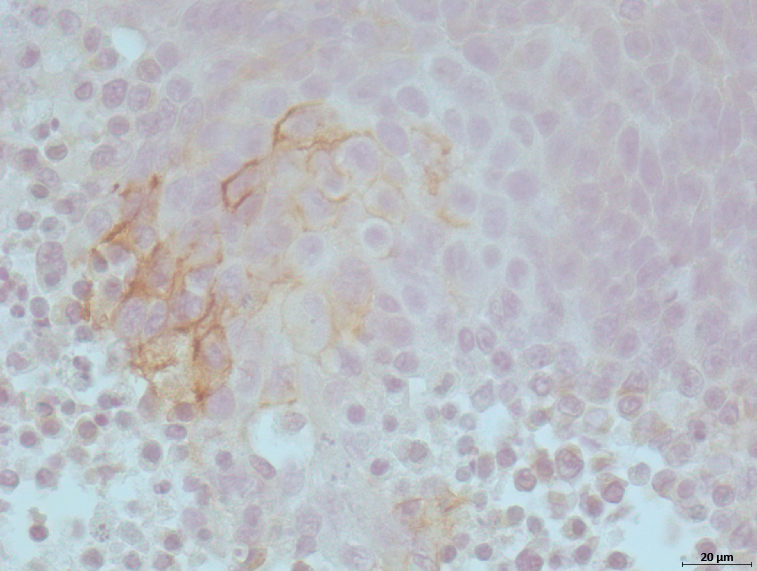

Supplement: Supplementary file 1 [file ijms-24-05797-s001.zip › Figure S2_CA9-CA12 immunohistochemistry - original pictures/LOVO/CA9 40x.tif]

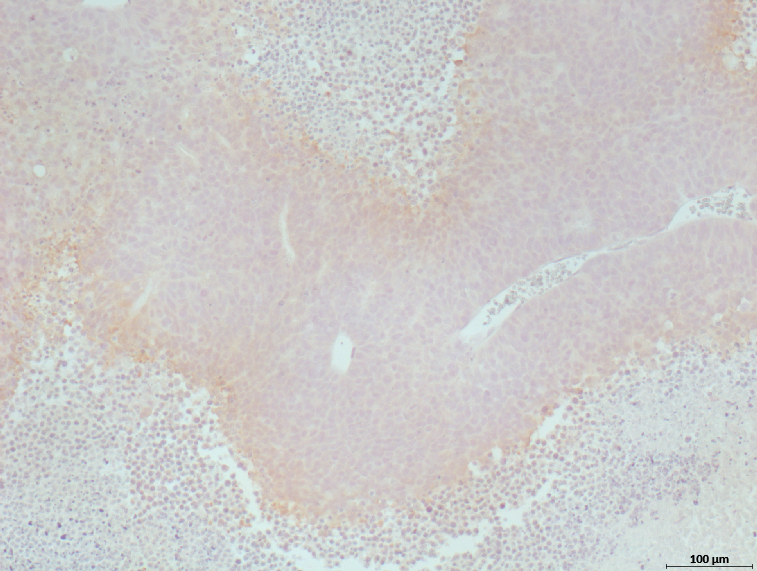

Supplement: Supplementary file 1 [file ijms-24-05797-s001.zip › Figure S2_CA9-CA12 immunohistochemistry - original pictures/LOVO/HP 10x.tif]

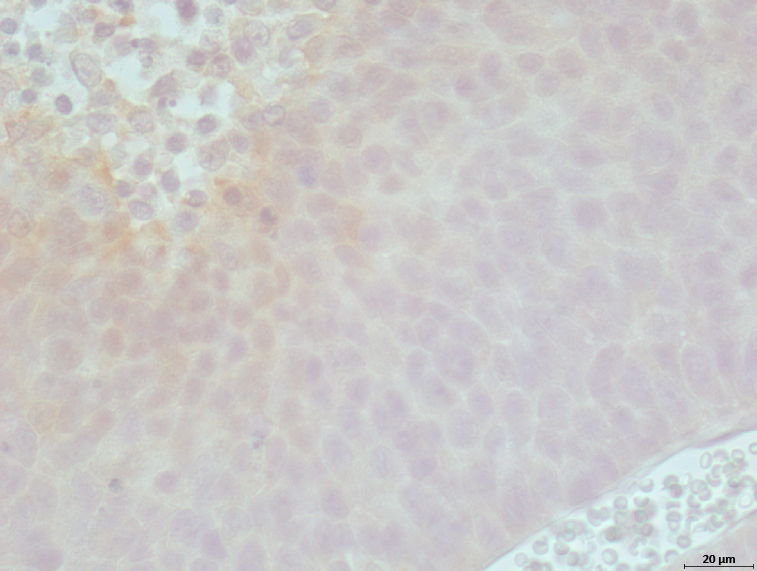

Supplement: Supplementary file 1 [file ijms-24-05797-s001.zip › Figure S2_CA9-CA12 immunohistochemistry - original pictures/LOVO/HP40x.tif]

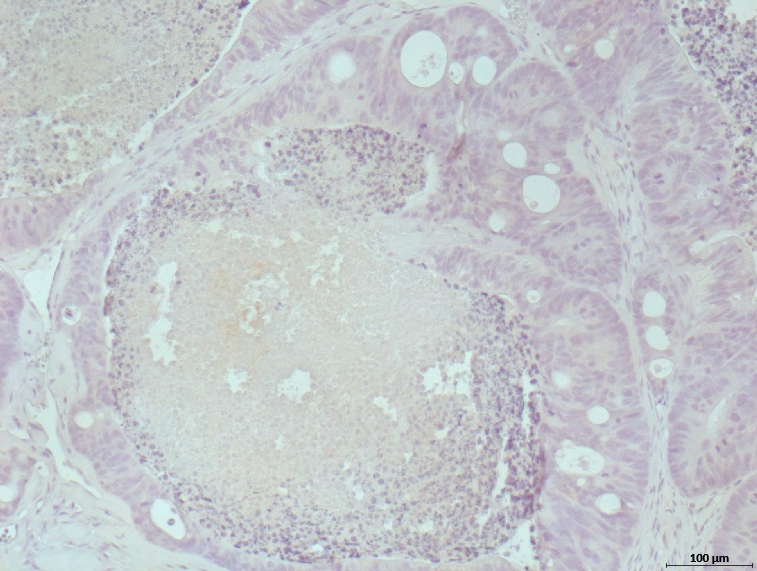

Supplement: Supplementary file 1 [file ijms-24-05797-s001.zip › Figure S2_CA9-CA12 immunohistochemistry - original pictures/LS1034/CA12 10x.jpeg]

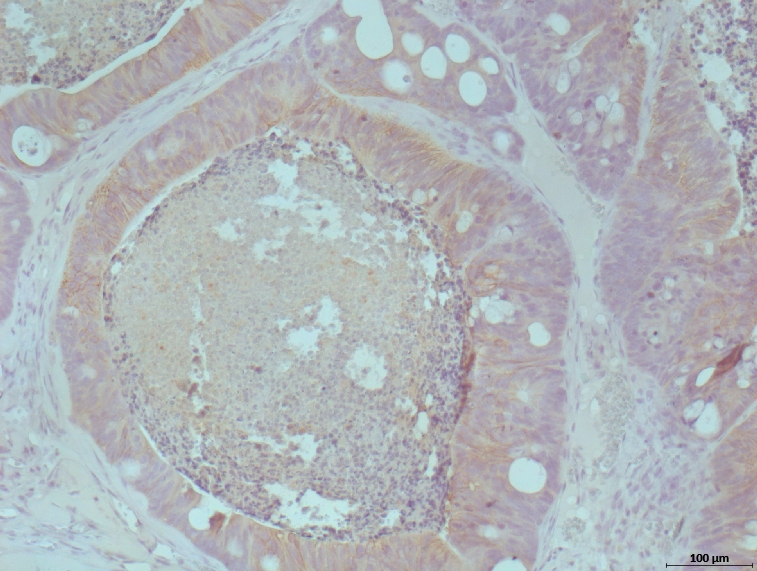

Supplement: Supplementary file 1 [file ijms-24-05797-s001.zip › Figure S2_CA9-CA12 immunohistochemistry - original pictures/LS1034/CA9 10x.jpeg]

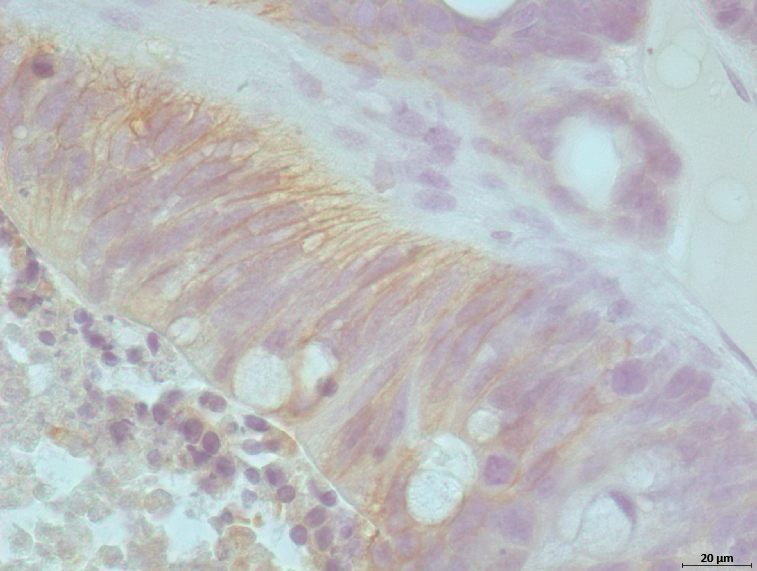

Supplement: Supplementary file 1 [file ijms-24-05797-s001.zip › Figure S2_CA9-CA12 immunohistochemistry - original pictures/LS1034/CA9 40x.jpeg]

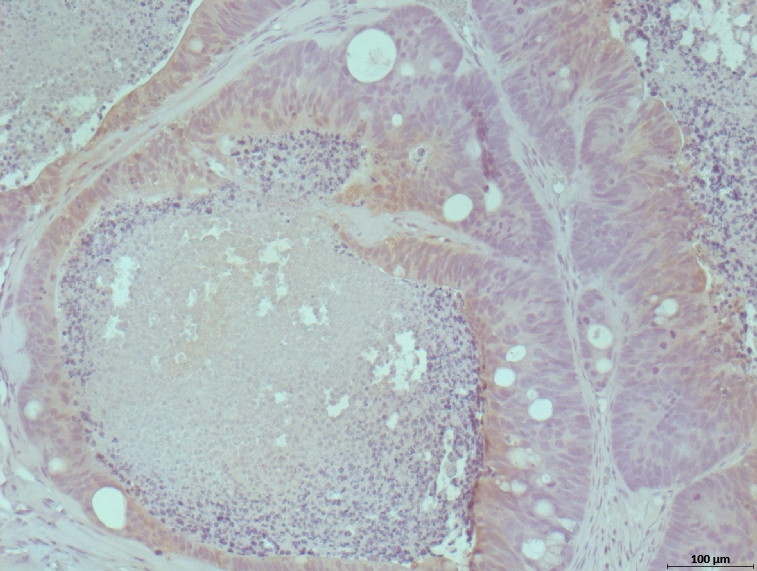

Supplement: Supplementary file 1 [file ijms-24-05797-s001.zip › Figure S2_CA9-CA12 immunohistochemistry - original pictures/LS1034/HP 10x.jpeg]

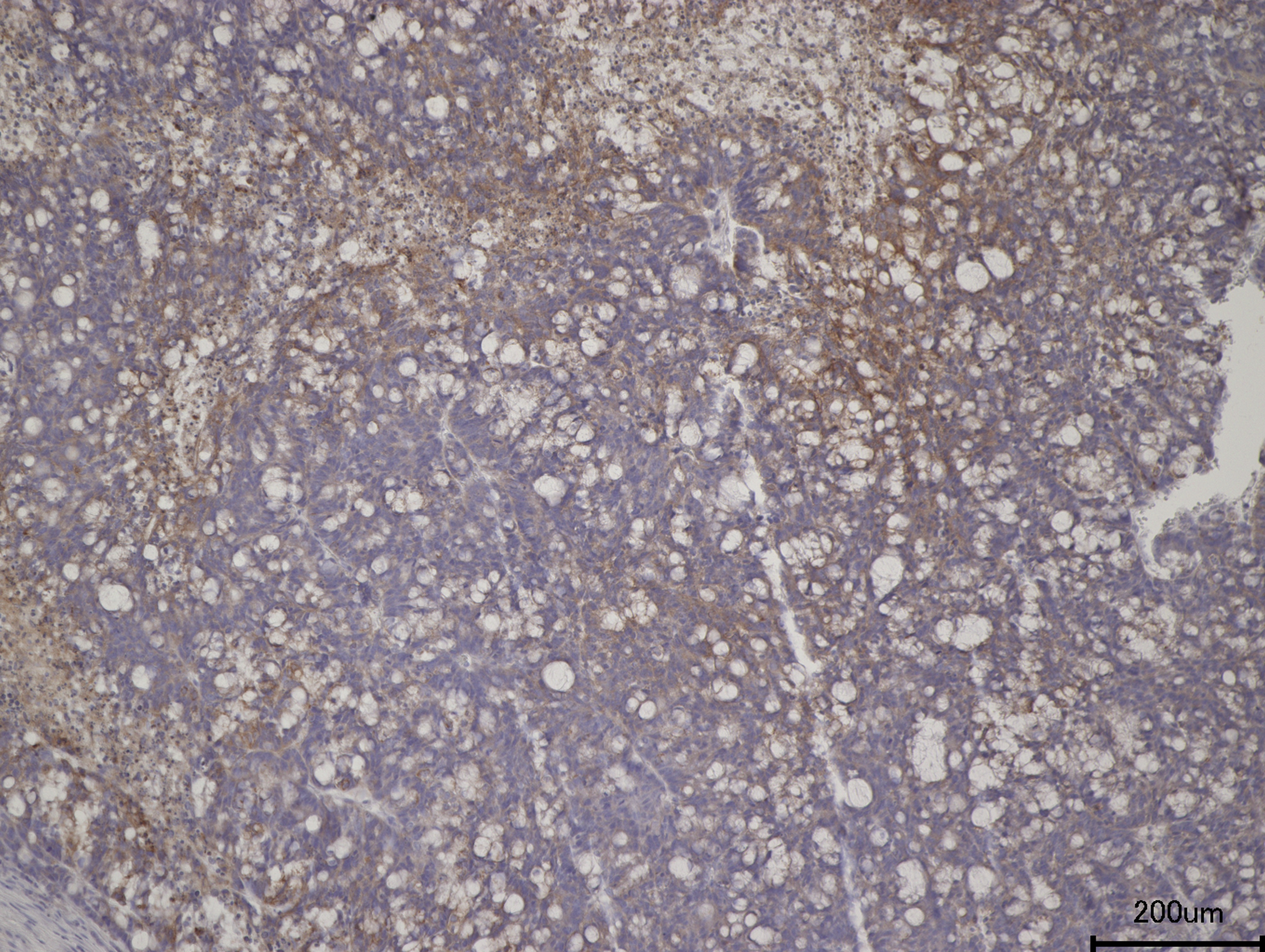

Supplement: Supplementary file 1 [file ijms-24-05797-s001.zip › Figure S2_CA9-CA12 immunohistochemistry - original pictures/LS174T/additional pictures/CA12 10x.jpg]

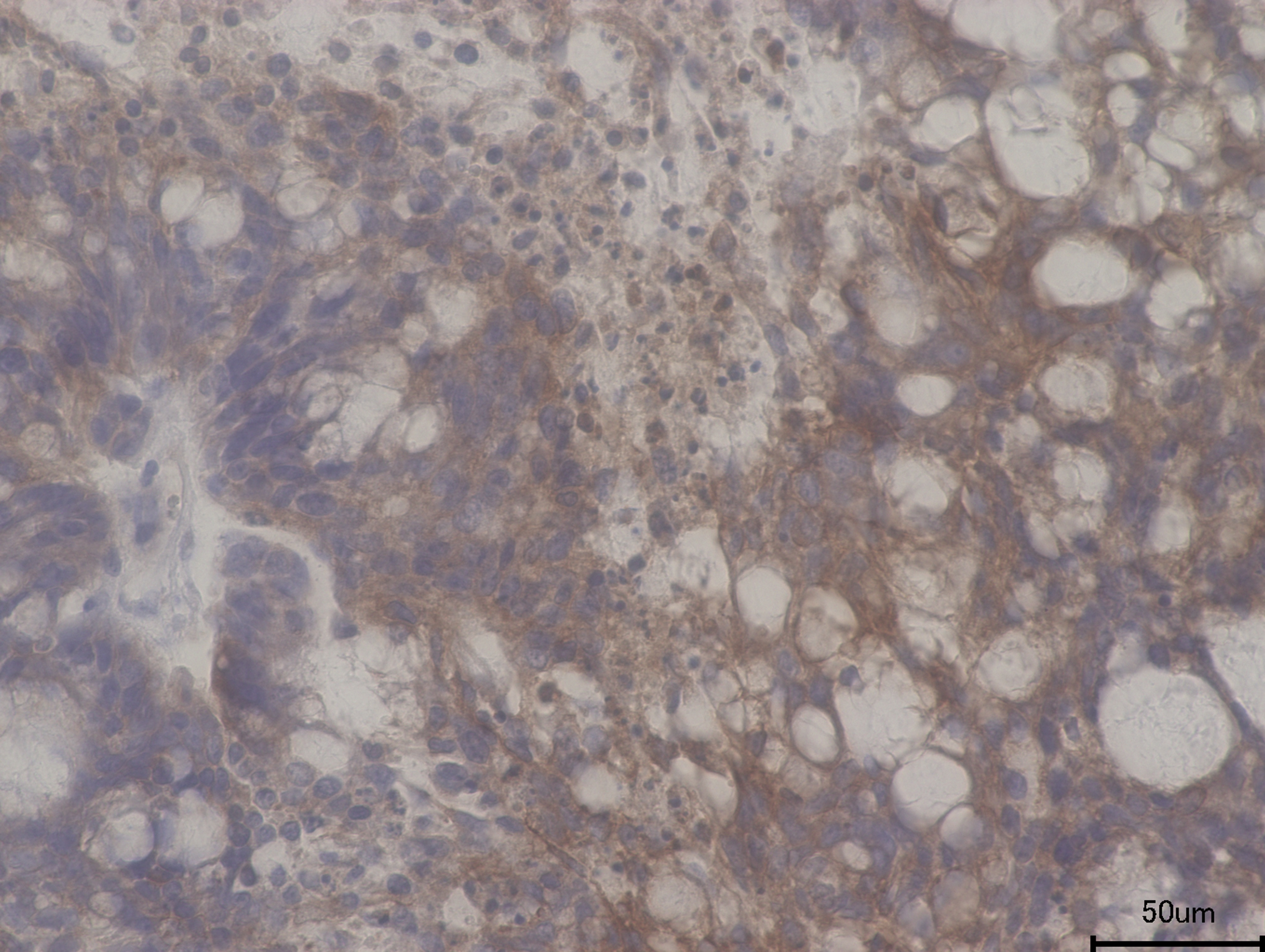

Supplement: Supplementary file 1 [file ijms-24-05797-s001.zip › Figure S2_CA9-CA12 immunohistochemistry - original pictures/LS174T/additional pictures/CA12 40x perinecrotic.jpg]

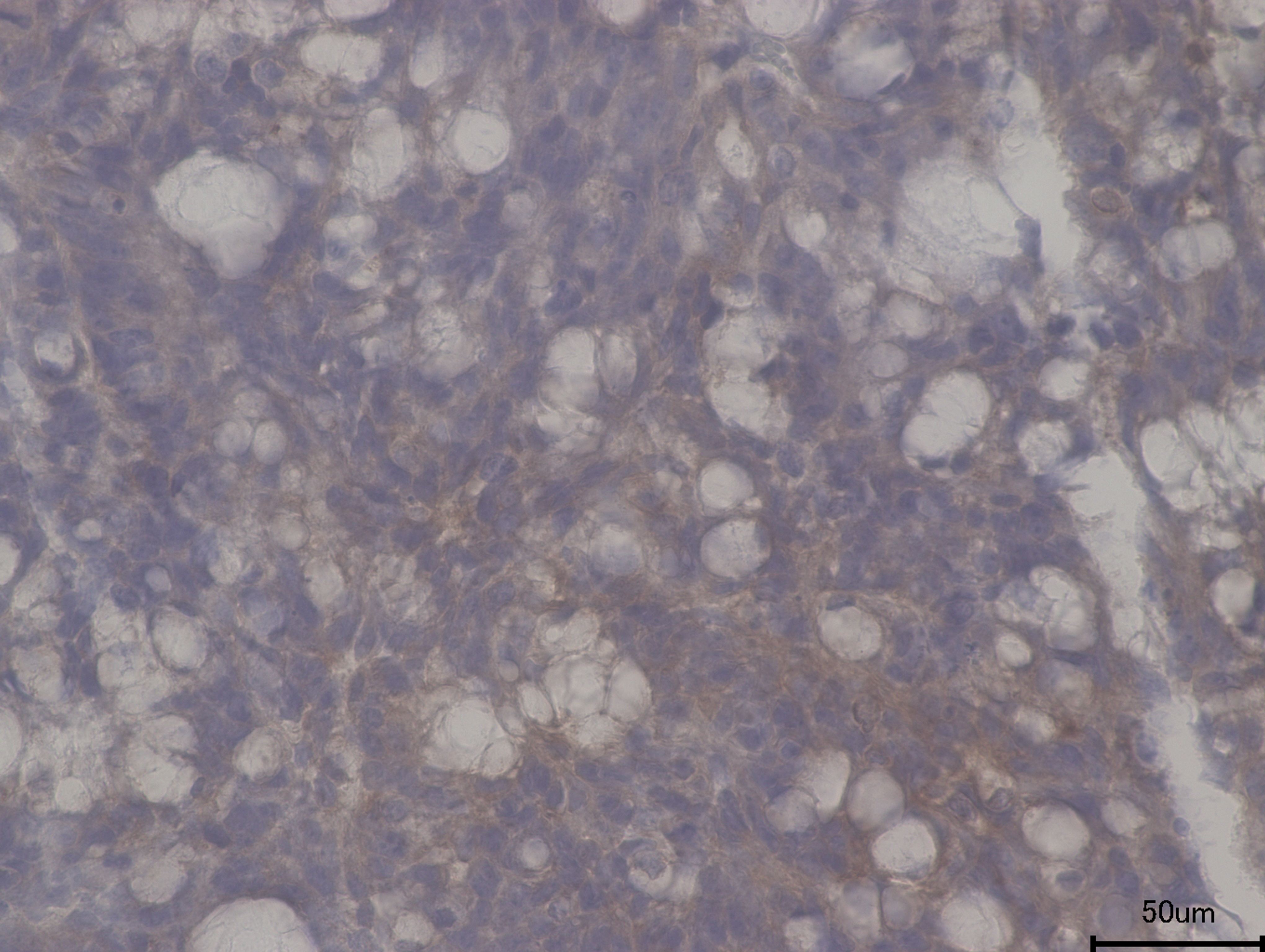

Supplement: Supplementary file 1 [file ijms-24-05797-s001.zip › Figure S2_CA9-CA12 immunohistochemistry - original pictures/LS174T/additional pictures/CA12 40x vital.jpg]

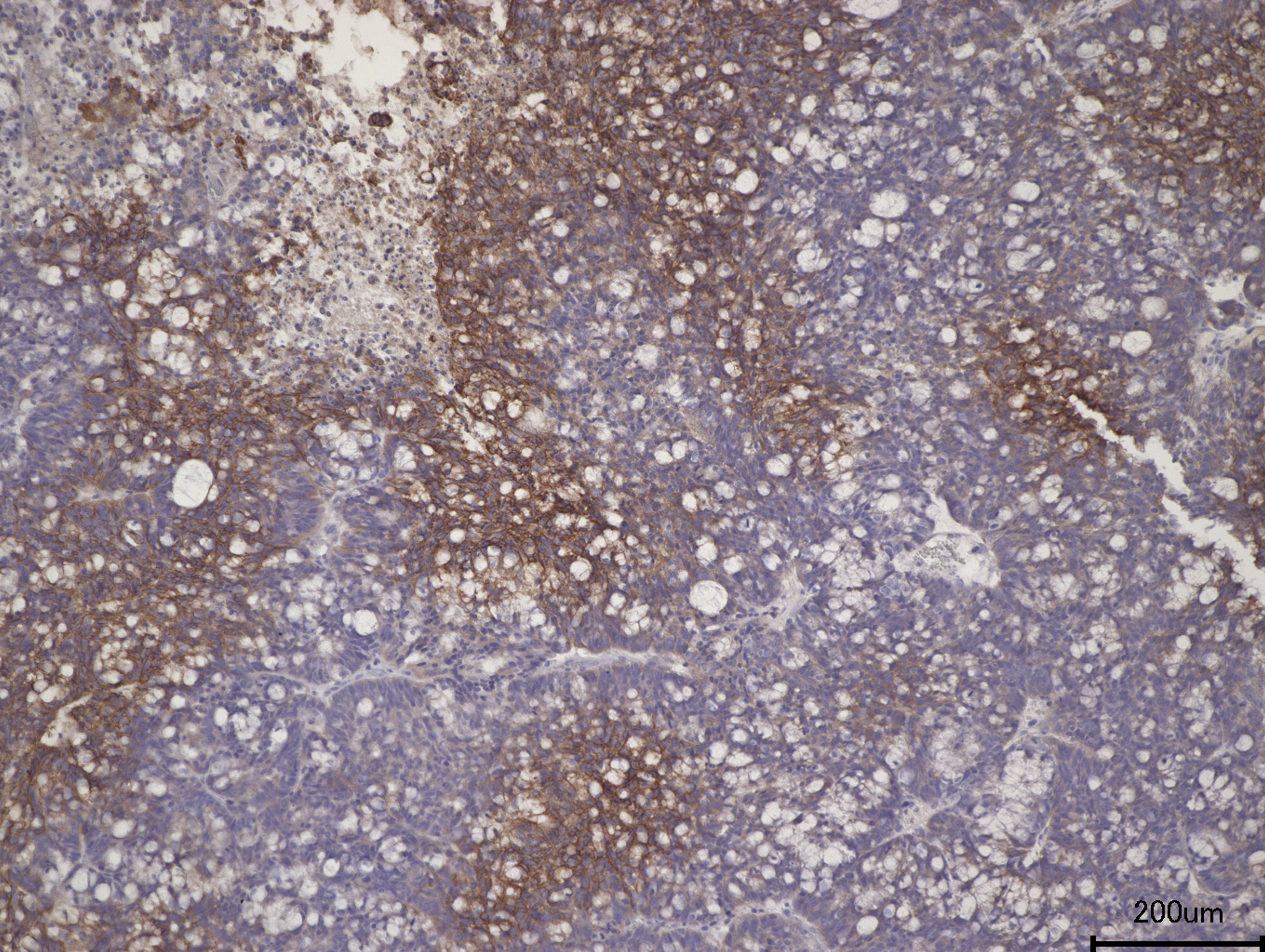

Supplement: Supplementary file 1 [file ijms-24-05797-s001.zip › Figure S2_CA9-CA12 immunohistochemistry - original pictures/LS174T/additional pictures/CA9 10x.jpg]

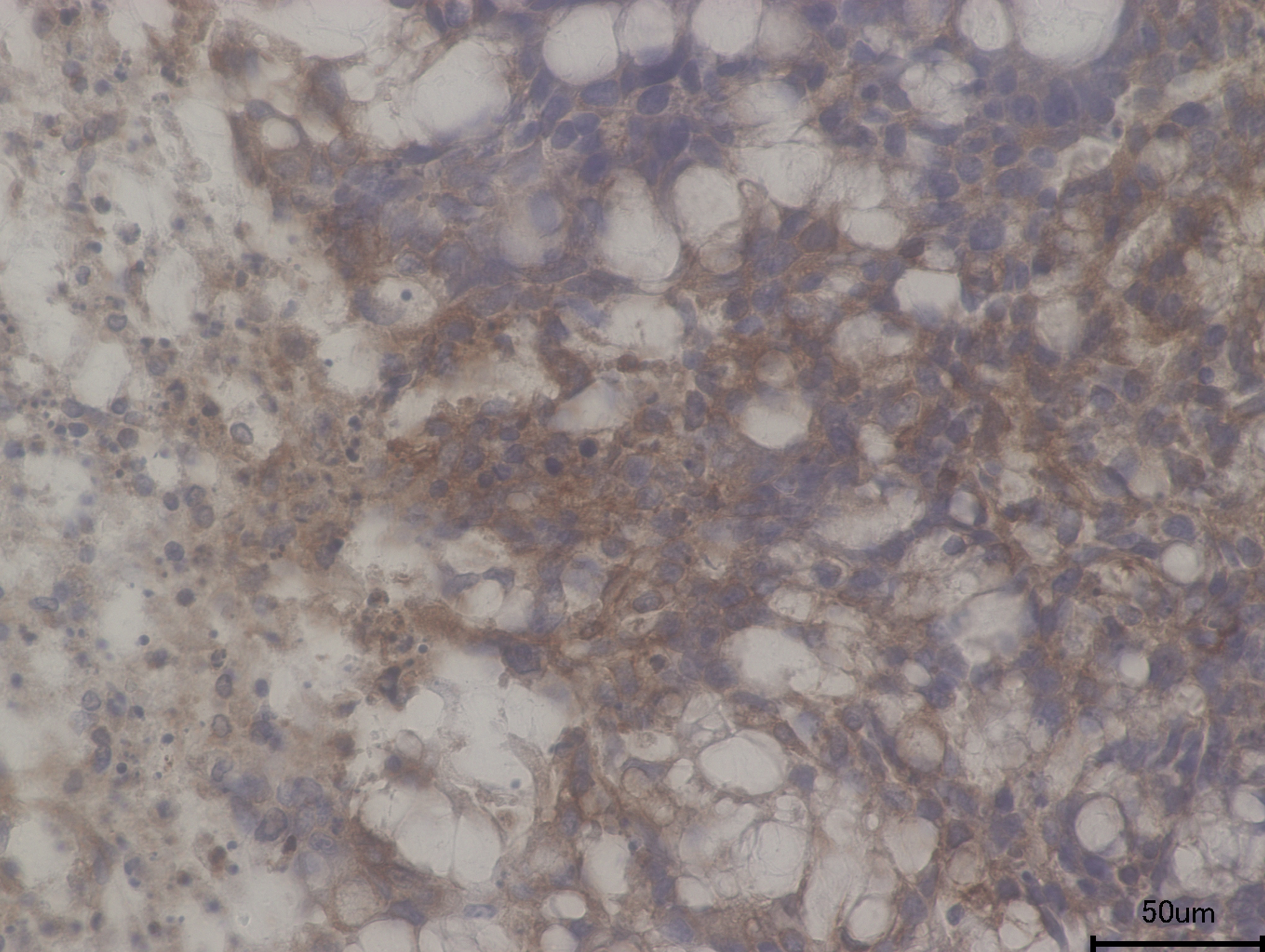

Supplement: Supplementary file 1 [file ijms-24-05797-s001.zip › Figure S2_CA9-CA12 immunohistochemistry - original pictures/LS174T/additional pictures/CA9 40x perinecrotic.jpg]

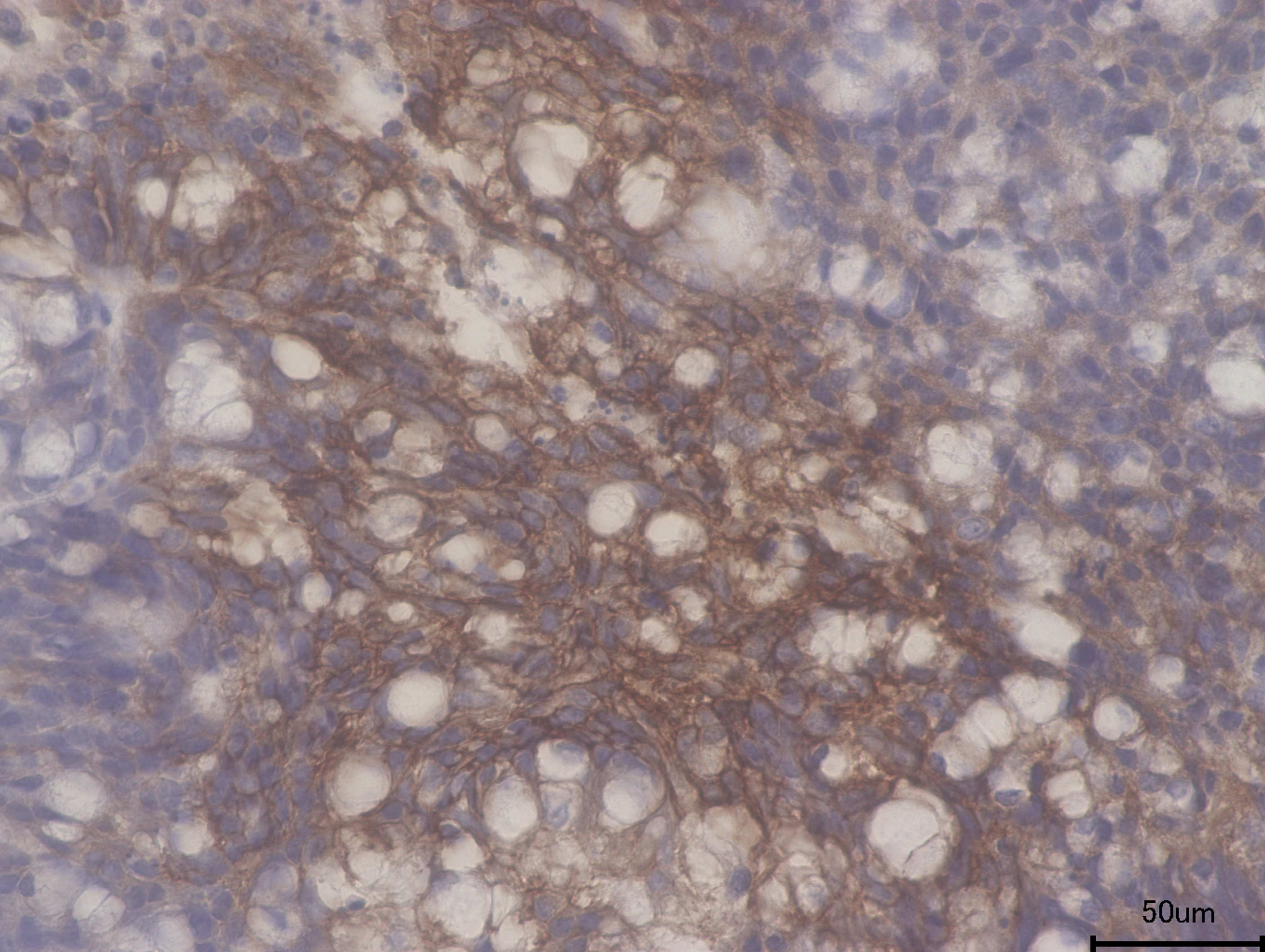

Supplement: Supplementary file 1 [file ijms-24-05797-s001.zip › Figure S2_CA9-CA12 immunohistochemistry - original pictures/LS174T/additional pictures/CA9 40x vital.jpg]

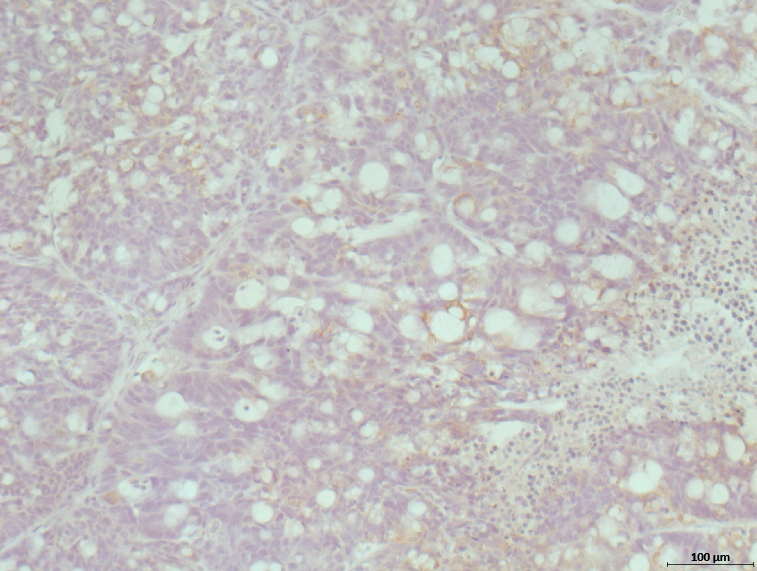

Supplement: Supplementary file 1 [file ijms-24-05797-s001.zip › Figure S2_CA9-CA12 immunohistochemistry - original pictures/LS174T/CA12 10x.tif]

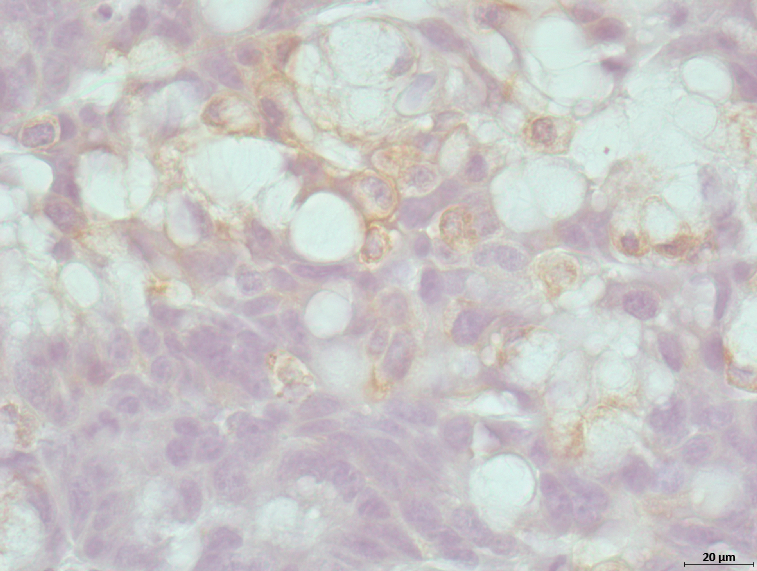

Supplement: Supplementary file 1 [file ijms-24-05797-s001.zip › Figure S2_CA9-CA12 immunohistochemistry - original pictures/LS174T/CA12 40x.tif]

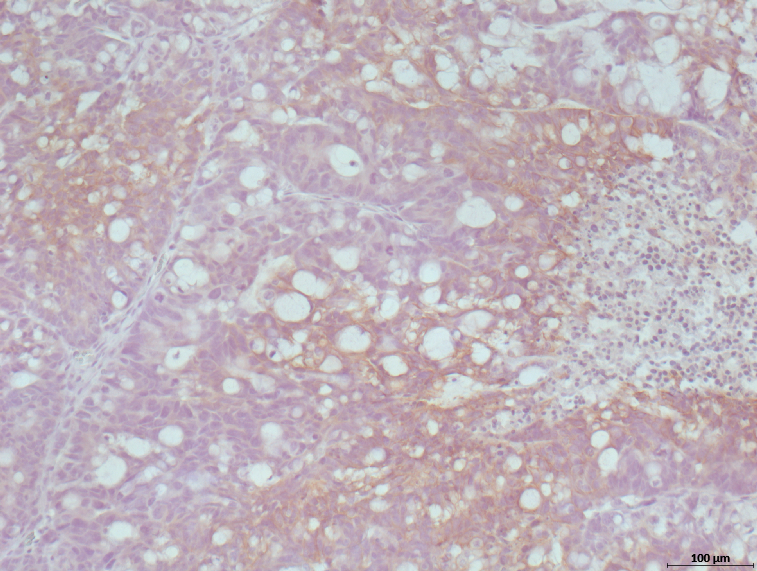

Supplement: Supplementary file 1 [file ijms-24-05797-s001.zip › Figure S2_CA9-CA12 immunohistochemistry - original pictures/LS174T/CA9 10x.tif]

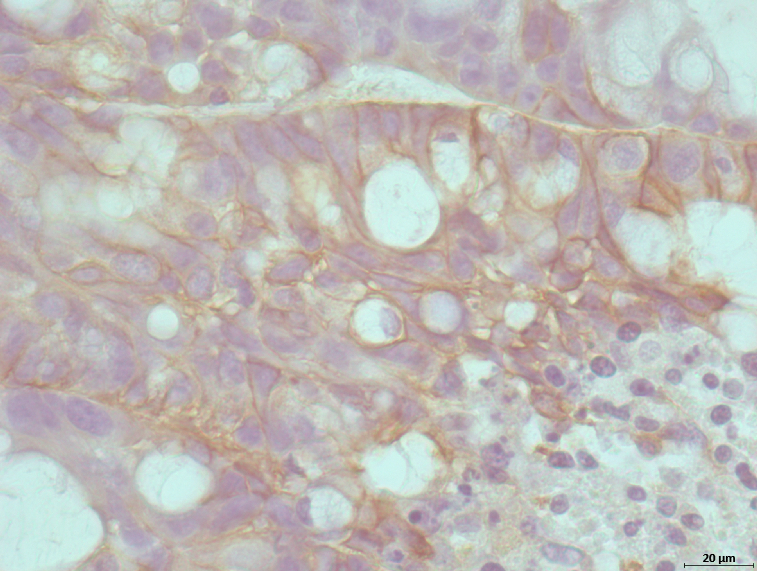

Supplement: Supplementary file 1 [file ijms-24-05797-s001.zip › Figure S2_CA9-CA12 immunohistochemistry - original pictures/LS174T/CA9 40x perinecrotic.tif]

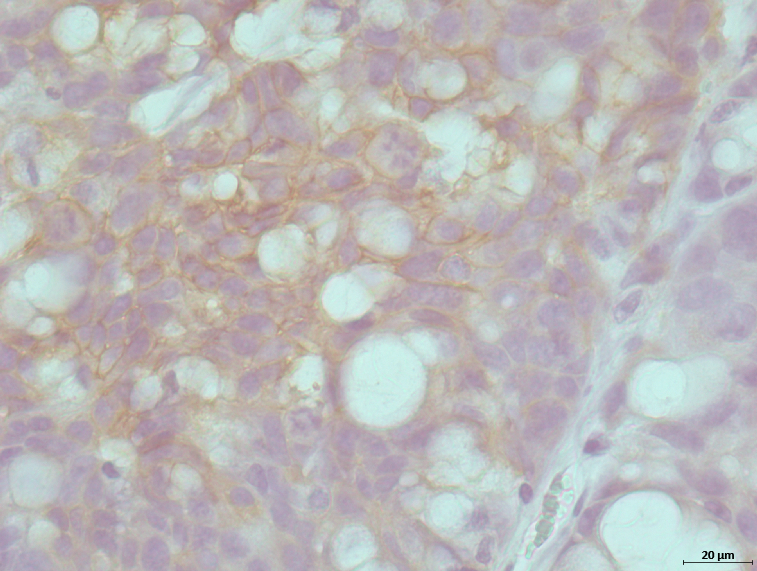

Supplement: Supplementary file 1 [file ijms-24-05797-s001.zip › Figure S2_CA9-CA12 immunohistochemistry - original pictures/LS174T/CA9 40x vital.tif]

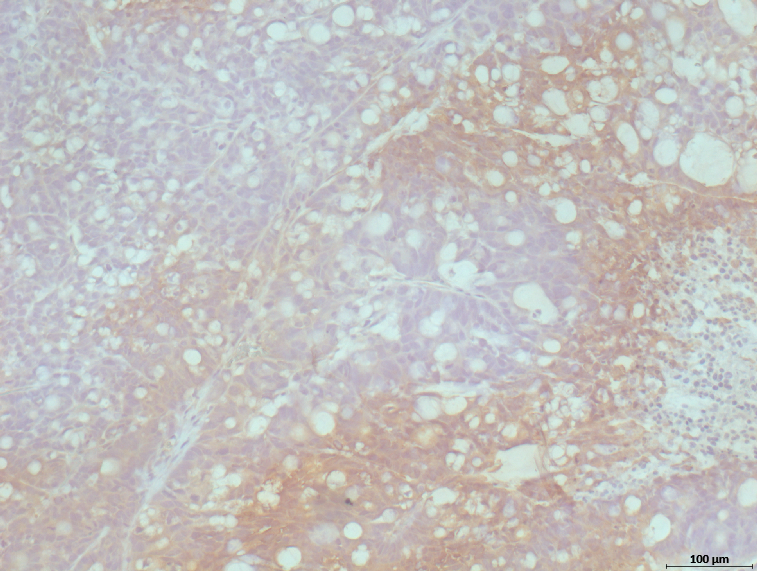

Supplement: Supplementary file 1 [file ijms-24-05797-s001.zip › Figure S2_CA9-CA12 immunohistochemistry - original pictures/LS174T/HP 10x.tif]

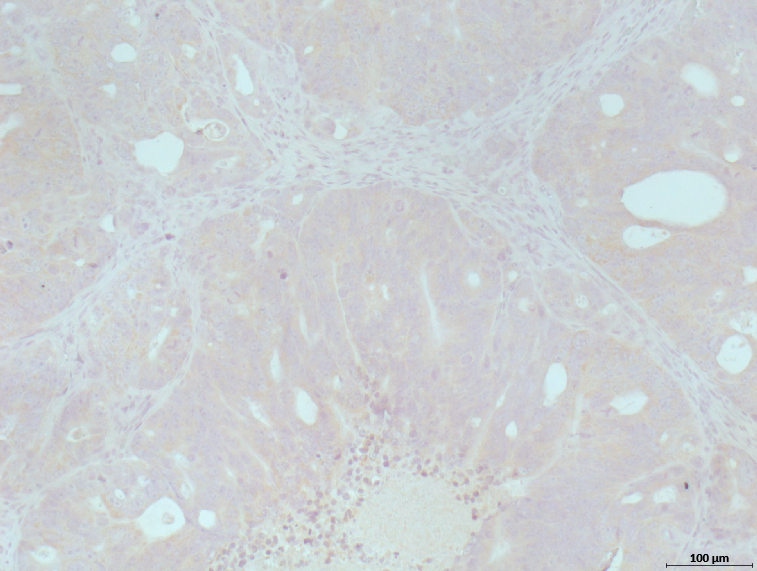

Supplement: Supplementary file 1 [file ijms-24-05797-s001.zip › Figure S2_CA9-CA12 immunohistochemistry - original pictures/SW1463/CA12 10x.tif]

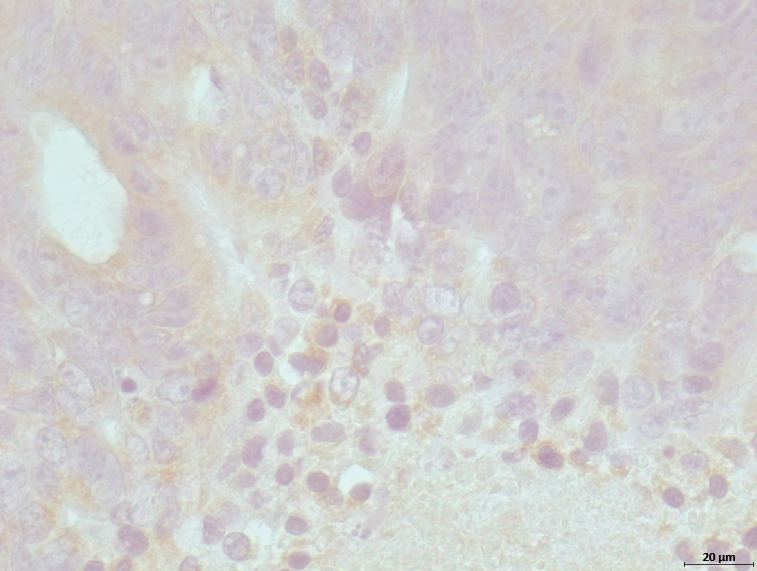

Supplement: Supplementary file 1 [file ijms-24-05797-s001.zip › Figure S2_CA9-CA12 immunohistochemistry - original pictures/SW1463/CA12 40x perinecrotic.tif]

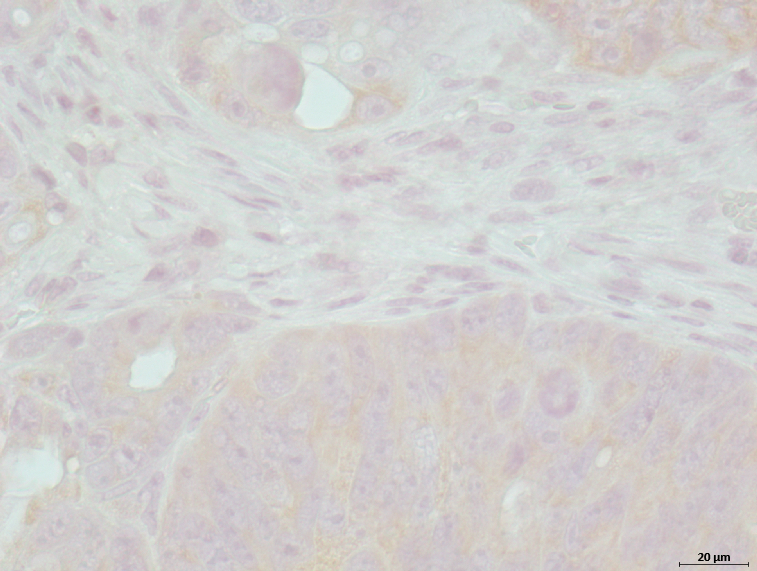

Supplement: Supplementary file 1 [file ijms-24-05797-s001.zip › Figure S2_CA9-CA12 immunohistochemistry - original pictures/SW1463/CA12 40x vital.tif]

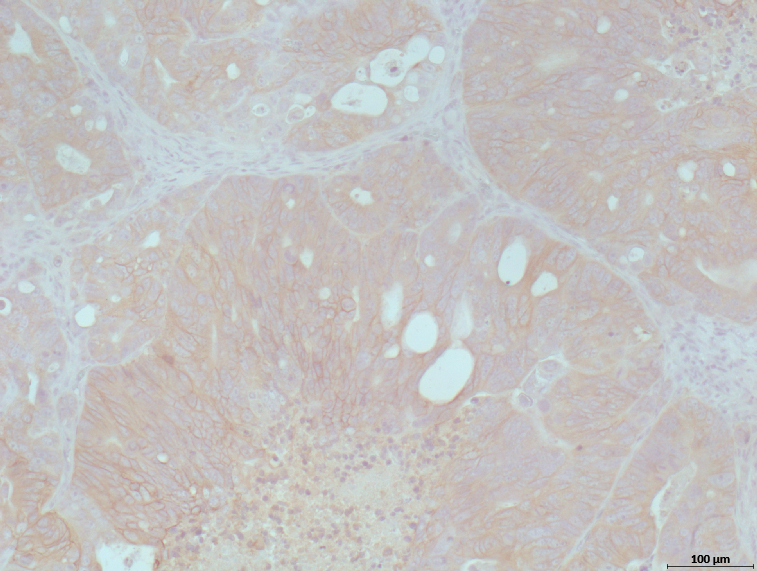

Supplement: Supplementary file 1 [file ijms-24-05797-s001.zip › Figure S2_CA9-CA12 immunohistochemistry - original pictures/SW1463/CA9 10x.tif]

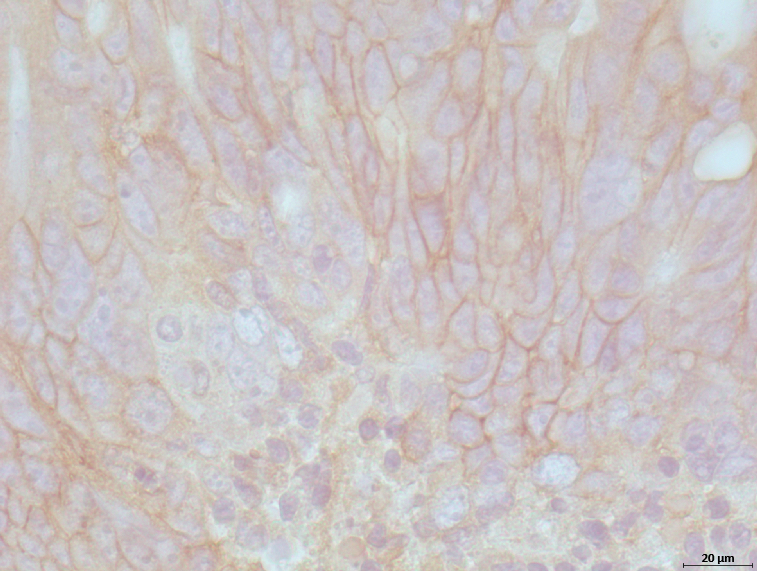

Supplement: Supplementary file 1 [file ijms-24-05797-s001.zip › Figure S2_CA9-CA12 immunohistochemistry - original pictures/SW1463/CA9 40x perinecrotic.tif]

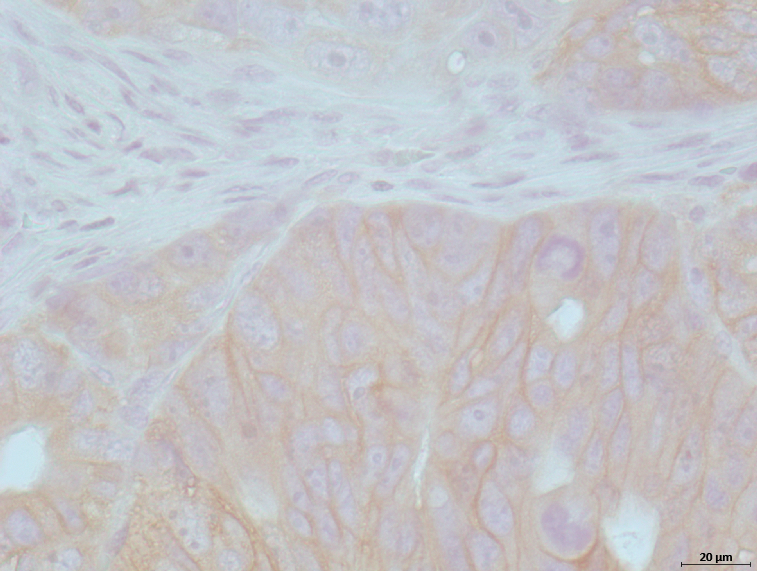

Supplement: Supplementary file 1 [file ijms-24-05797-s001.zip › Figure S2_CA9-CA12 immunohistochemistry - original pictures/SW1463/CA9 40x vital.tif]

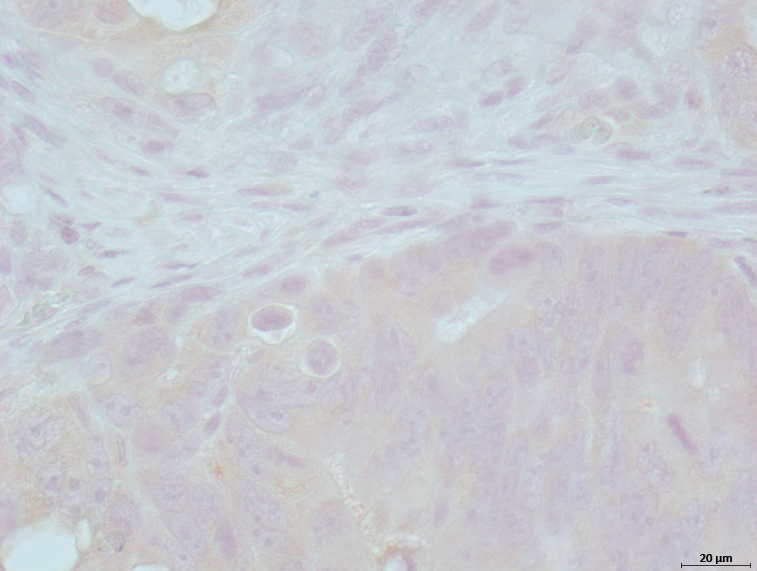

Supplement: Supplementary file 1 [file ijms-24-05797-s001.zip › Figure S2_CA9-CA12 immunohistochemistry - original pictures/SW1463/HP 40x vital.tif]

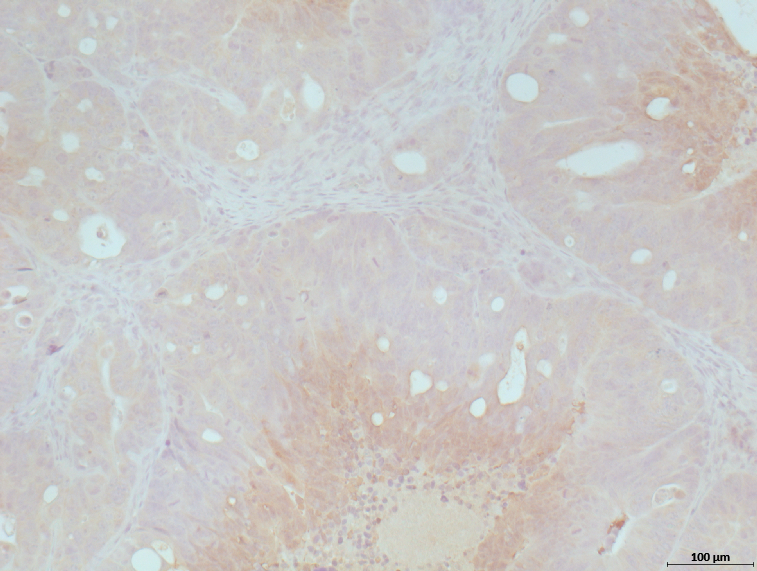

Supplement: Supplementary file 1 [file ijms-24-05797-s001.zip › Figure S2_CA9-CA12 immunohistochemistry - original pictures/SW1463/HP10x.tif]

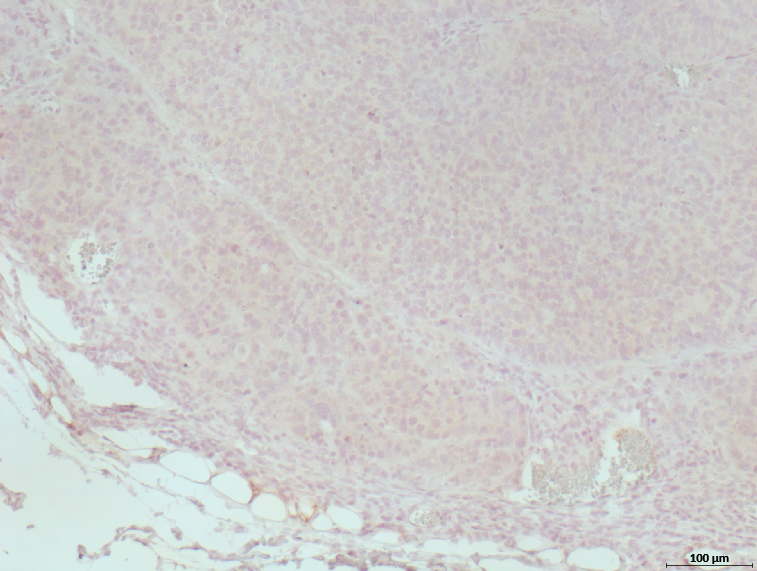

Supplement: Supplementary file 1 [file ijms-24-05797-s001.zip › Figure S2_CA9-CA12 immunohistochemistry - original pictures/SW480/CA12 10x.tif]

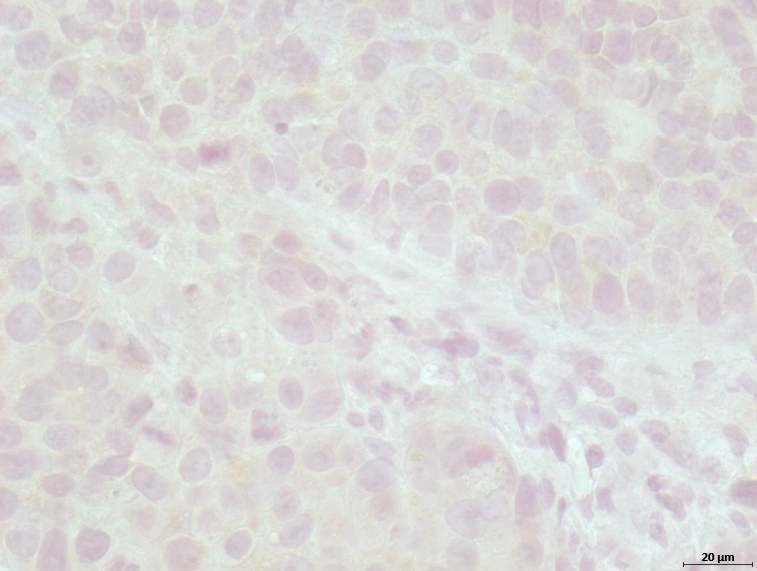

Supplement: Supplementary file 1 [file ijms-24-05797-s001.zip › Figure S2_CA9-CA12 immunohistochemistry - original pictures/SW480/CA12 40x.tif]

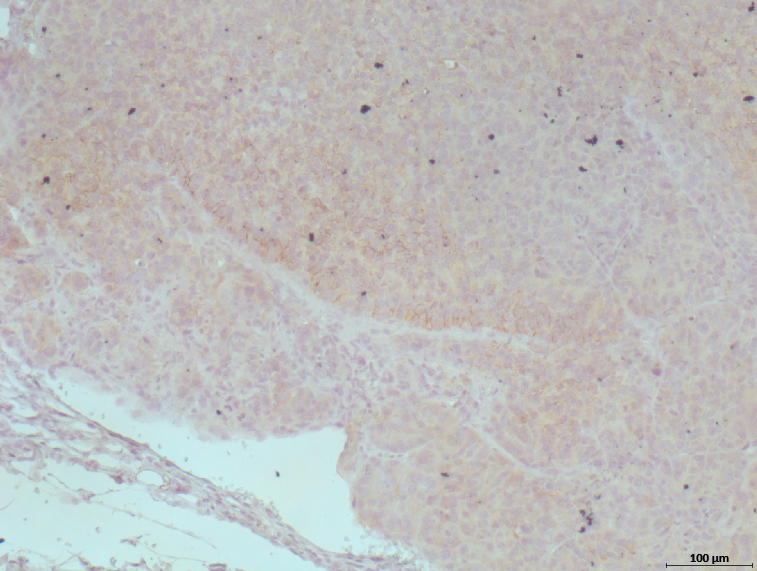

Supplement: Supplementary file 1 [file ijms-24-05797-s001.zip › Figure S2_CA9-CA12 immunohistochemistry - original pictures/SW480/CA9 10x.tif]

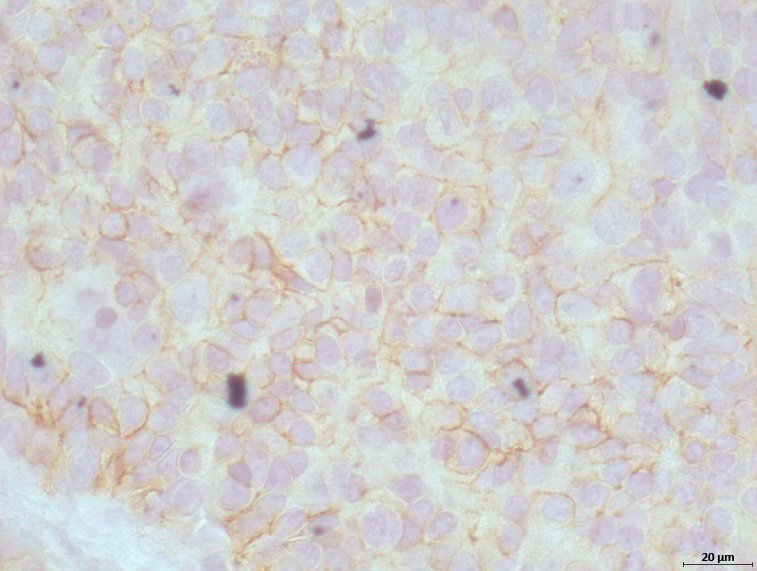

Supplement: Supplementary file 1 [file ijms-24-05797-s001.zip › Figure S2_CA9-CA12 immunohistochemistry - original pictures/SW480/CA9 40x.tif]

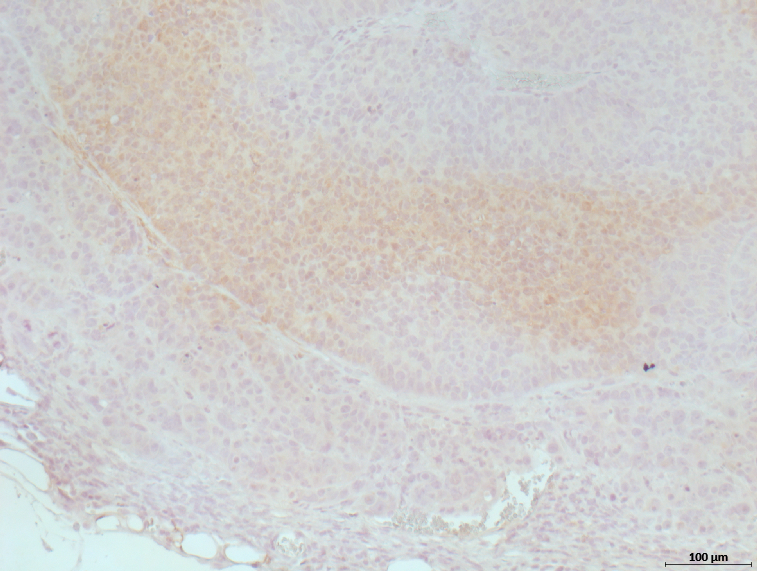

Supplement: Supplementary file 1 [file ijms-24-05797-s001.zip › Figure S2_CA9-CA12 immunohistochemistry - original pictures/SW480/HP 10x.tif]

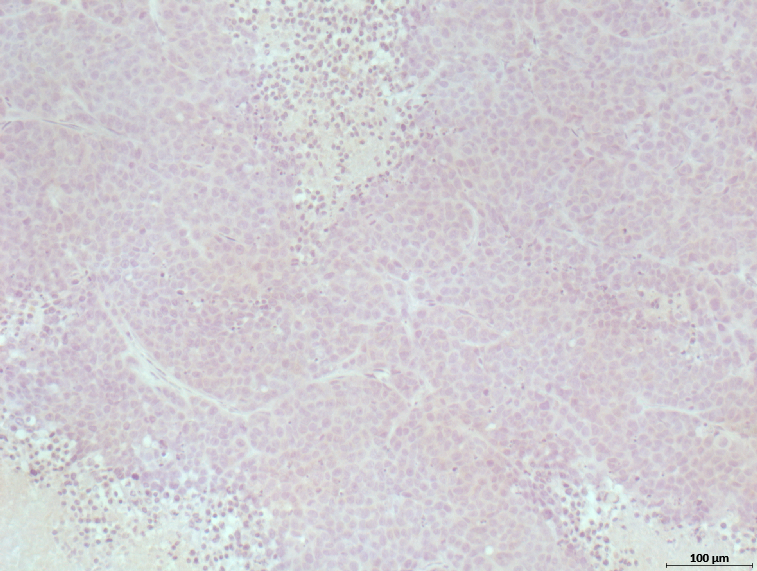

Supplement: Supplementary file 1 [file ijms-24-05797-s001.zip › Figure S2_CA9-CA12 immunohistochemistry - original pictures/SW48/CA12 10x.tif]

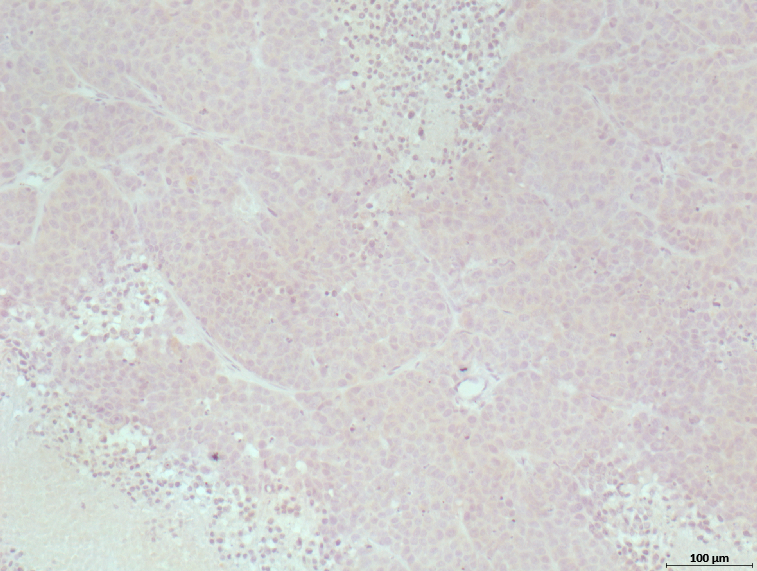

Supplement: Supplementary file 1 [file ijms-24-05797-s001.zip › Figure S2_CA9-CA12 immunohistochemistry - original pictures/SW48/CA9 10x.tif]

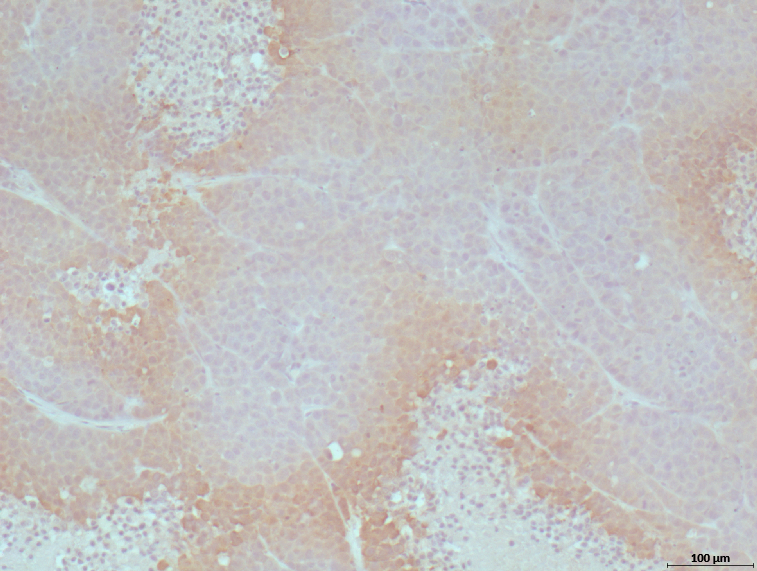

Supplement: Supplementary file 1 [file ijms-24-05797-s001.zip › Figure S2_CA9-CA12 immunohistochemistry - original pictures/SW48/HP 10x.tif]
